# Genome-wide DNA hypermethylation opposes healing in patients with chronic wounds by impairing epithelial-mesenchymal transition

**DOI:** 10.1172/JCI157279

**Published:** 2022-09-01

**Authors:** Kanhaiya Singh, Yashika Rustagi, Ahmed S. Abouhashem, Saba Tabasum, Priyanka Verma, Edward Hernandez, Durba Pal, Dolly K. Khona, Sujit K. Mohanty, Manishekhar Kumar, Rajneesh Srivastava, Poornachander R. Guda, Sumit S. Verma, Sanskruti Mahajan, Jackson A. Killian, Logan A. Walker, Subhadip Ghatak, Shomita S. Mathew-Steiner, Kristen E. Wanczyk, Sheng Liu, Jun Wan, Pearlly Yan, Ralf Bundschuh, Savita Khanna, Gayle M. Gordillo, Michael P. Murphy, Sashwati Roy, Chandan K. Sen

**Affiliations:** 1 Indiana University Health Comprehensive Wound Center, Indiana Center for Regenerative Medicine and Engineering, Department of Surgery, Indiana University School of Medicine, Indianapolis, Indiana, USA.; 2 Department of Surgery, The Ohio State University, Columbus, Ohio, USA.; 3 Sharkia Clinical Research Department, Ministry of Health, Zagazig, Sharkia, Egypt.; 4 Department of Biomedical Engineering, Indian Institute of Technology Ropar, Punjab, India.; 5 Department of Physics, The Ohio State University, Columbus, Ohio, USA.; 6 Center for Computational Biology and Bioinformatics (CCBB), Indiana University School of Medicine, Indianapolis, Indiana, USA.; 7 Comprehensive Cancer Center,; 8 Department of Chemistry & Biochemistry, and; 9 Division of Hematology, The Ohio State University, Columbus, Ohio, USA.

**Keywords:** Dermatology, Therapeutics, Epigenetics, Molecular biology

## Abstract

An extreme chronic wound tissue microenvironment causes epigenetic gene silencing. An unbiased whole-genome methylome was studied in the wound-edge tissue of patients with chronic wounds. A total of 4,689 differentially methylated regions (DMRs) were identified in chronic wound-edge skin compared with unwounded human skin. Hypermethylation was more frequently observed (3,661 DMRs) in the chronic wound-edge tissue compared with hypomethylation (1,028 DMRs). Twenty-six hypermethylated DMRs were involved in epithelial-mesenchymal transition (EMT). Bisulfite sequencing validated hypermethylation of a predicted specific upstream regulator TP53. RNA-Seq analysis was performed to qualify findings from methylome analysis. Analysis of the downregulated genes identified the *TP53* signaling pathway as being significantly silenced. Direct comparison of hypermethylation and downregulated genes identified 4 genes, *ADAM17*, *NOTCH*, *TWIST1*, and *SMURF1*, that functionally represent the EMT pathway. Single-cell RNA-Seq studies revealed that these effects on gene expression were limited to the keratinocyte cell compartment. Experimental murine studies established that tissue ischemia potently induces wound-edge gene methylation and that 5′-azacytidine, inhibitor of methylation, improved wound closure. To specifically address the significance of *TP53* methylation, keratinocyte-specific editing of *TP53* methylation at the wound edge was achieved by a tissue nanotransfection-based CRISPR/dCas9 approach. This work identified that reversal of methylation-dependent keratinocyte gene silencing represents a productive therapeutic strategy to improve wound closure.

## Graphical abstract

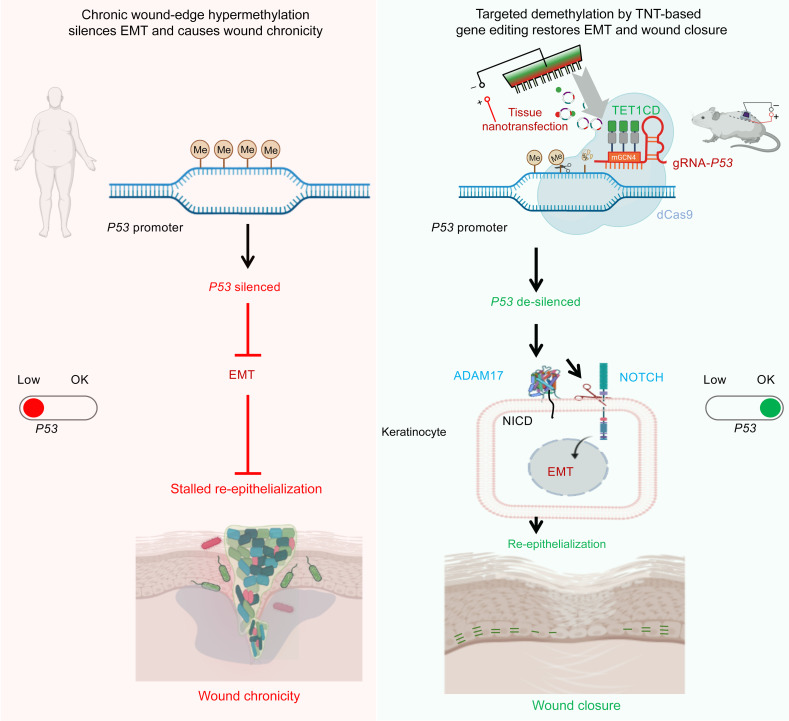

## Introduction

The extreme conditions that are characteristic of the chronic wound environment have no parallel within the live human body. Examples include the high burden of planktonic and biofilm microbial pathogens (1), sharply lower pH (2), abundance of dying and necrotic tissue (3), excessive ROS (4), unique biochemical *milieu*, including hyperactive enzymes (5), and hyperactive immune responses (6). Any parallel to the above-mentioned combinations within the live body would inevitably cause septic death (7). Although an integral component of the live body, the chronic wound microenvironment is compartmentalized, thus mitigating systemic complications. This anatomical isolation, contributed by underlying vasculopathies, allows the extreme chronic wound microenvironment to persist in the live human body for years without any imminent threat to patient survival in most cases. The viable chronic wound tissue surviving such extreme microenvironmental conditions is complicated by epigenetic gene silencing secondary to long-term hypoxia and infection (8–10). These conditions induce DNA hypermethylation to cause such gene silencing (11). Pharmacological reversal of such hypermethylation has been proven to be productive in rescuing tissue regeneration (12). Thus, the chronic wound niche offers an unparalleled opportunity to study the effects of an extreme biochemical microenvironment on epigenomic alterations and their significance (13). The significance of such a line of investigative pursuit is heightened by the fact that in the United States, 2% of the population is affected by chronic wounds (14). Because of the sharp escalation of the health care cost burden, rapidly growing aging population, and rapid rise in the incidence of diabetes and obesity, the burden of treating chronic wounds is growing rapidly (14). Molecular analyses of the wound tissue of patients with chronic wounds represents a powerful approach to identify clinically relevant therapeutic targets.

Cutaneous wounds close by reepithelialization. Onset of such restoration of the skin defect relies on loss of epithelial cell–cell adhesion; gain of apical-basal polarity; and gain of mesenchymal features, including enhanced motility and cytoskeletal rearrangement (15). Such epithelial-mesenchymal transition (EMT) is responsible for transient epithelial plasticity essential for initiation and progression of wound closure (16–18). Methylation-dependent silencing of EMT-inducing transcription factors can stall EMT (19). Wounds with defective EMT fail to close (20). Mice deficient in EMT marker vimentin or ZEB1 exhibit defects in wound reepithelialization caused by slow keratinocyte migration (17, 21). Blunted EMT, as well as defects in maturation and stratification of the neoepidermis, characterize such nonhealing wounds (22).

Ischemia, caused by underlying peripheral vascular disease, is a common complication of chronic wounds. Persistent inflammation and infection act as an oxygen sink and add to the severity of such ischemia (23). Studies of the heart (24) and kidney (25) have shown that ischemia can be a potent inducer of gene methylation. However, the significance of gene methylation in wound chronicity remains to be addressed. This work adopted a patient-based approach to catalog the incidence of gene methylation on a genome-wide basis and utilized that foundation to develop a mechanistic paradigm recognizing *TP53* methylation and gene silencing as a critical barrier to cutaneous wound EMT and healing.

## Results

### Comparative profiling of DNA methylation and mRNA expression in chronic wound-edge and unwounded human skin.

**Figure 1:**
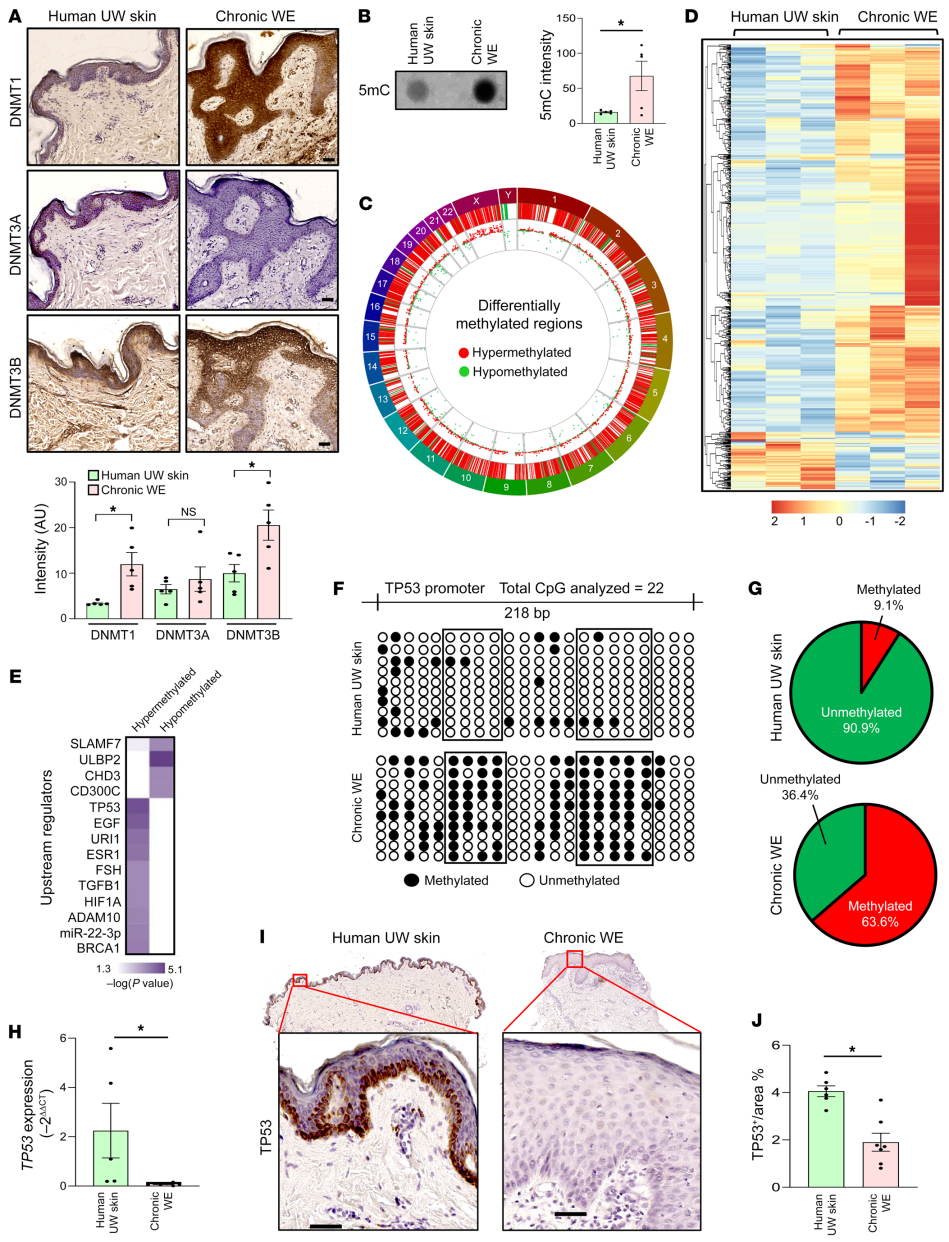
Increased global DNA methylation is strongly associated with human chronic WE tissue. (**A**) Representative IHC analysis of DNMT1 (top), DNMT3A (middle), and DNMT3B (bottom) in paraffin sections from human unwounded (UW) skin and chronic wound-edge (WE) tissue. Bottom panel represent the intensity analysis of the images. (Scale bar: 50 μm; *n =* 5; *P < 0.05, Student’s *t* test). (**B**) Dot blot analysis (left) and its intensity analysis (right) of 5-methylcytosine (5mc) in human chronic WE compared with UW skin (*n =* 5; *P < 0.05, Student’s *t* test). (**C**) Circos plot demonstrating the distribution of significant differentially methylated regions (DMRs) associated with 1 kb upstream and 1 kb downstream of Ref-Seq genes in human chronic WE. Chromosome number marked in the periphery. Red lines and dots represent hypermethylated loci, and green lines and dots represent hypomethylated loci in chronic WE. (**D**) Hierarchical clustering analysis of 4689 significant DMRs associated with Ref-Seq genes in chronic WE. (*n =* 3; FDR adjusted *P* < 0.05; 3661 hypermethylated and 1028 hypomethylated in chronic WE tissue) were obtained. (**E**) IPA upstream regulator analysis of methylation data identified TP53 to be the most significant hypermethylated upstream regulator in chronic WE. (**F**) Methylation status of a region of TP53 promoter (–1069 bp to –821 bp) analyzed through bisulfite sequencing (methylated CpG, black; unmethylated CpG, white) (*n =* 10 clones). (**G**) Distribution of methylated and unmethylated CpGs in TP53 promoter (human UW skin (top); chronic WE (bottom). (**H**) qRT-PCR analysis of *TP53* expression in human chronic WE and skin. (*n =* 5, 7; *P < 0.05, Student’s *t* test). (**I**) Representative IHC analysis of TP53 in sections from human UW skin and chronic WE and (**J**) intensity analysis of the images. (Scale bar: 50 μm; *n =* 6, 7; *P < 0.05, by Student’s *t* test). Data are presented as the mean ± SEM.

In the human skin, DNA methyltransferase (DNMT) 1 and 3 are primarily active in causing gene methylation (26). In the wound edge (WE) of patients with chronic wounds, expression of DNMT1 and 3B were elevated (Figure 1A). The functional significance of such elevation was validated by the observation that compared with unwounded (UW) human skin, chronic WE tissue was observed to be hypermethylated as evident by elevated 5-methylcytosine (5mC) (Figure 1B). The murine ischemic flap approach offers the opportunity to investigate DNA methylation as a function of graded ischemia (Supplemental Figure 1A; supplemental material available online with this article; https://doi.org/10.1172/JCI157279DS1). The simultaneous study of 5mC and 5hmC identified that DNMT expression and DNA hypermethylation were inducible by ischemia (Supplemental Figure 1, A–G). Comparison of whole-genome methylation status between human chronic WE tissue and UW skin revealed 4689 differentially methylated regions (DMRs) in the proximal promoters (1 kb upstream and 1kb downstream of TSS of Ref-Seq genes) of WE (Figure 1, C and D). Hypermethylation (3661 DMR) was more prominent than hypomethylation (1028 DMR) in human chronic WE tissue (Figure 1D). To address the functional significance of such changes in DMR, Ingenuity Pathway Analysis (IPA) was performed to identify the canonical pathways that were affected. EMT was found to be the most significant pathway enriched in hypermethylated DMR in chronic WE tissue (Supplemental Figure 1H). EMT-related genes were represented by 26 hypermethylated DMRs. Candidate genes included EMT inducers, such as *NOTCH* and *WNT*, and EMT-related transcriptional factors, such as *TWIST* and *FOXC2* (Supplemental Table 1 and Supplemental Figure 1I).

Upstream regulator analysis of the affected DMRs recognized *TP53* as a hypermethylated candidate gene primarily affected in WE tissue (Figure 1E and Supplemental Table 2). Candidate *TP53*, a known EMT regulator (27), was validated by bisulfite sequencing (Figure 1, F and G). Supplemental murine flap studies demonstrated that ischemia methylated *TP53* (Supplemental Figure 1, J and K). In humans, *TP53* hypermethylation in WE tissue was associated with downregulation of TP53 gene expression (Figure 1, H–J).

**Figure 2:**
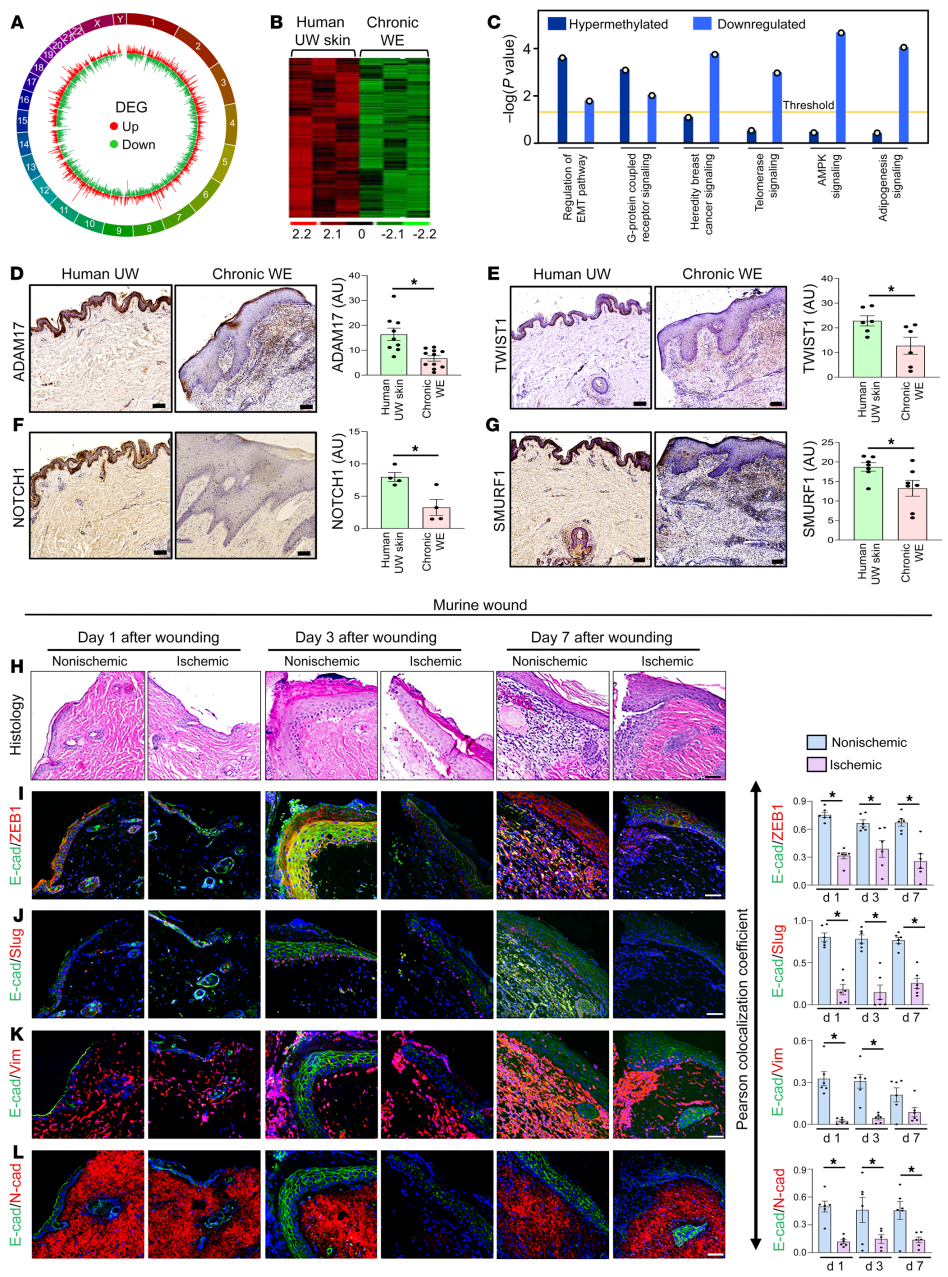
Increase in global DNA methylation represses EMT pathway in chronic WE tissue. (**A**) Circos plot demonstrating the distribution of significant differentially expressed genes (DEGs) obtained through RNA-Seq analysis in chronic WE. Chromosome number marked in the periphery. Red lines represent upregulated genes and green lines represent downregulated genes in chronic WE. (**B**) Hierarchical clustering analysis of significantly different genes in chronic WE. (**C**) Bar plot representing integration of hypermethylated genes (by MethylCap-Seq analysis) with downregulated genes (by RNA-Seq analysis) was performed to look for the common canonical pathways using the comparison analysis tool of IPA. The dot over each bar represents the –log(*P* value) of each pathway. (**D**) Representative IHC analysis of ADAM17, (**E**) TWIST1, (**F**) NOTCH1, and (**G**) SMURF1 in paraffin sections of human UW skin and chronic WE. Right panels represent the intensity analysis of the images. (Scale bar: 100 μm; *P < 0.05, Student’s *t* test, *n =* 4–11). (**H**) H&E staining and (**I**) representative IHC analyses for E-cadherin–ZEB1 colocalization, (**J**) E-cadherin–slug colocalization, (**K**) E-cadherin–vimentin colocalization, and (**L**) E-cadherin–N-cadherin colocalization in ischemic and nonischemic murine bipedicle wounds at different time points after wounding. Right panels represent the Pearson colocalization coefficient calculation at days 1, 3, and 7 after wounding. (Scale bar: 50 μm; *n =* 6, *P < 0.05). Data represented as mean ± SEM.

To determine the impact of hypermethylation on downregulation of gene expression, total RNA-Seq–based differential expression analysis was performed. Out of 57,825 annotated genes detected (includes 20,327 protein coding genes) (Figure 2A), 614 differentially expressed genes (DEGs) satisfied the comparison filtering criteria (*P* < 0.05; log_2_ fold change > ±1) (Figure 2B). Consistent with the genome-wide DNA hypermethylation status in WE tissue, hierarchical clustering analyses identified specific sets of downregulated DEGs (Figure 2B). Of the 614 DEGs, the TP53 signaling pathway was enriched for downregulated genes (Supplemental Figure 2A and Supplemental Table 3). IPA-based analysis of upstream regulator of genes that were downregulated predicted negative regulation of the *TP53* signaling pathway (Supplemental Figure 2B). Biological validation of these candidate genes was achieved using quantitative reverse transcription PCR (qRT-PCR) and IHC analyses of human chronic WE tissue. All candidates, with the exception of SIRT1, were validated (Supplemental Figure 2, C–N). Thus, these 2 sets of high-throughput genomic analyses, methylome and RNA-Seq, recognized the *TP53* pathway as being downregulated by hypermethylation in human WE tissue.

### DNA hypermethylation and downregulation of the EMT pathway.

To determine the candidate genes that share the same directionality both at methylation (hyper) levels and RNA expression (downregulation) levels, cross-comparison analysis of DMR-containing genes (from methylome analysis) with downregulated genes (from RNA-Seq) was performed. Given that the majority of the significant DEGs were downregulated, a search for candidates with hypermethylated DMRs was conducted. The comparison analysis module of IPA recognized the “regulation of EMT pathway” as the most enriched pathway whose genes shared similar directionality in terms of being hypermethylated and having lower expression (Figure 2C and Supplemental Table 4). The only other pathway recognized as being significant was “G-protein coupled receptor signaling.”

The contention that in chronic WE tissue, hypermethylated DMR downregulates DEGs representing the EMT signaling pathway was addressed (Supplemental Table 4). IHC analyses revealed that ADAM17, TWIST1, SMURF1, and NOTCH1 were downregulated in chronic WE tissue (Figure 2, D–G). The physiological relevance of ischemia, a common complication underlying chronic wounds, as downregulator of EMT genes was tested in an established murine model (28). IHC analyses of a panel of EMT-related proteins demonstrated a suppressive function of ischemia (Figure 2, H–L). Markers of WE EMT, i.e., colocalization of epithelial E-cadherin with mesenchymal markers (vimentin, N-cadherin, ZEB1, or Slug), was noted to be blunted in ischemic wounds (Figure 2, H–L). EMT is a property generally acquired by epithelial cells (29). To address the specific cell compartment affected, we turned toward single-cell RNA-Seq (scRNA-Seq).

### Patient-derived WE keratinocyte population.

**Figure 3:**
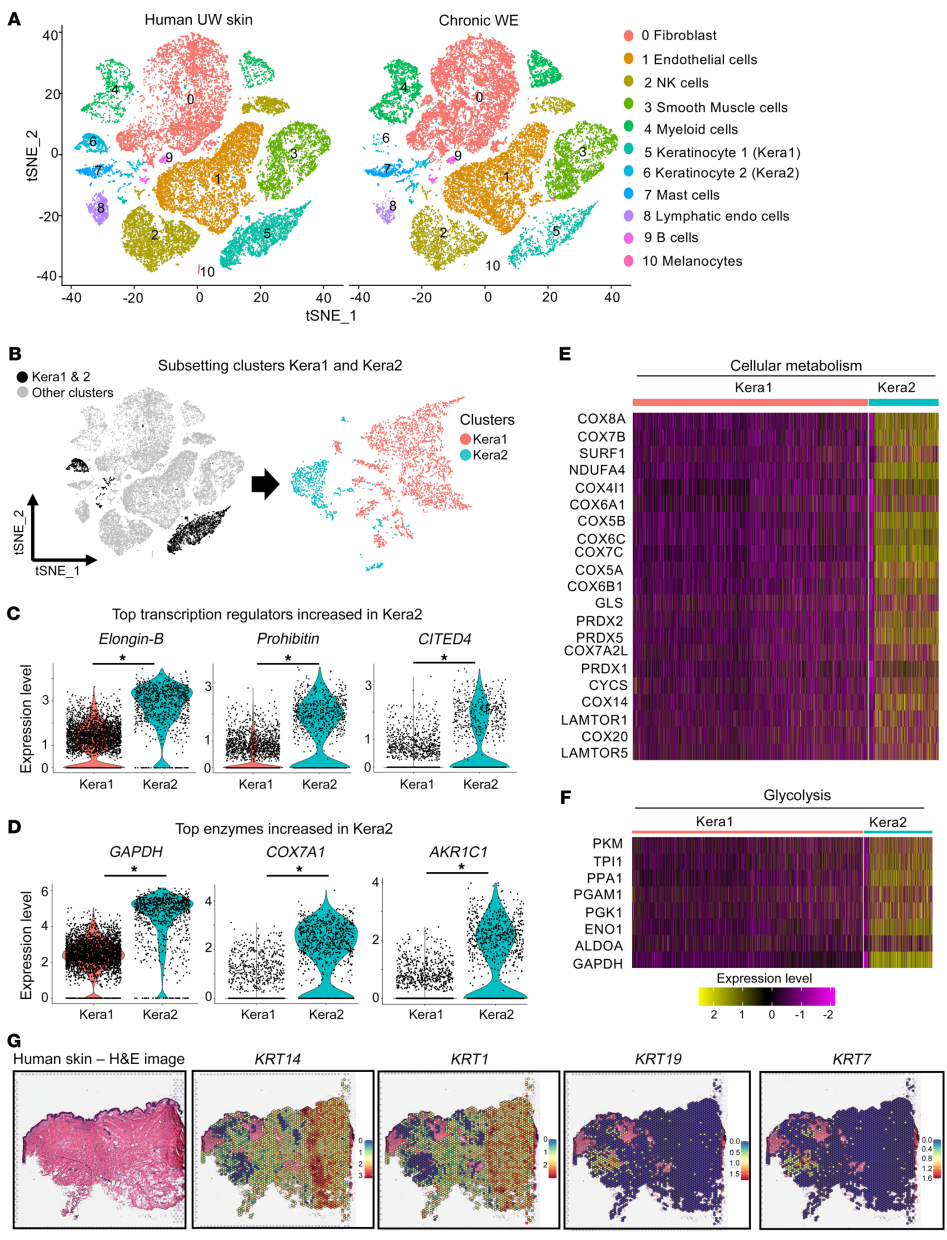
Single-cell RNA-Seq analysis identifies 2 epithelial clusters in human unwounded skin, one of which, high in metabolic genes, is diminished in chronic WE tissue. (**A**) tSNE (t-distributed stochastic neighbor embedding) plots showing single-cell transcriptomes of 25,561 cells from UW skin (obtained from 4 individuals) (left) and 25,168 cells from chronic WE (right) (obtained from 3 individuals) analyzed using the 10x Genomics platform. Unsupervised clustering revealed cellular heterogeneity with 11 distinct clusters of cells identified and color-coded. Each cell is represented as a dot. (**B**) tSNE clustering of the epithelial cells showing 2 identified keratinocytes, Kera1 (cluster 5) and Kera2 (cluster 6), in human UW skin. (**C**) Violin plots showing the expression level of the top 3 upregulated transcription regulators and (**D**) top 3 upregulated enzymes in the Kera2 cluster of human UW skin compared with the Kera1 cluster. (*adjusted *P* < 0.00001, Wilcoxon rank-sum test). (**E**) Heatmap showing the relative expression of genes involved in cellular metabolism in the 2 keratinocyte clusters (Kera1 and Kera2). (**F**) Heatmap showing the relative expression of genes involved in glycolysis in the 2 keratinocyte clusters (Kera1 and Kera2). (**G**) Spatial transcriptomics identified distinct localization of Kera1 (marked by high *KRT14* and *KRT1* expression) and Kera2 (marked by high *KRT19* and *KRT7* expression) in human UW skin through spatial feature plots. Scale bar for expression levels: *KRT14* (scale: 0–3), *KRT1* (scale: 0–2), *KRT19* (scale: 0–1.5), and *KRT7* (scale: 0–1.6). H&E staining of human skin section (left) was processed for Visium spatial gene expression analysis for classifying tissue based on mRNA levels. Further characterization of the Kera2 cluster is illustrated in Supplemental Figure 4A.

scRNA-Seq analysis was performed in 7 independent human samples, 3 of which were chronic WE and 4 represented reference UW skin. A data set from 67,040 cells from 7 samples was thus generated. After application of quality control measures, a total of 25,168 cells from chronic WE and 25,561 cells from UW skin were analyzed. Clustering analyses of these cells resulted in a t-distributed stochastic neighbor embedding (t-SNE) plot identifying 11 clusters with distinct expression profiles (Figure 3A and Supplemental Figure 3, A and B). These clusters were composed of 10 cell types present in human chronic WE tissue: fibroblasts 34.3%, endothelial cells 22.6%, smooth muscle cells 10.7%, NK cells 9.1%, myeloid cells 12.7%, keratinocytes 5.8%, mast cells 2.9%, B cells 1%, lymphatic endothelial cells 0.9%, and melanocytes 0.1% (Supplemental Figure 3C). The above-mentioned clusters had similar expression of housekeeping gene (β-actin) and contained cells from all the donors (Supplemental Figure 3D). In reference UW skin, based on DEGs, keratinocytes were detected in 2 clusters (5, Kera1; 6, Kera2). Among the keratinocyte-specific genes, Kera1 showed high abundance (more than log 2 folds) of *KRT1*, *KRT5*, *KRT14*, *KRT10*, *KRT16*, and *KRT6A*, representing the stratified epithelium (30). In contrast, Kera2 was characterized by high abundance of *KRT7*, *KRT8*, *KRT18*, and *KRT19*, which are known to be characteristic markers of nonstratified (simple) epithelium (30). Compositional analysis revealed substantial loss of the Kera2 subpopulation in the chronic WE tissue (*P* < 0.00001; χ^2^ with Yates correction = 187.98) (Figure 3A and Supplemental Figure 3C).

Further characterization of UW skin Kera1 and Kera2 was achieved by specifically subsetting clusters 5 and 6 (keratinocytes) only (Figure 3B). Comparison of these 2 clusters using a Wilcoxon rank-sum test resulted in the identification of 457 upregulated and 421 downregulated genes in Kera1 (Supplemental Table 5). Kera2 was identified to highly express transcription regulators Elongin-B, Prohibitin, and Cited4 (top 3 candidates), all known for their role in EMT (31–34) (Figure 3C). Furthermore, Kera2 showed high abundance of distinct metabolic enzymes, such as *GAPDH*, cytochrome c oxidase subunit isoform (*COX7A1*), and aldo-keto reductase 1C1 (*AKR1C1*) (35–37) (Figure 3D). Pathway analysis using the Reactome database (38) demonstrated enrichment of genes related to cellular metabolism and glycolysis in Kera2 (Figure 3, E and F). In UW skin, distinct spatial localization of Kera1 and Kera2 was visualized using the Visium spatial transcriptomics platform (39). Kera1 was marked as *KRT14*^+^*KRT1*^+^, while Kera2 was marked as *KRT19*^+^*KRT7*^+^ (Figure 3G). In UW skin, the spatial area marked by Kera2 was also enriched in metabolic genes like *GAPDH*; *CITED4*; *COX5B*, *6A1*, *7B*, *7C*, *8A*; *NDUFA4*; and *PRDX4* (Supplemental Figure 4A). In supplemental murine studies, Kera2 was detected as *KRT19*^+^*KRT7*^+^ cells as a function of wound healing (Supplemental Figure 4B). After injury, there was an increase in Kera2 cells in murine WE tissue on day 3 (Supplemental Figure 4B). This injury-dependent increase in Kera2 cells was blunted in ischemic wounds (Supplemental Figure 4B).

**Figure 4:**
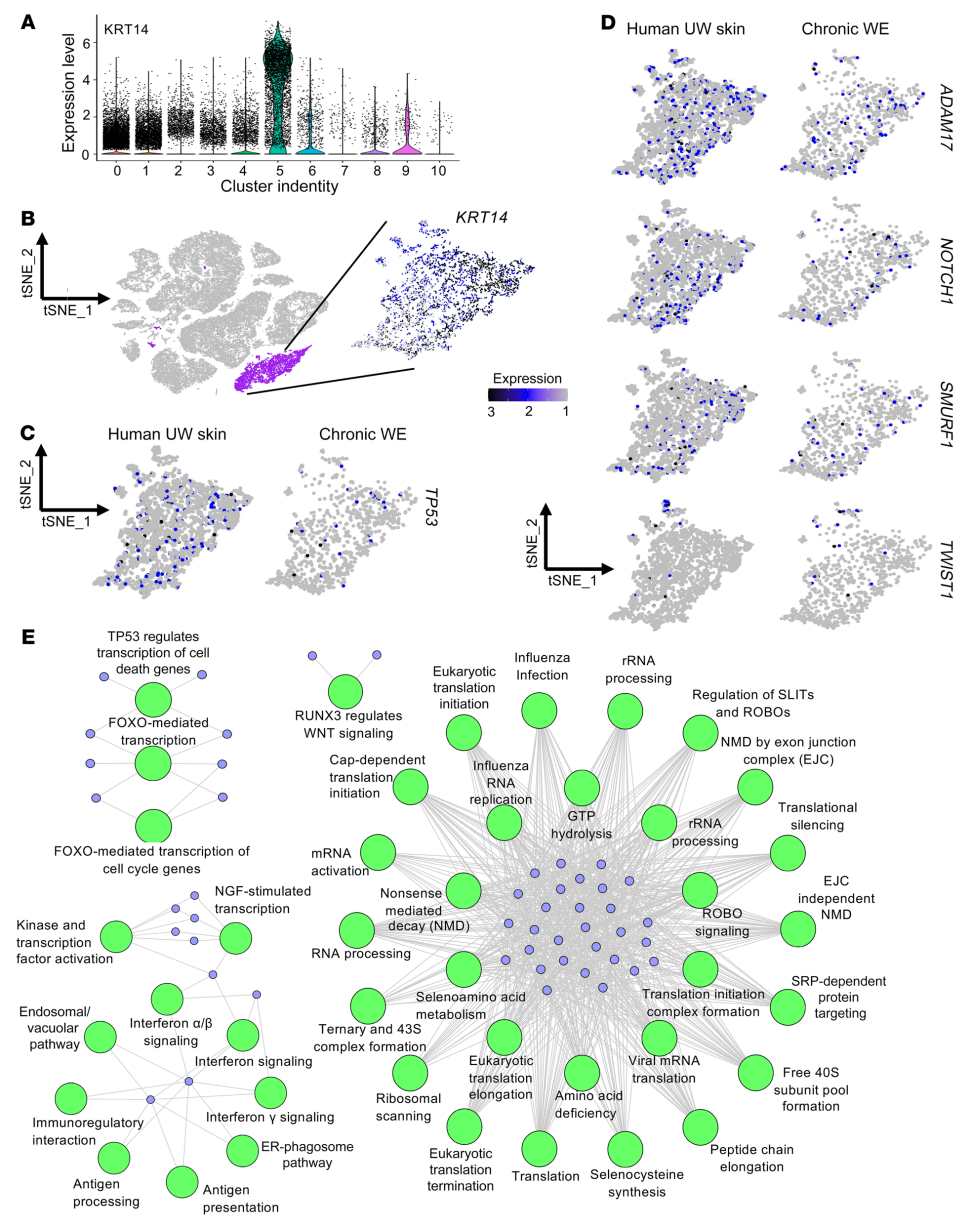
*KRT14*^+^ Kera1 cluster expresses low transcripts of identified EMT-related genes. (**A**) Violin plot showing expression of *KRT14* in the identified 11 clusters obtained through scRNA-Seq analyses. (**B**) tSNE plot with *KRT14*^+^ Kera1 (cluster 5) color-coded purple (left) and expression of KRT14 in the Kera1 cluster (right). (**C**) Expression level of *TP53* in Kera1 derived from human UW skin (left) and chronic WE (right). (*P* < 0.0001; χ^2^ test). (**D**) Expression levels of *ADAM17*, *NOTCH1*, *SMURF1*,and *TWIST1* in human UW skin and chronic WE. Scale bar for expression levels: scale 1–3. (*P* < 0.05, except for *TWIST1*; χ^2^ test). (**E**) Network of Reactome pathways enrichment of the downregulated pathways in Kera1 epithelial cells of chronic WE (CW) compared with human UW skin (adjusted *P* < 0.001 and at least 8% of the pathway genes found in the DEG). NGF, nerve growth factor; NMD, nonsense-mediated decay; EJC, exon junction complex; SRP, signal recognition particle; SLIT, slit guidance ligand 1; ROBO, roundabout.

Unlike Kera2, which was markedly depleted in chronic WE tissue, Kera1 was detected in both WE and UW skin samples. Thus, the classical *KRT14*^+^ Kera1 cluster was analyzed in chronic WE samples (1062 cells) with UW skin (3068 cells) as the reference (Figure 4, A and B). DEGs affected by WE hypermethylation, *TP53*, *ADAM17*, *NOTCH1*, *TWIST1*, and *SMURF1*, were studied in Kera1. All of these DEGs were substantially downregulated in Kera1 (Figure 4, C and D, and Supplemental Figure 5A). For example, while 5.9% of Kera1 cells of UW skin were *TP53*^+^, in chronic WE skin, only 2.5% of the cells were *TP53*^+^. Of note, such downregulation of DEGs was exclusively observed in the Kera1 cell population (Supplemental Figure 5B).

Unbiased differential analysis of Kera1 gene expression (Supplemental Table 6) identified 158 upregulated and 144 downregulated genes in human chronic WE compared with UW skin. Reactome pathway enrichment analysis of Kera1 DEGs of chronic WE tissue revealed that the downregulated genes were significantly associated with TP53-FOXO–mediated regulation of transcription, RNA processing, transcription or translation initiation, and transcription factor activation, among others (Figure 4E). The upregulated genes, however, were significantly associated with collagen degradation, degradation of extracellular matrix (ECM), keratinization, and ECM proteoglycans with other functional pathways (Supplemental Figure 5C). Among the other candidate genes involved in the classical NOTCH and WNT pathway, *HES1* and *WNT4* were hypermethylated and their expression downregulated in chronic WE tissue (Supplemental Figure 6, A and B, and Supplemental Figure 7, A and B). These scRNA-Seq data complemented methylome and bulk RNA-Seq data to recognize the impact of chronic WE methylation in the keratinocyte cell compartment.

Clinically, injuries to the skin cause acute wounds. These wounds are interpreted as chronic if not closed within 4 weeks of onset (40). Comparative studies of acute versus chronic wounds represent a powerful approach to understanding mechanisms underlying wound chronicity. Published scRNA-Seq data from day 7 human acute wounds were thus analyzed (41). Keratinocyte (KRT14^+^) populations of acute wounds (355 cells) and uninjured skin (314 cells) were compared (Supplemental Figure 8A). Unlike observations in the chronic WE tissue (Figure 3A), depletion of skin KRT19^+^ Kera2 cells was not observed in acute wounds (71.34% in uninjured skin versus 74.65% in day 7 acute wounds) (Supplemental Figure 8B). In addition, unlike in chronic WE tissue, the downregulation of TP53, ADAM17, NOTCH1, TWIST1, and SMURF1 was not observed in acute wounds compared with the corresponding uninjured skin (Supplemental Figure 8C). Next, pathway enrichment was performed using the Reactome database using only significant genes with adjusted *P* value less than 0.05 and log_2_ fold change ± 0.5 (Supplemental Figure 8, D and E). In contrast to chronic WE keratinocytes, where downregulated genes were associated with TP53-FOXO–mediated regulation of transcription (Figure 4E), acute wound keratinocytes displayed downregulated iron uptake and transport pathways (Supplemental Figure 8E). Among the top significant upregulated pathways identified in day 7 acute wound keratinocytes were keratinization and formation of cornified envelope, indicative of an active reepithelialization process (Supplemental Figure 8E).

### TP53 methylation hinders human keratinocyte migration.

**Figure 5:**
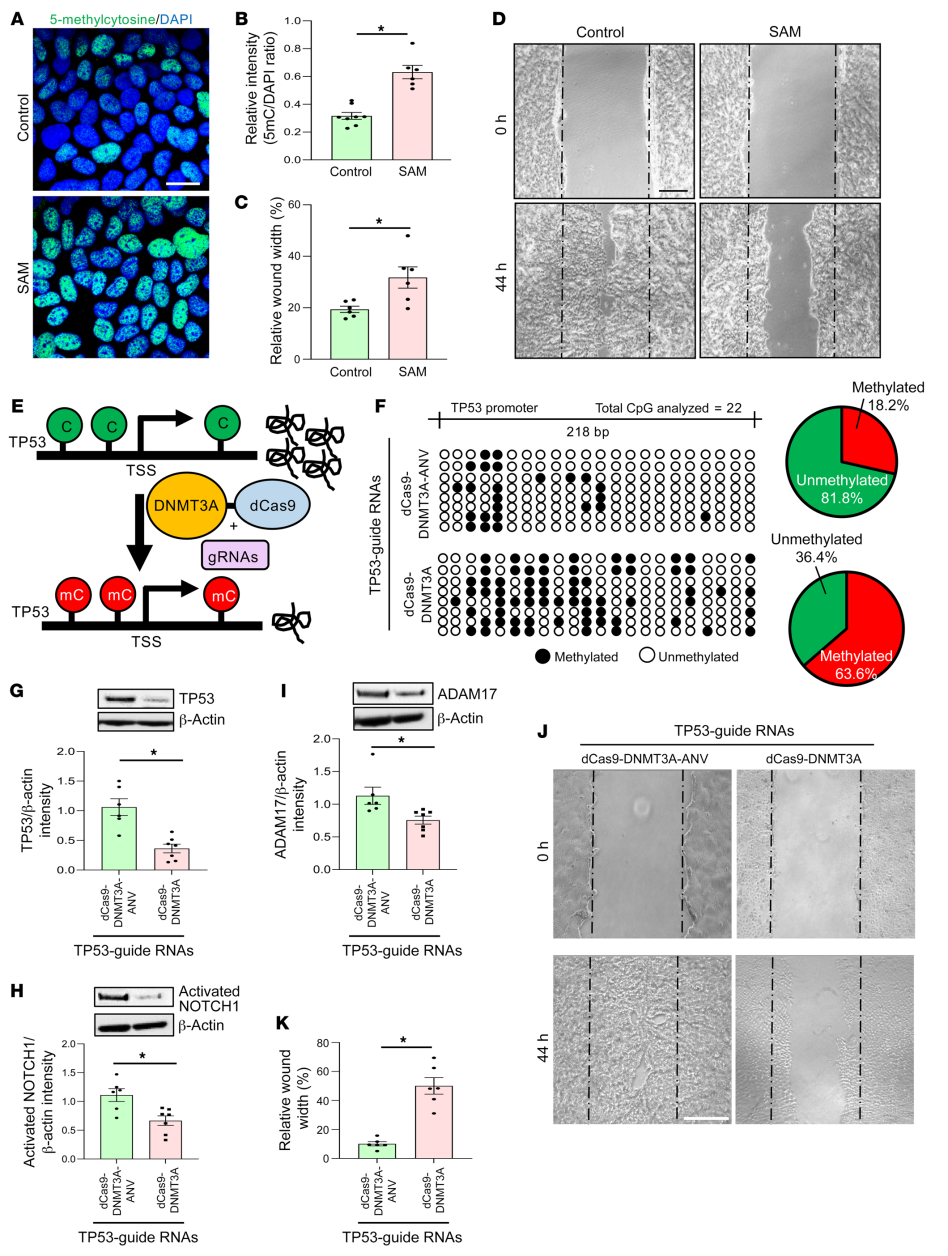
TP53 methylation hinders human keratinocyte migration. (**A**) Immunofluorescence analysis of 5-methylcytosine (5mc) in keratinocytes (HaCaT cells) exposed to vehicle control or S-adenosylmethionine (SAM) (80 μM, 48 h) and (**B**) their intensity analysis. DAPI intensity was used for normalization. (Scale bar: 20 μm; *n =* 6–8; *P < 0.001, Student’s *t* test). (**C**) Quantitation and (**D**) representative images of scratch-wound migration assay of keratinocytes. Scratch wound migration was performed after 48 hours of pretreatment with vehicle control or SAM (80 μM) and followed for 44 hours after wounding. (Scale bar: 200 μm; *n =* 6; *P < 0.05, Student’s *t* test). (**E**) Schematic diagram showing DNA methylation strategy of TP53 promoter mediated by dCas9-DNMT3A. Transfection efficiency of GFP-labeled plasmids shown in Supplemental Figure 9K through flow cytometry. (**F**) Left: schematic diagram of TP53 promoter (–1069 bp to –821 bp) analyzed through bisulfite genomic sequencing of DNA. Diagrammatic representation of the promoter methylation status shown (methylated CpG, black; unmethylated CpG, white). Right: Venn diagram showing the distribution of methylated and unmethylated TP53 promoter in HaCaT cells transfected with control (dCas-9-DNMT3A-ANV) (top) and in dCas-9-DNMT3A (bottom) in presence of TP53-guide RNAs. (**G**) Western blot analysis showing the expression of TP53, (**H**) activated NOTCH1, and (**I**) ADAM17 in HaCaT cells transfected with dCas-9-DNMT3A-EGFP or control (dCas-9-DNMT3A-EGFP-ANV) in presence of TP53-guide RNAs. Data expressed as fold change; β-actin used as loading control. (*n =* 6, 7; *P < 0.05, Student’s *t* test). Data represented as mean ± SEM. (**J**) Representative images and (**K**) quantitation of scratch-wound migration assay of HaCaT keratinocytes. Scratch-wound assay was performed after 48 hours of transfection with dCas-9-DNMT3A or control (dCas-9-DNMT3A-ANV) in presence of TP53-guide RNAs and followed for 44 hours after wounding. (*n =* 6; *P < 0.05, Student’s *t* test). Data represented as mean ± SEM.

Keratinocyte migration is required for wound reepithelialization and closure. The significance of DNA methylation on keratinocyte migration was tested in the presence of S-adenosyl methionine (SAM), a universal methyl donor (42). Exogenous SAM triggers DNA hypermethylation in the presence of DNMT (43). SAM pretreatment caused DNA hypermethylation of human keratinocytes as determined by increased abundance of 5mC (Figure 5, A and B). Cell migration was significantly blunted in SAM-treated cells (Figure 5, C and D). This inhibitory effect on migration was associated with downregulation of integrins (ITGA1, 4 and 5) and MMP1 (Supplemental Figure 9A). SAM-dependent DNA hypermethylation downregulated TP53 expression. This downregulation was associated with marked hypermethylation of the *TP53* promoter as determined by bisulfite sequencing (Supplemental Figure 9, B and C). Protein expression of other TP53-related EMT regulators, ADAM17 and activated NOTCH1, was also downregulated in SAM-treated keratinocytes, indicating coregulation of a broader EMT pathway in these cells (Supplemental Figure 9C).

SAM is a pharmacological methylating agent of choice that is known for its efficacy (44). In the interest of rigor, it is important to address possible epigenetic effects of SAM in any given experimental system that are independent of SAM as a methylating agent. In this work, SAM treatment did not affect H3K4 histone methylation but attenuated total H3 histone acetylation (Supplemental Figure 9, D and E). This effect of SAM was associated with increased levels of HDAC1 (45) (Supplemental Figure 9F). Follow-up studies were conducted with genetic and pharmacological inhibitors of DNMT as well as HDAC to determine the role of SAM-induced methylation on keratinocyte migration. The arresting effect of SAM on cell migration was abolished under conditions of inhibited DNMT (Supplemental Figure 9, G and H). Inhibition of HDAC1, however, did not rescue cell migration after SAM treatment (Supplemental Figure 9, I and J). These experiments established that the inhibitory effect of SAM on keratinocyte migration was DNA methylation dependent and independent of the effect of SAM on gene acetylation.

To determine the specific significance of *TP53* methylation, a targeted CRISPR/Cas9-based approach to induce *TP53* promoter methylation was adopted (Figure 5E). Catalytically inactive Cas9 (dCas9) was fused with the catalytic domain of DNMT3A (amino acids P602-V912) (46), known to be responsible for *de novo* DNA methylation. Targeted inactive construct (dCas9-DNMT3A-ANV) was used as a negative control. This catalytically inactive DNMT3A was generated by site-directed mutagenesis of the active site motif ENV to ANV (E756A) (46, 47). To test whether the downregulation of TP53 gene expression is indeed dependent on the site-specific DNA methylation, we targeted the dCas9-DNMT3A system using 5 sgRNAs designed to target the *TP53* promoter and its coding region. The confirmation of hypermethylation of the promoter (−1069 to −821 site) was achieved through bisulfite sequencing (Figure 5F). Overall, the percentage of CpG methylation of dCas-9-DNMT3A–transfected cells with *TP53* targeting sgRNAs was 63.6%, whereas cells transfected with catalytically inactive dCas-9-DNMT3A-ANV had a methylation percentage of 18.2% in the same region (Figure 5F and Supplemental Figure 9K). Analysis of TP53 expression by Western blot indicated an approximately 60% decrease in TP53 protein expression after targeting with dCas9-DNMT3A (Figure 5G). Functionally, such targeted methylation of *TP53* significantly inhibited keratinocyte migration (Figure 5, J and K). In addition, *TP53* methylation lowered the expression of ADAM17 and activated NOTCH1 (Figure 5, H and I).

### Correction of hypermethylation improves ischemic wound closure.

**Figure 6:**
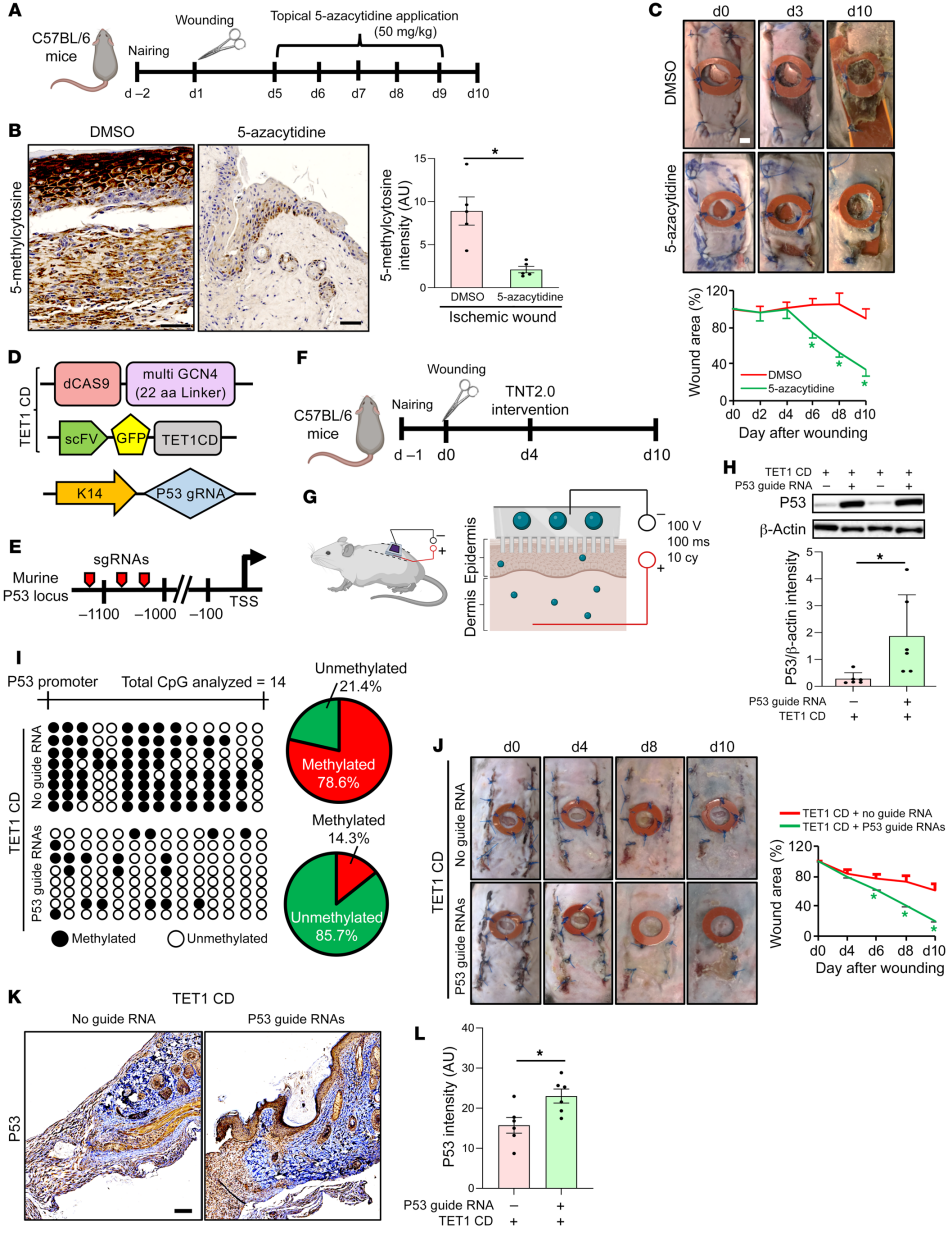
Correction of P53 hypermethylation improves ischemic wound closure. (**A**) Topical delivery of 5-azacytidine to murine ischemic bipedicle wounds. (**B**) Representative IHC and intensity analysis (right panel) of 5-methylcytosine in ischemic wounds treated with either vehicle control or 5-azacytidine. (Scale bar: 50 μm; *n =* 5; *P < 0.05, Student’s *t* test). (**C**) Wound closure determined by digital planimetry (top). Data presented as percentage of wound area (bottom). *n =* 7, 8, *P < 0.05 (Student’s *t* test). Data represented as mean ± SEM. (**D**) Vector components used for targeted demethylation of P53 promoter in keratinocytes using CRISPR/dCas9 approach. Keratinocyte targeting was achieved by KRT14 promoter–driven guide RNAs. (**E**) The mouse P53 promoter locus used for demethylation events. The locations of the targets (1–3) for sgRNAs are indicated by red pointers. (**F**) Topical delivery of TET1 CD and targeted sgRNAs to the ischemic wound employing tissue nanotransfection (TNT2.0) technology. (**G**) Schematic diagram of the TNT process. (**H**) Western blot analysis showing the expression of P53 in bipedicle ischemic wounds of mice nanotransfected with TET1CD and peptide repeat in presence or absence of KRT14 promoter–driven P53 gRNA targets. Data expressed as fold-change using β-actin as loading control. (*n =* 6; *P < 0.05, Student’s *t* test). (**I**) Demethylation activity was measured by bisulfite sequencing of murine P53 promoter region (mm10_chr11:69,578,954-69,579,215). (**J**) Wound closure was monitored at different days after wounding in bipedicle ischemic wounds of mice subjected to TNT by digital planimetry (left). Data presented as percentage of wound area (right). *n =* 8, *P < 0.05 (Student’s *t* test). (**K**) Representative IHC analysis of P53 in ischemic wounds subjected to TNT. (**L**) Intensity analysis of the images. (Scale bar: 100 μm; *n =* 6; *P < 0.05, Student’s *t* test). nairing, hair-removal technique using a depilatory agent (Nair, Church and Dwight).

An established murine model of ischemic wound healing was studied (Figure 6A) (28). Characterization of wound tissue ischemia was performed employing laser speckle imaging (Supplemental Figure 10, A and B). Ischemia increased methylation levels, as evident by increased levels of 5mC (Figure 6B). This was associated with downregulated expression of the EMT regulators TP53, ADAM17, and NOTCH1 (Supplemental Figure 10C). The functional significance of ischemia-induced methylation on wound closure was tested in a series of rescue experiments, the first of which utilized topical application of 5-azacytidine (5-aza), a DNMT inhibitor (Figure 6B). Wound closure, compromised by ischemia (Figure 6C), was rescued in response to topical 5-aza treatment beginning at day 5 but not at days 0 or 7 (Figure 6C, Supplemental Figure 10, D and E, and Supplemental Figure 11, A–H). Such improvement was associated with the rescue of all of the EMT regulators and an increase in the *KRT19*^+^*KRT7*^+^ Kera2 cell population at the ischemic WE (Supplemental Figure 10C and Supplemental Figure 12A). While investigating the effect of 5-aza on the non-keratinocyte compartment, no change in Col1A2^+^ collagen content was observed after 5-aza treatment (Supplemental Figure 12B). Interestingly, the abundance of myeloid cells, high in both human and murine ischemic wounds, was reduced at the murine WE in response to 5-aza treatment (Supplemental Figure 12C and Supplemental Figure 13, A–D). Of note, 5-aza–mediated rescue of wound closure was limited to the TP53-dependent EMT pathway, including ADAM17 and NOTCH1 (Supplemental Figure 10C). Both ADAM17 and NOTCH1 were observed to play an active role in the pathway leading to ischemic wound closure (Supplemental Figure 14, A–N).

To specifically address *TP53* demethylation as a strategy to treat ischemic wounds, a CRISPR/dCas9-based approach employing keratinocyte-specific guide RNAs was tested. The system contained an inactive Cas9 nuclease (dCas9) along with a plasmid-expressing catalytic domain at the C-terminus of TET1 (TET1 CD) (48). To increase the efficiency of targeted demethylation, a previously described dCas9-SUperNova Tagging (SunTag) with modified linker length to 22 amino acids was adopted (48–50). This approach was aimed at demethylating the promoter region of the *TP53* gene in the keratinocyte (KRT14^+^) compartment of ischemic wounds (Figure 6, D and E). Electrophoretic tissue nanotransfection (TNT; refs. 51, 52) allowed direct cytosolic delivery of demethylation cocktail (Figure 6, F and G, and Supplemental Figure 15A). First, the targeted delivery of guide RNAs to keratinocytes was validated using flow cytometry (Supplemental Figure 15B). Subsequently, the demethylation of the *TP53* promoter was confirmed (Figure 6, H and I). Successful demethylation of the *TP53* promoter rescued the expression of EMT regulators TP53, ADAM17, and NOTCH1 and promoted ischemic wound closure (Figure 6, H and J–L, and Supplemental Figure 15, C and D).

## Discussion

A direct role of microenvironment on epigenetic modifications has been established in tumor biology (53). This work presents the first evidence, to our knowledge, that human chronic WE tissue, nested in an extreme and unique microenvironment, is subject to genome-wide hypermethylation. Such epigenetic silencing, impairing EMT and wound closure, is recognized as a therapeutic target that is subject to specific in vivo gene editing. Wound healing represents an evolutionarily conserved, intricate, and dynamic process aimed at preserving life (54). A chronic wound may be viewed as a condition wherein the wound healing cascade has been derailed by one or more limitations in physiological repair processes (55). Underlying limitations in perfusion, causing ischemia, are commonly reported in chronic wounds (56). Studies in tumor biology have identified the hypoxia component of ischemia as a potent inducer of gene hypermethylation (57). DNA methylation is known to dynamically regulate critical biological processes directly implicated in cutaneous wound closure. Such processes include skin reepithelialization, dermal regeneration, and neoangiogenesis (42, 54, 58, 59).

In this work, scRNA-Seq analyses of human chronic WE tissue characterized its heterogenous cellular composition. Specifically, 2 distinct keratinocyte clusters were identified as Kera1 (*KRT14*^+^*KRT1*^+^) and Kera2 (*KRT19*^+^*KRT7*^+^). Additionally, enriched in *KRT8* and *KRT18*, evident in fetal mesenchymal cells (30), Kera2 is rich in genes relevant to cell plasticity. Genes such as KRT7 (60), KRT19 (61), KRT8 (61), and KRT18 (62), highly abundant in Kera2, are directly implicated in EMT. Human keratinocyte populations, *KRT14*^+^*KRT1*^+^ and *KRT19*^+^*KRT7*^+^, referred to as Kera1 and Kera2 in this work, have been evident in data reported by us as well as other laboratories (63–68). In those studies, Kera2 has been evident in human skin samples. Kera2 have been localized in the bottom/side of the epidermal rete ridges, hair follicles, and glandular and ductal cells (63–68). In this work, the Kera2 pool of keratinocytes has been found to be abundant in UW skin. However, in human chronic WE tissue, it is markedly depleted. Compared to Kera1, which accounts for almost all of the keratinocytes in human chronic WE tissue, Kera2 cells were enriched in genes related to cellular metabolism and glycolysis. Such genes are necessary to satisfy the metabolic demands of increased keratinocyte motility during EMT (69). Activation of glycolysis by EMT is required for cytoskeletal remodeling as well as increasing cell traction (70). Pyruvate kinase (PKM), elevated in Kera2, induces EMT via activation of β-catenin (71). Among other genes elevated in Kera2 are aldolase A (ALDOA) and phosphoglycerate kinase 1 (PGK1), encoding glycolytic enzymes that promote HIF1α-dependent EMT (72, 73). Thus, depletion of Kera2 in WE tissue is likely to contribute to wound chronicity.

Skin morphogenesis relies on EMT. EMT enables the acquisition of mesenchymal phenotype characterized by loss of cell adhesion and tight junction structures in epithelial cells (17). During this gain of epithelial plasticity, induction of prototypic epithelial markers is coupled with the loss of apical-basal polarity and increased cell motility caused by cytoskeleton reorganization. Turning down of intercellular adhesion facilitates keratinocyte migration in the epidermis proximal to wound margins, causing reepithelialization. EMT has been extensively studied in the context of tumor biology (19, 74). Such work has recognized direct regulation of EMT by epigenetic mechanisms (74). This work unveiled a set of target genes subject to epigenetic regulation in human chronic wounds. Analysis of the panel of such genes identified transcription factor and signaling inducers directly implicated in EMT. Beyond EMT, NOTCH and WNT signaling are active in several cellular compartments, which are relevant to the overall wound healing process (75–78). With reepithelialization, critically required for wound closure and thus for elimination of wound chronicity, the current work focuses on EMT, which is central to resurfacing the wound (16–18).

Ischemia is a common complicating factor underlying almost all clinically presented human chronic wounds (40). A major subset variable of ischemia is hypoxia (79). Hypoxia is known, primarily from works on tumor biology, to be a potent inducer of hypermethylation specifically targeting the EMT pathway. Hypoxia favors hypermethylation by impairing TET DNA demethylases because of limited availability of oxygen, an essential cofactor of TET enzymes (57). Tumor suppressor genes are specifically hypermethylated and silenced as a result (57). In human chronic WE tissue, the most affected of such genes was identified as *TP53*. CpG hypermethylation of the *TP53* promoter markedly attenuates its transcriptional activity (80). In this work, experimental murine studies lend credence to that notion. Studies testing the effect of ischemia revealed that 80% of the 14 CpGs analyzed in the *TP53* promoter remained methylated compared with 29% methylation under nonischemic conditions. The observation that the hypoxia component of wound ischemia hypermethylates the *TP53* promoter led to studies addressing the functional significance of this finding.

*TP53* gene expression is responsive to injury such that its response is dynamic during the course of healing (81–84). In the initial phase of healing where cell proliferation is necessary, TP53 is transiently downregulated (81–84). A strong rebound expression of TP53 in the latter phases of healing helps reestablish tissue homeostasis during the course of tissue remodeling (81). Functional wound closure, characterized by reestablishment of barrier function in the repaired skin, relies on epithelial plasticity (16). TP53 contributes to tissue plasticity (85). TP53-null mice exhibit severely blunted tissue plasticity and stunted tissue regeneration (86, 87). Thus, wound intervention aimed at rescuing TP53 function is of interest.

DNA methylation is reversible (88). Hypomethylating agents, such as 5-aza (available as VIDAZA or ONUREG) and its deoxyl-derivative decitabine (available as DACOGEN), have been approved by the FDA to treat patients with hematological malignancies (89, 90). This work demonstrated that topical administration of 5-aza caused demethylation of genes at experimental ischemic wound sites and achieved accelerated wound closure. Encouraging findings of this global pharmacological approach provided impetus to test the significance of rescuing *TP53* hypermethylation on wound closure. A TNT-based topical CRISPR/dCas9 system was thus employed wherein the goal was to generate chimeric versions of dCas9 fused with TET to achieve targeted *TP53* demethylation (91). This targeted DNA demethylation method is robust, and the increased gene expression can be maintained up to 80 days (92, 93). Specific *TP53* demethylation improved wound closure. This work provides evidence on the significance of nonviral topical gene editing as a productive technology platform to close complicated cutaneous wounds. In summary, this work recognizes that in human chronic wounds, WE tissue suffers from global gene silencing caused by hypermethylation. Global pharmacological demethylation achieved by topical administration of 5-aza was effective in restoring closure of ischemic wounds. The significance of hypermethylation of WE *TP53* was recognized by specific demethylation of this gene, which improved wound closure. This work, inspired by findings from patient-based tissue, identified a potentially novel paradigm in nonhealing wounds and provides direct cues to inform therapeutic strategies.

## Methods

Additional methods are provided in the Supplemental Methods.

*Reagents and antibodies*. Tissue culture materials were procured from Thermo Fisher Scientific. A list of antibodies used is provided in Supplemental Methods. Adam17 NM_009615.6 Lentifect and Negative Control Lentifect (LPP-Mm24237-Lv122-400) lentiviral particles were purchased from GeneCopoeia, Inc.; 5-azacytidne (A2385-1G) was purchased from Sigma-Aldrich, and SAM (B9003S) was purchased from New England Biolabs, Inc. DNMT1 (ID: 110914), DNMT3A (ID: HSS176224), and DNMT3B (ID: 111744) siRNAs were purchased from Thermo Fisher Scientific.

### MethylCap-Seq: library generation and data analysis

DNA (1 μg) isolated from chronic WE tissue (*n =* 3) or UW human skin (*n =* 3) was sonicated to generate 100–300 bp size fragments. This was followed by methylated DNA fragment enrichment using Diagenode AutoMethylCap kit following the manufacturer’s protocol. Illumina-compatible sequencing libraries were generated using the Kapa Hyper Prep kit. Single-end 50 bp sequencing was done using Illumina HiSeq 2500 to approximately 40 million clusters per sample. DNA methylation values from MethylCap-Seq data using PrEMeR-CG analysis (94) were computed for proximal promoters (1 kb up- and downstream of transcription start site or TSS). DMR analysis was performed using a mean vector test. A total of 4689 significantly different DMRs (FDR adjusted *P* < 0.05; 3661 hypermethylated and 1028 hypomethylated in chronic WE tissue) were selected for subsequent IPA analyses. A Circos plot was created using Circa (http://omgenomics.com/circa). Source code for the analysis has been uploaded at (https://github.com/bundschuhlab/PublicationScripts/tree/master/WoundEdgeHypermethylation).

### Total transcriptome: library generation and data analysis

Total transcriptome libraries were generated from chronic WE tissue (*n =* 3) or UW human skin (*n =* 3) using the Illumina TruSeq Stranded Total RNA Sample Prep kit with Ribo-Zero Gold according to the manufacturer’s instructions (Epicentre Biotechnologies). Briefly, 200 ng of total RNA was incubated with rRNA removal capture oligos followed by binding to magnetic beads for rRNA and mitochondrial RNA subtraction. The ribo-depleted RNA was used to generate cDNA libraries. All samples were indexed with Illumina adapters followed by PCR amplification. Paired-end 50 bp sequencing was performed with the Illumina HiSeq 2500 sequencer to approximately 40 million clusters per sample.

RNA-Seq FASTQ files were generated using Illumina CASAVA software. Sequencing results were first checked for quality control metrics using QuaCRS (95). Samples were then processed using a slightly modified version of the Cufflinks protocol (96). In brief, reads were first aligned to the human reference genome GRCh37.p13 using STAR version 2.4.0 (97), with both a first and second pass alignment. Cufflinks v2.2.1 (98) was used to assemble transcripts from the STAR alignments. Cuffquant was used to quantify isoform counts using a GTF file generated by Cuffcompare. A total of 57,825 annotated genes were detected using human Release 19 (GRCh37.p13). Out of these 57,825 genes, 20,327 were protein coding genes. Other significant classifications of genes were the following: Pseudogene, 13,920; lincRNA, 7109; antisense, 5273; miRNA, 3049; misc_RNA, 2033; snRNA, 1916; snoRNA, 1457; sense_intronic, 741; rRNA, 526; processed_transcript, 514; others, 960. Finally, the Cuffdiff utility was used to call DEGs, which were then compared and sorted using custom scripts written in Python. Additional processing of data was performed by using dChip software (Harvard University, Cambridge, Massachusetts).

#### Cell culture.

Immortalized human keratinocytes (HaCaT, provided by NE Fusenig of German Cancer Research Center, Heidelberg, Germany) or murine dermal keratinocytes (Kera-308, purchased from Cell Line Services) were grown in low-glucose DMEM (10% FBS and 1% antibiotic) (Life Technologies). The cells were maintained in a standard culture incubator with humidified air containing 5% CO_2_ at 37°C.

#### Bisulfite conversion and sequencing.

Bisulfite conversion of DNA from cultured cells or human tissue was done using Cells-to-CpG Bisulfite Conversion kit (Thermo Fisher Scientific) as per our previous report (42) or using EZ DNA Methylation-Gold kit (Zymo Research). The primers for the sequencing region of the human *TP53* promoter were used as previously reported by Kim et al. (99). For sequencing the murine *TP53* promoter, the following primers were used: forward, TGTATTTTTTTTTGTTGGGGAAT; reverse, CCCTAATAATTACTTTAAATTC. A CpG site methylated in more than 30% of the total clones studied was termed as methylated.

#### Patients.

WE tissue was collected from patients with chronic wounds at the Comprehensive Wound Center of the Ohio State University and Indiana University. A chronic wound as per standard definition is a wound that fails to progress to healing or respond to treatment over the normal expected healing timeframe (4 weeks) (40). WE biopsy samples were obtained from 15 patients with chronic wounds with open wounds for more than 4 weeks. The Declaration of Helsinki protocols were followed. The demographics of patients with location of wounds included in this study are in Supplemental Table 7.

#### ScRNA-Seq.

Single-cell suspensions were generated from UW human skin and chronic WE biopsies. The resulting cell suspension was utilized for scRNA-Seq using the 10x Genomics platform using Chromium Next GEM Single Cell 3′ GEM, Library & Gel Bead kit v3.1and sequenced on an Illumina NovaSeq 6000.

Cell Ranger Single Cell Software Suite (http://support.10xgenomics.com/) was used to process the raw reads and to generate the count matrix. Seurat package in R (v3.1.0) (100–102) was used to perform the downstream analysis as reported (103). The samples were checked for quality control and then were integrated together. After filtering, the number of the cells became 50,729 cells (UW human skin: 25,561 cells; chronic WE: 25,168 cells). Identification of cluster markers was performed using a Wilcoxon rank-sum test by comparing each cluster with the rest of the cells using log fold-change 0.2 and adjusted *P* value less than 0.05. Source code for the analysis has been uploaded (https://github.com/SinghLabICRME/KS-Debridement_Project/blob/main/scRNA-seq_transcriptomics.md). For comparing and contrasting the chronic wounds with respect to acute wounds, scRNA-Seq from human day 7 WE tissue and uninjured skin from 3 healthy donors were retrieved from NCBI’s Gene Expression Omnibus (GEO) database under accession number GSE137897 (41) (also see Supplemental Methods).

#### Spatial transcriptomic analysis.

A 20 μm thick section was taken on a Visium spatial gene expression slide followed by permeabilization for 12 minutes. cDNA libraries were synthesized using Visium spatial gene expression reagent kit as per the manufacturer’s recommendations (PN-100186, 100190; 10x Genomics) and sequenced using NovaSeq 6000 (Illumina). Generated reads were preprocessed using the standard Space Ranger (https://support.10xgenomics.com/spatial-gene-expression/software) pipeline to align the raw sequencing reads onto hg38 genome and obtain the respective h5-format and spatial image files for UW human skin. The resulting preprocessed files were loaded in Seurat (104) for further downstream data processing and analysis. Briefly, we loaded the.h5 and spatial image files using the “Load10X_Spatial” module in Seurat and created Seurat Object for data processing. Sample quality was assured by computing the mitochondrial content (MT-content <15% retained) and filtering out the poor-quality spots using the “subset” module of Seurat. We used the “SCTransform” method (105) available in Seurat that implements the regularized negative binomial regression for normalizing the 10x Genomics spatial data. The spatial feature plot function in Seurat was used to visualize the spatial location and expression profile of candidate genes (belongs to Kera2) in UW human skin. Source code for the analysis has been uploaded (https://github.com/SinghLabICRME/KS-Debridement_Project/blob/main/spatial_transcriptomics.md).

#### Crisper/Cas9-mediated targeted DNA methylation/demethylation.

pdCas9-DNMT3A-EGFP and pdCas9-DNMT3A-EGFP (ANV) was a gift from Vlatka Zoldoš (University of Zagreb, Zagreb, Croatia) (Addgene plasmids 71666 and 71685) (46). In this plasmid, the catalytic domain of human DNMT3A (amino acids P602-V912) was derived from the plasmid pcDNA3/Myc-DNMT3A (Addgene plasmid 35521) (106). An undesired BbsI restriction site was removed by site-directed mutagenesis without affecting the amino acid sequence. The DNMT3A active site motif ENV was mutated to ANV (E756A) (46). The sequences of the human *TP53* guide RNAs used were a) CCGGTTCATGCCGCCCATGC; b) CGCTATCTGAGCAGCGCTCA; c) CCCCGGACGATATTGAACAA; d) GAGCGCTGCTCAGATAGCGA; and e) CCCACGGAACCCGCGGAGCC. pCAG-scFvGCN4sfGFPTET1CD and pCAG-dCas9-5xPlat2AflD were gifts from Izuho Hatada (Gunma University, Maebashi, Japan) (Addgene plasmids 82561 and 82560) (48). The sequence of the murine *TP53* guide RNAs used were a) CGACCCTAGGCGCTTGGCTT b) ATCCTCCTCCGATTCCGAGC and c) TTCCTTCCCGCCTTCCCGCT. Guide RNAs were purchased from GenScript USA Inc. and Applied Biological Materials Inc.

#### Animal studies.

Commercially available C57BL/6 mice, obtained from Harlan Laboratories Inc. or The Jackson Laboratory, were used for the experiments. Adult (8–12-week-old mice, approximately 25 g in weight) were used for experiments. Mice were housed individually after wounding with a 12-hour light/12-hour dark cycle and temperature in the institutional animal facilities and allowed access to food and water ad libitum.

### TNT2.0.

In vivo TNT2.0 was performed as described previously with a modification in the chip design (51, 52, 107). Keratinocyte-specific delivery of guide RNAs through TNT2.0 in murine skin was tested using flow cytometry.

#### Data availability.

The MethylCap-Seq, total RNA-Seq, 10x Genomics sequencing, and Visium spatial transcriptomics raw data are deposited in NCBI’s GEO under accession number GSE176417. All other data generated or analyzed during this study are included in this published article (and its supplemental materials).

#### Statistics.

GraphPad Prism v8.0 was used for statistical analyses. Data were assumed to be normally distributed for all analyses conducted. Data for independent experiments are presented as mean ± SEM unless otherwise stated. A Student’s *t* test (2-tailed) was used to test the significant differences among 2 groups. A *P* value less than 0.05 was considered significant. In the case of statistical comparison between more than 2 groups, 1-way ANOVA was used and Tukey’s honestly significant difference (HSD) post hoc test was applied. A *P* value less than 0.05 was considered significant.

#### Study approval.

Murine experiments presented in this study were reviewed and approved by the IACUC of Indiana University (Indianapolis, Indiana) or of The Ohio State University. All human tissue-based experiments were reviewed and approved by the IRB of Indiana University or The Ohio State University. Patients provided their written informed consent. Additionally, under IRB-approved protocols, surgically discarded and deidentified samples were collected. This included the collection of wound tissue and UW skin obtained from individuals undergoing elective surgeries. In the cases of surgically discarded and deidentified samples, informed consent was not required from the individuals.

## Author contributions

KS and CKS conceived and designed the work and wrote the manuscript. CKS and SR designed the clinical study and supervised sample collection and clinical annotation, with important help from GMG, MPM, KS, EH, SM, SSMS, SG, and KEW. KS and CKS designed and supervised scRNA-Seq experiments with important contributions from YR, MK, ASA, SL, JW, and RS. Sectioning and library generation for Visium spatial transcriptomics was done by YR and data analysis was done by SL, RS, and KS. Library generation and data analysis for MethylCap-Seq and total RNA-Seq was done by JAK and LAW under the supervision of PY and RB. IHC analysis and data interpretation were done by KS, DKK, DP, ST, YR, SSV, SK, and SM. Animal experiments and data analyses were done by KS, YR, ST, PV, EH, DKK, SKM, MK, PRG, SSV, and SK. Murine EMT experiments were done by YR with contributions from KS, SM, and EH. In vitro and In vivo CRISPR/dCas9 experiments were conceived by KS and performed by YR, EH, SSV, and PV. KS, SR, and CKS wrote the manuscript. CKS and KS are the guarantors of this work and, as such, had full access to all the data in the study and take responsibility for the integrity of the data and the accuracy of the data analysis. All authors read or provided comments on the manuscript

## Supplementary Material

Supplemental data

## Version 1 07/12/2022

In-Press Preview

## Version 2 09/01/2022

Electronic publication
