# Supplementary material for: Genome-wide DNA hypermethylation opposes healing in patients with chronic wounds by impairing epithelial-mesenchymal transition
Source: J Clin Invest. 2022 Sep 1;132(17):e157279. doi: 10.1172/JCI157279 (PMC9433101; doi:10.1172/JCI157279)
Supplement: Supplemental data [file jci-132-157279-s169.pdf]

Supplemental Methods & Figures

Genome-Wide DNA Hypermethylation Opposes Healing in Chronic Wound Patients by Impairing Epithelial-to-Mesenchymal Transition

Kanhaiya Singh[1,2,§] , Yashika Rustagi[1], Ahmed S Abouhashem[1,3], Saba Tabasum[1,2], Priyanka Verma[1], Edward Hernandez[1], Durba Pal[2,4], Dolly K Khona[1,2], Sujit K Mohanty[1], Manishekhar Kumar[1], Rajneesh Srivastava[1], Poornachander R Guda[1], Sumit S Verma[1], Sanskruti Mahajan[1], Jackson A Killian[5], Logan A Walker[5], Subhadip Ghatak[1,2] , Shomita S. Mathew-Steiner[1,2], Kristen E Wanczyk[1], Sheng Liu[6], Jun Wan[6], Pearlly Yan[7], Ralf Bundschuh[5,8,9], Savita Khanna[1,2], Gayle M Gordillo[1], Michael P Murphy[1], Sashwati Roy[1,2] and Chandan K Sen[1,2,§]*

[1]Indiana University Health Comprehensive Wound Center, Indiana Center for Regenerative Medicine and Engineering, Department of Surgery, Indiana University School of Medicine, Indianapolis, IN

[2]Department of Surgery, The Ohio State University, OH

[3]Sharkia Clinical Research Department, Ministry of Health, Egypt

[4]Department of Biomedical Engineering, Indian Institute of Technology Ropar, Punjab, India

[5]Department of Physics, The Ohio State University, Columbus, OH

[6]Center for Computational Biology and Bioinformatics (CCBB), Indiana University School of Medicine, Indianapolis, IN

[7]Comprehensive Cancer Center, The Ohio State University, Columbus, OH

[8]Department of Chemistry & Biochemistry, [9]Division of Hematology, The Ohio State University, Columbus, OH

§Corresponding Authors:

Chandan K. Sen, PhD
975 W Walnut Street, Suite IB-454A
Indianapolis, IN 46202
Tel: 317-278-2736
Email: cksen@iu.edu

Kanhaiya Singh, PhD
975 W Walnut Street, Suite IB-444
Indianapolis, IN 46202
Tel.: +1 317-278-3411
E-mail: kanh@iu.edu

*Lead contact: Chandan K Sen, PhD, cksen@iu.edu

List of antibodies used in this study

Antibodies against DNMT1 (cat. no. ab19905), DNMT3A (cat. no. ab4897), DNMT3B (cat. no. ab2851), TP53 (cat. no. ab26), ADAM17 (cat. no. ab2051 and ab57484), NOTCH1 (cat. no. ab27526), activated NOTCH1 (cat. no. ab8925 and ab52301), TWIST1 (cat. no. ab50581), SMURF1 (cat. no. ab38866), P73 (cat. no. ab40658), SIRT1 (cat. no. ab32441), 5-hydroxymethylcytosine (cat. no. ab214728), Cytokeratin 19 (cat. no. ab15463) and HDAC1 (cat. no. ab19845) were purchased from Abcam, Cambridge, MA. Antibodies against Slug (cat. no. 9585T), Vimentin (cat. no. 5741T), N-cadherin (cat. no. 13116T), 5-methylcytosine (cat. no. 28692S), Zeb1 (cat. no. 3396S), and P63 (cat. no. 4892S) were purchased from Cell Signaling Technology, Inc. Danvers, MA. Antibodies against E-cadherin (cat. no. 13-1900) and Cytokeratin 7 (cat. no. MAI-06315) were purchased from Thermo Fisher Scientific, Waltham, MA. Antibody against HIF-1 alpha (cat. no. NB100) was purchased from Novus Biologicals, LLC, Centennial, CO. Antibody against F4/80 (cat. no. MCA497R) was purchased from Bio-Rad Laboratories, Inc. Hercules, CA. Antibody against Col1A2 (cat. no. sc-393573) was purchased from Santa Cruz Biotechnology, Inc. Dallas, TX.

Dot blot analysis

Dot blot analysis was performed to detect global DNA methylation levels in human UW skin and chronic WE. Genomic DNA was isolated using the QIAamp DNA Mini Kit (cat. no. 51306, Qiagen, Germantown, MD). A total of 500 ng of genomic DNA was denatured with 0.4M NaOH, 25mM EDTA at 95°C for 10 min and then neutralized by adding an equal volume of cold 4 M ammonium acetate (pH 7.0). The denatured DNA were then spotted on a nitrocellulose membrane. The spotted membrane was then washed with 2X SSC buffer and kept in UV transilluminator for auto crosslinking. The membrane was then blocked with 5% non-fat milk in 1X TBST for 1 h and incubated overnight with anti-5mC-anti-rabbit antibody (1:500 dilution) in 1X TBST at 4°C. The following day, the membrane was washed and incubated with a HRP-conjugated anti-rabbit secondary antibody (1:5000) for 2h at room temperature. The membrane was washed, and the images were acquired using HyGLO chemiluminescent detection reagent (Denville Scientific, Metuchen, NJ). 5mc intensity was detected by an Azure c600 Gel Imaging System (Azure Biosystems, Dublin, CA). The dot blot intensity was quantified by ImageJ software (NIH).

Monolayer scratch cell migration assay: HaCaT cells were seeded in 12 well plates at a density of $1.5x10^5$/mL in DMEM media supplemented with 10%FBS, 1% antibiotic and 4.5g of glucose. After specific pretreatment or transfection, a scratch was created with a 1000 µL pipette tip and cells were washed with 1xPBS twice. Three images were captured per well at different locations at 0 hour and 44-hour post-scratching using a light microscope. Wound width was calculated using ImageJ software.

RNA isolation and quantitative real-time PCR (RT-qPCR). RNA extraction was done from UW human skin or chronic WE tissue samples or cultured cells using the miRVana kit (Ambion™,

cat. no. AM1560). cDNA preparation was done from mRNA using qScript™ SuperMix (Quanta Biosciences, cat. no. 95048-100). RT-qPCR was done using SYBR green–based chemistry and primers specific for selected genes **(Sup Table S8).** Quantification of gene expression was done using the delta-delta Ct relative quantization method after normalization with 18S.

Single cell suspension: Generation of single cell suspension was done as previously reported(1). Briefly, the tissue was chopped into small pieces and subjected to enzymatic dissociation using whole skin dissociation kit (cat. no. 130-101-540) in MACS C Tubes (cat. no. 130-093-237) (Miltenyi Biotec Inc., San Diego, CA). The samples were incubated in a water bath at 37°C for approximately 3 hours. After incubation, the samples were diluted by adding 0.5 mL of cold cell culture medium in order to stop the enzymatic reaction. The C Tubes were then attached to gentleMACS™ Octo dissociator and skin dissociation program was performed. The cell suspension was subjected to a 70 μm pre-Separation Filter in order to remove the debris. RBC lysis was performed using RBC lysis buffer (cat. no. 420301, BioLegend, San Diego, CA).

Single cell RNA seq quality control (QC) and analysis:

QC parameters for data analyses was done as previously reported(1, 2). Cells expressing more than 15% mitochondrial genes were excluded. Cells with very high total counts (>40,000) and very low counts (<3,000) were excluded. Also, genes that were detected in fewer than 3 cells were excluded. Log normalization for genes values was performed using 10,000 as scaling factor. The top 2000 highly variable genes among the eight samples were chosen for performing canonical correlation analysis to integrate the samples and to identify cells with similar gene expression profiles. Principal component analysis (PCA) was performed and the first 21 principal components were chosen to reduce the data dimensions for the clustering. Cluster five and six were extracted from the whole object and PCA was re-performed using the top 2000 variable genes among cells from clusters five and six only. t-Distributed Stochastic Neighbor Embedding (tSNE) was chosen to reduce the data dimensions to two dimensions. The proportion of cell types positive for selected genes between skin and chronic WE were compared using a Chi-Square test(3). For comparison between myeloid cells between normal skin and chronic WE, Wilcoxon Rank Sum test was used to identify the differentially expressed genes. Genes with adjusted p value< 0.05, log2FC ±0.5 and expressed at least in 20% of cells in either chronic WE or normal skin were used for pathways enrichment analysis using Reactome database(4).

Acute wound analyses: Acute day 7 wounds and uninjured skin samples single cell RNA seq data (RPKM counts) were retrieved from the Gene Expression Omnibus (GEO) database under accession number GSE137897(5). For downstream analyses, only keratinocyte clusters were isolated. Next, RPKM values were log normalized with 10,000 as a scaling factor. The top 2,000 variable genes among keratinocytes were identified and were used to run PCA. Next, tSNE was performed using the first 20 components. To identify differentially expressed genes, gene expression values were compared between keratinocytes from acute wound skin and uninjured

skin samples. Only genes with expression level of at least 20% in either group were considered for the differential expression analysis. Pathways enrichment was performed using the Reactome database using genes with adjusted p value < 0.05 and log2FC ±0.5. The top significant pathways identified to be altered were visualized using Cytoscape software(6).

Pathway analysis: The Ingenuity Pathway Analysis database (QIAGEN Inc., Redwood City, CA, www.qiagen.com/ingenuity) predicts biological networks enriched in the dataset. Significantly differentially regulated genes/DMRs were subjected to functional analysis using IPA as reported by us previously(7-9). The enriched canonical pathways and upstream regulators was obtained using the comparison analysis function in IPA. The heat map or bar plots were used to visualize the upstream regulators and canonical pathways. Correlation of multiple analysis simultaneously (DMRs and DEGs) allowed identification of differentially regulated pathways and upstream regulators. Further, enrichment of Gene Ontology (http://geneontology.org/) biological processes and Reactome pathway analysis (http://www.reactome.org) was performed via ClueGo app v.2.5.3 (Cytoscape plug-in)(10). Cytoscape application was used for visualization of networks of pathways(6).

Wounding, digital planimetry and Laser Speckle Imaging (LSI): Mice were anesthetized using 1.5 percent isoflurane and carbogen (95% O_2 and 5% CO_2) mixture. The dorsum was shaved, cleaned, and sterilized. A bipedicle flap was developed on the back of the mice by making 30-mm-long full-thickness parallel incisions 10 mm apart as reported previously(11). Full-thickness excisional wounds were developed in the middle of each flap with a 3-mm disposable biopsy punch. In another experiment, monopedicle flap was created on the dorsal skin of the mice by making 30-mm-long full-thickness parallel incisions 10 mm apart. The bottom part of the skin was cut to create a free hanging flap. Flap edges were cauterized. A 0.5 mm silicon sheet was placed under the flap and then sutured to the adjacent skin with 5–0 ethicon silk suture. This Silicon sheet was not placed underneath the flap in murine TNT experiments. Digital images of the wounds were taken on the days as indicated. Perfusion was checked by laser speckle (PeriCam PSI HR System, PeriMed, Sweden) for validation of establishment of ischemia(12, 13). Wound area measurement was done by digital planimetry using Image-J software (NIH), as described previously(12, 13).

Tissue Nanotransfection 2.0: The hollow microneedle array was fabricated on a double side polished silicon wafer using a standard semiconductor process in a cleanroom environment as described(14, 15). The wafer was then transferred to another plasma etching system to perform a deep Si etching called the Bosch process, a common semiconductor process to achieve a vertical etching profile with a high aspect ratio until the hollow microneedles were connected to the reservoirs so that the cargo or the plasmid DNA fluid could freely flow from the reservoir to the hollow microchannel. When an electric pulse was applied between the TNT chip and the tissue, the negatively charged plasma DNA would travel from the reservoir to nearby target cells by electrophoresis and enter them by electroporation.

Flow cytometry analyses: Single-cell suspensions were generated from the murine skin samples using whole skin dissociation kit (cat. no. 130-101-540) in MACS C Tubes (cat. no. 130-093-237; Miltenyi Biotec Inc., San Diego, CA). The fluorescence and light-scattering properties (forward scatter and side scatter) of the cells were determined by using an BD Accuri™ C6 Plus Flow Cytometer (BD). Signals from cells labeled with conjugated fluorophores were detected(16). Data was analyzed using Accuri C6 Software or FlowJo software. Gates were set manually. Gating strategy for the fluorophores has been shown in respective figures.

Histology, Immunohistochemistry, and Immunocytochemistry (ICC): Histology of skin was performed from 10-µm-thick paraffin sections after staining with H&E. In order to have consistency in the tissue morphology for different antibodies, serial sections from same tissue blocks were obtained and processed for control and treated samples. Paraffin tissue sections were deparaffinized using xylene and rehydrated using decreasing gradient of ethanol. Antigen retrieval was done using citrate buffer, blocked with 10% NGS and incubated with primary antibodies (dilution 1:100 for DAB staining and 1:200 for fluorescence staining) followed by appropriate fluorescence conjugated secondary antibodies (Alexa 568-tagged α-rat, 1:200 dilution; Alexa 488-tagged α-488 rabbit, 1:200 dilution) or HRP conjugated antibodies. For DAB staining, Vectastain Elite ABC Kit (Vector Laboratories, Burlingame, CA) was used as reported(17, 18). Slides were counterstained with hematoxylin. Cells having brown-stained cytoplasm were regarded as positive. Similar staining time and procedure were adopted for all the tissue samples. Images were collected using a Zeiss Axio Scan Z1 and Zeiss LSM 880 microscope guided by Zen blue imaging software(12, 13). IHC images were analyzed using the IHC Profiler(19) algorithm and Image-J software (NIH).

Western Blot: Cells or homogenized tissues were harvested using cell lysis buffer (Cell Signaling Tech., MA #9803), sonicated on ice (3 pulses for 3 sec) and centrifuged (14000 g for 20 min at 4°C). The protein estimation in the clear lysates was done using bicinchoninic acid (BCA) assay. Proteins (25-30 µg) were separated on 4-12% Bis-Tris Gel/MOPS running buffer (NuPAGE, cat. no. NP0321BOX and NP0001) (45 mins at 200V) using NuPAGE electrophoresis system (Invitrogen). Proteins were transferred to polyvinylidene difluoride membranes (PVDF) (2.15 h at 30V). Membranes were blocked with 5% skim milk in Tris-buffered saline with 0.1% Tween 20 for 1 h at room temperature and incubated with the respective primary antibodies overnight at 4°C (dilution TP53- 1:500; ADAM17- 1:500; activated NOTCH1- 1:500). The following day the membranes were washed and incubated with the corresponding HRP-conjugated secondary antibody (1:2000) for 1h at room temperature. The membranes were washed and the images were acquired using HyGlo chemiluminescent detection reagent (cat. no. E-2500, Thomas Scientific, NJ) and Azure c600 Gel Imaging System by Azure Biosystems(20). Beta–actin and GAPDH were used as loading control. Image J (NIH) software was used for quantification of bands by densitometry analysis.

Histone acetylation and methylation enzyme-linked immunosorbent (ELISA) assay: Histones were extracted using the EpiQuick Total Histone Extraction Kit (EpiGentek). Briefly, 1×10^5 HaCat cells were seeded in 12 wells, treated with 80µM SAM at 80% confluency for 48 hours before collection using Cell Striper (Corning). After extraction, histone protein concentration was determined using BCA™ Protein Assay Kit (Pierce, Rockford, IL) and analyzed using the

EpiQuick Total Histone H3 Acetylation Detection Fast Kit (EpiGentek) according to the manufacturer's protocols. Briefly, histone proteins (200ng) were added to the strip wells for 2 hours at room temperature. The wells were then washed and incubated with the secondary Ab-containing solution for 1 hour on rocker. The wells were then developed with the enzyme reaction solution for 5 min before being quenched and measured for absorbance at 450 nm. Histone H3K4 methylation analysis was done using EpiQuik global histone H3K4 methylation assay kit (EpiGentek). Briefly, histone proteins (800ng) were added to the strip wells. Methylated histone H3K4 was detected with a high-affinity antibody, and the ratios and amounts of methylated histone H3K4 were determined with a horseradish peroxidase-conjugated secondary antibody using a color development system. Absorbance was measured at a wavelength of 450 nm.

RT^2 Profiler human wound healing PCR array: A sample of 1 μg of total RNA per array plate obtained from each group (vehicle control and SAM treated, 80μM SAM) was used for synthesis of cDNA using RT^2 First Strand kit (Qiagen). qPCR array was conducted using the RT^2 Profiler PCR Arrays (Qiagen); Eighty-four human genes related to wound healing (PAHs-121ZC) were assessed. A 1.5-fold cut-off was used to identify genes differentially expressed (up-regulated or down-regulated genes). Data analysis was performed using the RT^2 Profiler PCR array data analysis (https://geneglobe.qiagen.com/us/analyze).

References:

1. Rustagi Y, Abouhashem AS, Verma P, Verma SS, Hernandez E, Liu S, et al. Endothelial Phospholipase Cgamma2 Improves Outcomes of Diabetic Ischemic Limb Rescue Following VEGF Therapy. *Diabetes.* 2022;71(5):1149-65.
2. Abouhashem AS, Singh K, Azzazy HME, and Sen CK. Is Low Alveolar Type II Cell SOD3 in the Lungs of Elderly Linked to the Observed Severity of COVID-19? *Antioxid Redox Signal.* 2020;33(2):59-65.
3. Hughes TK, Wadsworth MH, 2nd, Gierahn TM, Do T, Weiss D, Andrade PR, et al. Second-Strand Synthesis-Based Massively Parallel scRNA-Seq Reveals Cellular States and Molecular Features of Human Inflammatory Skin Pathologies. *Immunity.* 2020;53(4):878-94 e7.
4. Gillespie M, Jassal B, Stephan R, Milacic M, Rothfels K, Senff-Ribeiro A, et al. The reactome pathway knowledgebase 2022. *Nucleic Acids Res.* 2022;50(D1):D687-D92.
5. Li D, Cheng S, Pei Y, Sommar P, Karner J, Herter EK, et al. Single-Cell Analysis Reveals Major Histocompatibility Complex IIExpressing Keratinocytes in Pressure Ulcers with Worse Healing Outcomes. *J Invest Dermatol.* 2022;142(3 Pt A):705-16.
6. Shannon P, Markiel A, Ozier O, Baliga NS, Wang JT, Ramage D, et al. Cytoscape: a software environment for integrated models of biomolecular interaction networks. *Genome Res.* 2003;13(11):2498-504.
7. Ghosh N, Das A, Biswas N, Gnyawali S, Singh K, Gorain M, et al. Urolithin A augments angiogenic pathways in skeletal muscle by bolstering NAD(+) and SIRT1. *Sci Rep.* 2020;10(1):20184.
8. Wisler JR, Singh K, McCarty AR, Abouhashem ASE, Christman JW, and Sen CK. Proteomic Pathway Analysis of Monocyte-Derived Exosomes during Surgical Sepsis Identifies Immunoregulatory Functions. *Surg Infect (Larchmt).* 2020;21(2):101-11.

9. Das A, M SEM, Gnyawali SC, Ghatak S, Singh K, Stewart R, et al. Skin Transcriptome of Middle-Aged Women Supplemented With Natural Herbo-mineral Shilajit Shows Induction of Microvascular and Extracellular Matrix Mechanisms. *J Am Coll Nutr.* 2019;38(6):526-36.
10. Bindea G, Mlecnik B, Hackl H, Charoentong P, Tosolini M, Kirilovsky A, et al. ClueGO: a Cytoscape plug-in to decipher functionally grouped gene ontology and pathway annotation networks. *Bioinformatics.* 2009;25(8):1091-3.
11. Biswas S, Roy S, Banerjee J, Hussain SR, Khanna S, Meenakshisundaram G, et al. Hypoxia inducible microRNA 210 attenuates keratinocyte proliferation and impairs closure in a murine model of ischemic wounds. *Proc Natl Acad Sci U S A.* 2010;107(15):6976-81.
12. Singh K, Pal D, Sinha M, Ghatak S, Gnyawali SC, Khanna S, et al. Epigenetic Modification of MicroRNA-200b Contributes to Diabetic Vasculopathy. *Mol Ther.* 2017;25(12):2689-704.
13. Singh K, Sinha M, Pal D, Tabasum S, Gnyawali SC, Khona D, et al. Cutaneous Epithelial to Mesenchymal Transition Activator ZEB1 Regulates Wound Angiogenesis and Closure in a Glycemic Status-Dependent Manner. *Diabetes.* 2019;68(11):2175-90.
14. Zhou X, Brown BA, Siegel AP, El Masry MS, Zeng X, Song W, et al. Exosome-Mediated Crosstalk between Keratinocytes and Macrophages in Cutaneous Wound Healing. *ACS Nano.* 2020;14(10):12732-48.
15. Xuan Y, Ghatak S, Clark A, Li Z, Khanna S, Pak D, et al. Fabrication and use of silicon hollow-needle arrays to achieve tissue nanotransfection in mouse tissue in vivo. *Nat Protoc.* 2021.
16. Sinha M, Sen CK, Singh K, Das A, Ghatak S, Rhea B, et al. Direct conversion of injury-site myeloid cells to fibroblast-like cells of granulation tissue. *Nat Commun.* 2018;9(1):936.
17. Singh K, Agrawal NK, Gupta SK, Mohan G, Chaturvedi S, and Singh K. Increased expression of endosomal members of toll-like receptor family abrogates wound healing in patients with type 2 diabetes mellitus. *Int Wound J.* 2016;13(5):927-35.
18. Singh K, Agrawal NK, Gupta SK, Mohan G, Chaturvedi S, and Singh K. Genetic and epigenetic alterations in Toll like receptor 2 and wound healing impairment in type 2 diabetes patients. *J Diabetes Complications.* 2015;29(2):222-9.
19. Varghese F, Bukhari AB, Malhotra R, and De A. IHC Profiler: an open source plugin for the quantitative evaluation and automated scoring of immunohistochemistry images of human tissue samples. *PLoS One.* 2014;9(5):e96801.
20. Gordillo GM, Biswas A, Singh K, Sen A, Guda PR, Miller C, et al. Mitochondria as Target for Tumor Management of Hemangioendothelioma. *Antioxid Redox Signal.* 2021;34(2):137-53.

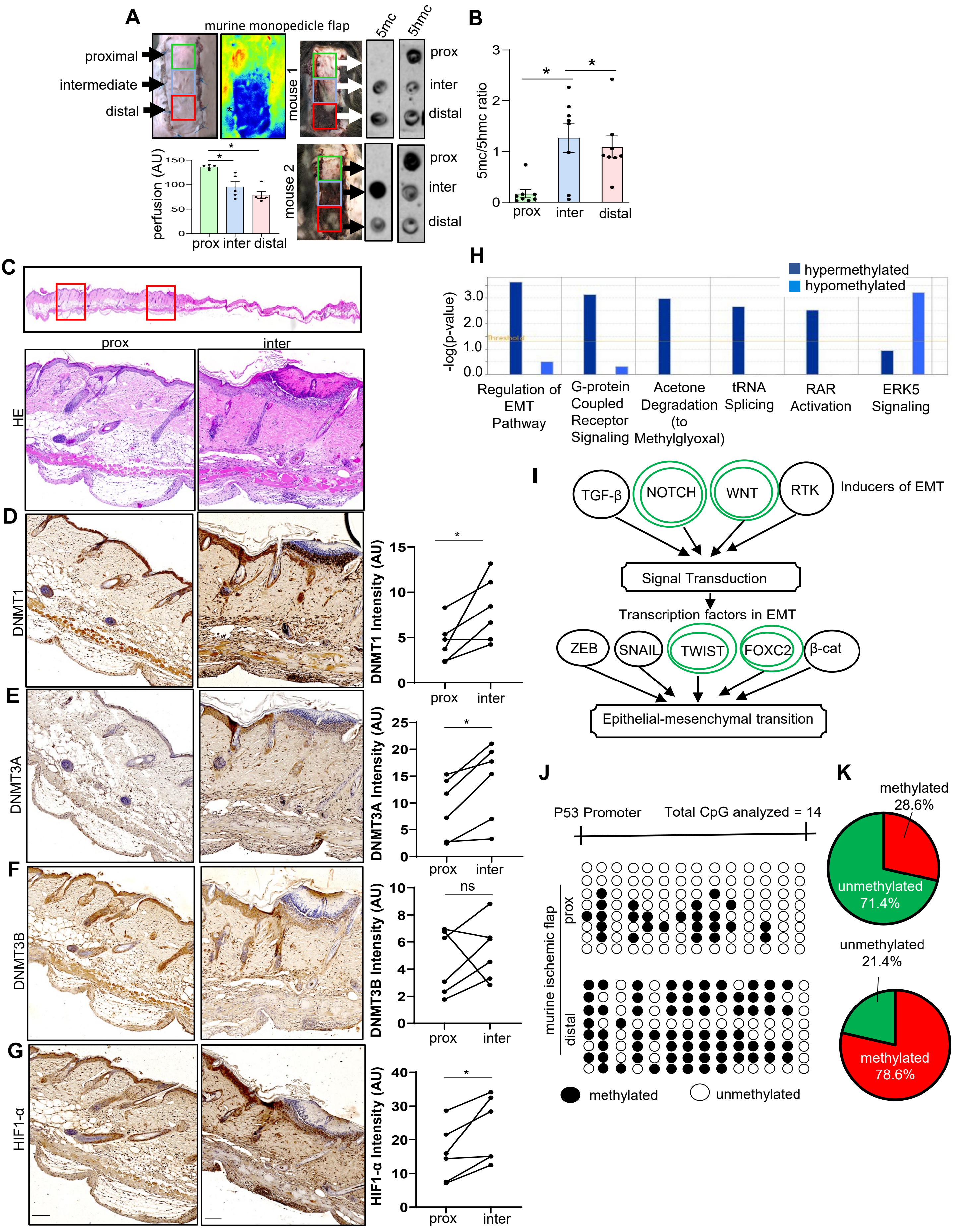
A
murine monopedicle flap
proximal
intermediate
distal
mouse 1
mouse 2
5mc
5hmc
prox
inter
distal
perfusion (AU)
prox inter distal
B
5mc/5hmc ratio
prox
inter
distal
C
prox
inter
HE
D
DNMT1
DNMT1 Intensity (AU)
E
DNMT3A
DNMT3A Intensity (AU)
F
DNMT3B
DNMT3B Intensity (AU)
ns
G
HIF1-α
HIF1-α Intensity (AU)
H
hypermethylated
hypomethylated
-log(p-value)
Threshold
Regulation of EMT Pathway
G-protein Coupled Receptor Signaling
Acetone Degradation (to Methylglyoxal)
tRNA Splicing
RAR Activation
ERK5 Signaling
I
TGF-β
NOTCH
WNT
RTK
Inducers of EMT
Signal Transduction
Transcription factors in EMT
ZEB
SNAIL
TWIST
FOXC2
β-cat
Epithelial-mesenchymal transition
J
P53 Promoter
Total CpG analyzed = 14
murine ischemic flap
prox
distal
methylated
unmethylated
K
methylated 28.6%
unmethylated 71.4%
unmethylated 21.4%
methylated 78.6%

Supplemental Figure 1. Ischemia induces DNA hypermethylation. (**A**) Monopedicle dorsal skin flap experiment showing samples collected from different flap regions. The extent of ischemia was categorically characterized by dividing the flap into three parts {proximal (green box), intermediate (blue box) and distal (red box) from cephalad attachment} in two different mice. The intermediate and distal regions of the flap were less perfused compared to proximal region (perfusion analysis, bottom panel) n=5; *$P<0.05$, One-way ANOVA. Dot blot analysis showing levels of 5-methylcytosine (5mc) and 5-hydroxymethylcytosine (5hmc) in different regions of monopedicle flap (proximal, intermediate and distal from cephalad attachment) in two different representative mice. (**B**) Intensity analysis of 5mc/5hmc ratio in abovementioned different regions of murine monopedicle dorsal skin flap (n=8; *$P<0.05$, One-way ANOVA). Data represented as the mean ± SEM. (**C**) Representative Hematoxylin and eosin staining and IHC analysis of DNMT1 (**D**), DNMT3A (**E**), DNMT3B (**F**) and HIF-1α (**G**) in paraffin sections from different regions of monopedicle flap (proximal and intermediate from cephalad attachment). Right panels represent the intensity analysis of the images. (Scale = 100μm; n = 6; *$P<0.05$, Paired t-test). (**H**) Canonical Pathway analysis by IPA software showing the top enriched network based on hyper and hypomethylated DMRs in chronic wound samples. (**I**) IPA canonical pathway (Regulation of the Epithelial-Mesenchymal Transition, EMT) with hypermethylated genes in human chronic WE highlighted in green. Some of the inducers of EMT (NOTCH, WNT), and transcription factors involved in EMT (TWIST and FOXC2) were hypermethylated in chronic WE. (**J**) Schematic diagram showing the region of murine P53 promoter (mm10_chr11:69,578,954-69,579,215) analyzed through bisulfite genomic sequencing of DNA isolated from different regions of murine monopedicle dorsal skin flap (proximal and distal from cephalad attachment). Diagrammatic representation of the promoter methylation status has been shown (methylated CpG, black; unmethylated CpG, white). (**K**) Venn diagram showing the distribution of methylated and unmethylated P53 promoter in different regions of murine monopedicle dorsal skin flap (proximal (**top**) and distal (**bottom**) from cephalad attachment).

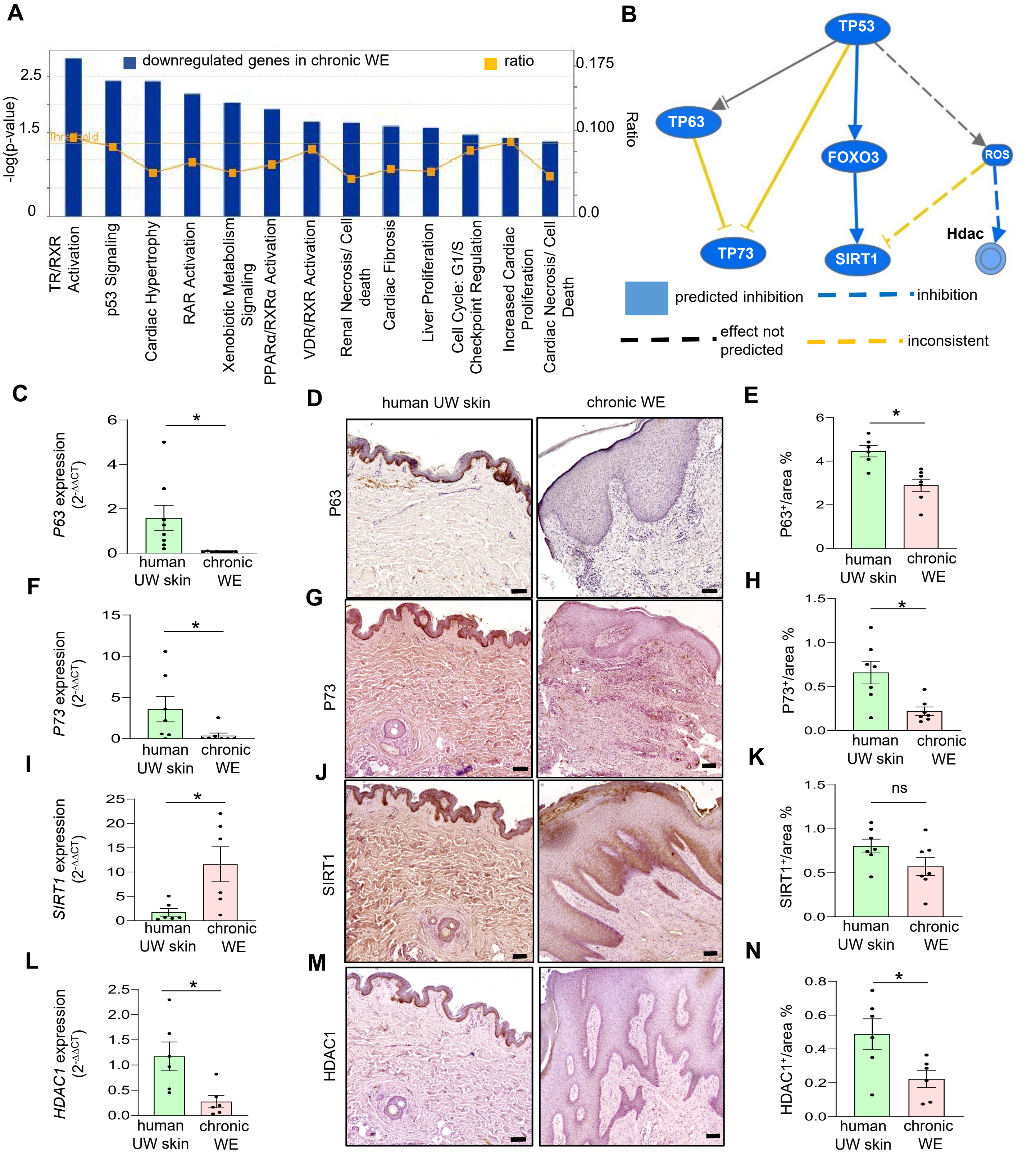
A
downregulated genes in chronic WE
ratio
-log(p-value)
Ratio
Threshold
TR/RXR Activation
p53 Signaling
Cardiac Hypertrophy
RAR Activation
Xenobiotic Metabolism Signaling
PPARα/RXRα Activation
VDR/RXR Activation
Renal Necrosis/ Cell death
Cardiac Fibrosis
Liver Proliferation
Cell Cycle: G1/S Checkpoint Regulation
Increased Cardiac Proliferation
Cardiac Necrosis/ Cell Death
B
TP53
TP63
FOXO3
ROS
TP73
SIRT1
Hdac
predicted inhibition
inhibition
effect not predicted
inconsistent
C
P63 expression (2-ΔΔCT)
human UW skin
chronic WE
D
human UW skin
chronic WE
P63
E
P63+/area %
F
P73 expression (2-ΔΔCT)
G
P73
H
P73+/area %
I
SIRT1 expression (2-ΔΔCT)
J
SIRT1
K
SIRT1+/area %
ns
L
HDAC1 expression (2-ΔΔCT)
M
HDAC1
N
HDAC1+/area %

Supplemental Figure 2. (**A**) Toxicological pathway analysis by IPA software showing the top enriched network based on downregulated genes in chronic WE sample. (**B**) IPA upstream regulator analysis of downregulated genes in chronic WE identified predicted regulators involved in TP53 signaling. (**C**) qRT-PCR, (**D**) IHC analysis of P63 and (**E**) intensity analysis of the images. (**F**) qRT-PCR, (**G**) IHC analysis of P73 and (**H**) intensity analysis of the images. (**I**) qRT-PCR, (**J**) IHC analysis of SIRT1 and (**K**) intensity analysis of the images. (**L**) qRT-PCR, (**M**) IHC analysis of HDAC1 and (**N**) intensity analysis of the images. (Scale = 100µm; n = 5-8; *$P<0.05$, Student's t-test). Data represented as the mean ± SEM.

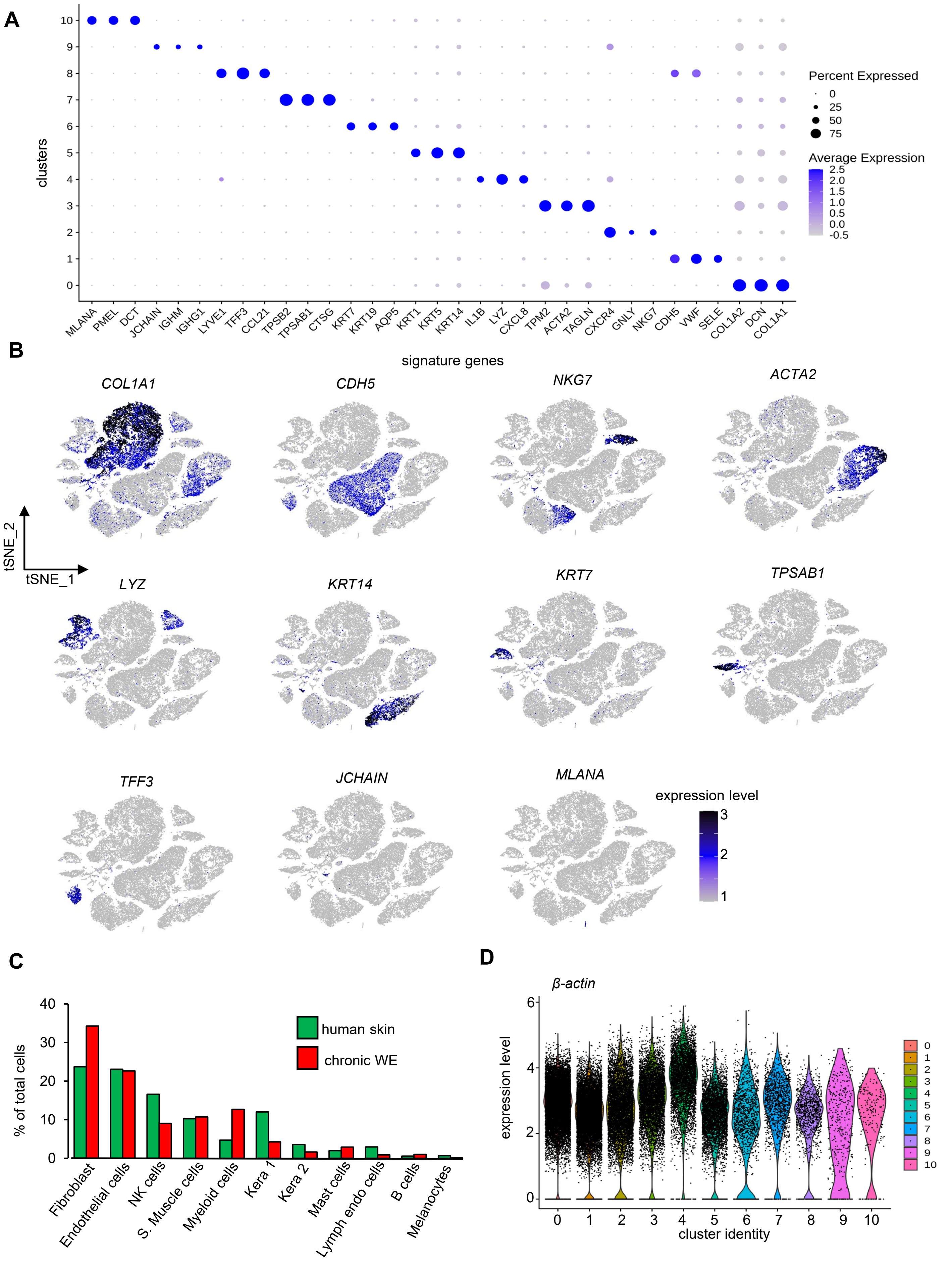
A
clusters
10
9
8
7
6
5
4
3
2
1
0
MLANA PMEL DCT JCHAIN IGHM IGHG1 LYVE1 TFF3 CCL21 TPSB2 TPSAB1 CTSG KRT7 KRT19 AQP5 KRT1 KRT5 KRT14 IL1B LYZ CXCL8 TPM2 ACTA2 TAGLN CXCR4 GNLY NKG7 CDH5 VWF SELE COL1A2 DCN COL1A1
signature genes
Percent Expressed
0
25
50
75
Average Expression
2.5
2.0
1.5
1.0
0.5
0.0
-0.5
B
COL1A1
CDH5
NKG7
ACTA2
tSNE_2
tSNE_1
LYZ
KRT14
KRT7
TPSAB1
TFF3
JCHAIN
MLANA
expression level
3
2
1
C
% of total cells
40
30
20
10
0
human skin
chronic WE
Fibroblast
Endothelial cells
NK cells
S. Muscle cells
Myeloid cells
Kera 1
Kera 2
Mast cells
Lymph endo cells
B cells
Melanocytes
D
β-actin
expression level
6
4
2
0
0 1 2 3 4 5 6 7 8 9 10
cluster identity

Supplemental Figure 3. (A) Dot plot of representative genes for identification of different eleven cellular clusters present in human skin: **cluster 0**, Fibroblast (*COL1A1*, *DCN*, *COL1A2*), **cluster 1**, Endothelial (*SELE*, *vWF*, *CDH5*), **cluster 2**, NK cells (*NKG7, GNLY, CXCR4*), **cluster 3**, Smooth Muscle Cells (*TAGLN, ACTA2, TPM2*), **cluster 4**, Myeloid cells (*LYZ, IL1B, CXCL8*), **cluster 5**, Keratinocyte 1 (*KRT14, KRT5, KRT1*), **cluster 6**, Keratinocyte 2 (*KRT7, KRT19, AQP5*), **cluster 7**, Mast Cells (*TPSAB1, CTSG, TPSB2*), **cluster 8**, Lymphatic endothelial cells (*LYVE1, TFF3, CCL21*), **cluster 9**, B cells (*IGHG1, IGHM, JCHAIN*) and **cluster 10**, Melanocytes (*DCT, PMEL, MLANA*). **(B)** tSNE plots showing the expression of one signature marker of each cluster as mentioned. (**C**) Bar graph showing percentage distribution of the cells in each cluster in human skin and in chronic WE. (**D**) Violin plot showing expression level of housekeeping gene *β-actin* in the identified 11 clusters.

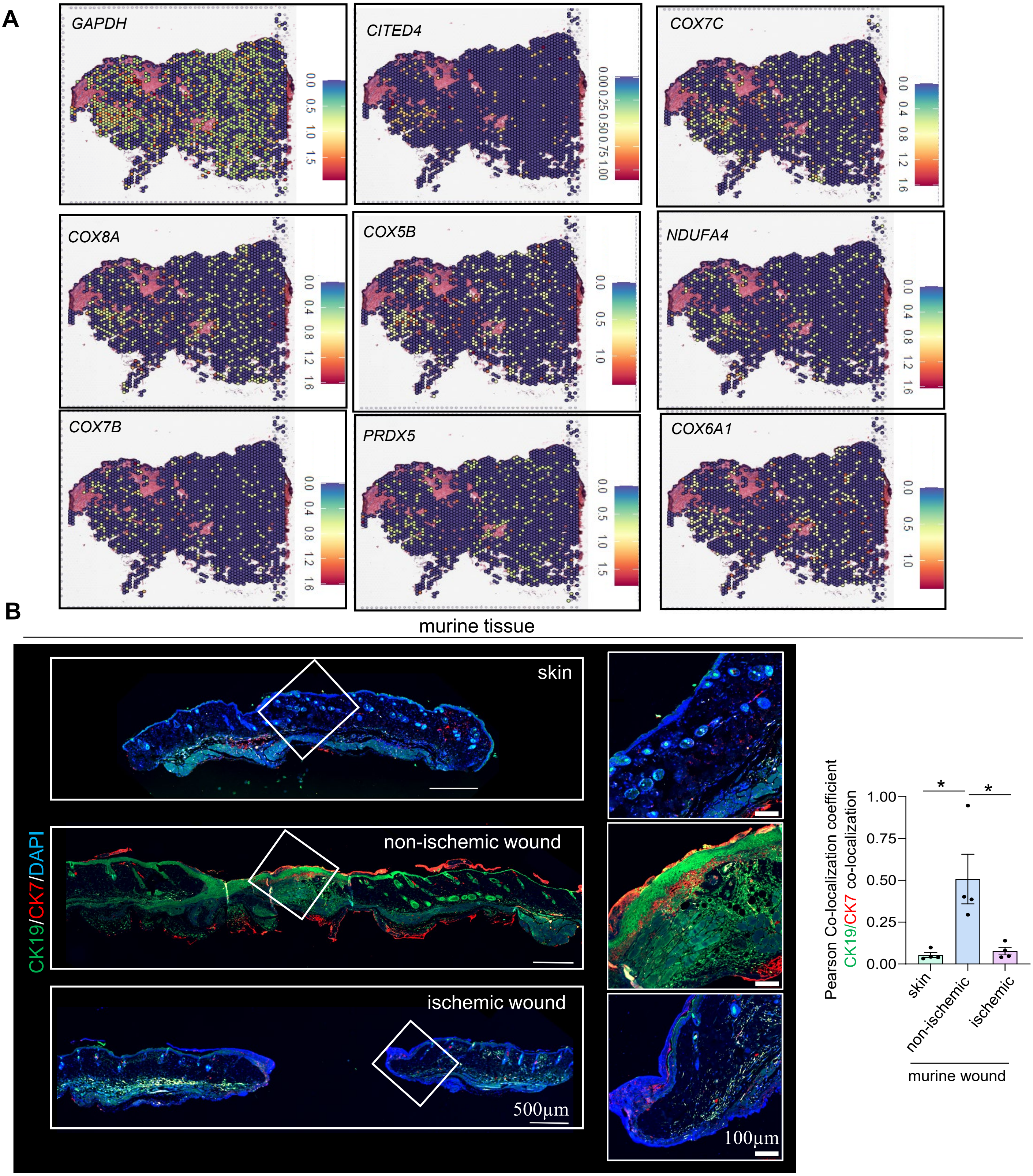

Supplemental Figure 4.(**A**) Spatial transcriptome profile (and localization) of top transcription factors, enzymes and metabolically active genes with increased expression levels in Kera 2 (**refer Figure 3 C-F**) of human skin shown using spatial feature plot function in Seurat. Scale bar for expression levels is provided, where the color towards red indicates high expression. (**B**) Representative IHC analyses for CK19–CK7 colocalization in ischemic and non-ischemic murine bipedicle wounds at day 3 post-wounding. Skin served as the baseline for colocalization analysis. Right panel represents the Pearson colocalization calculation. Data represented as the mean ± SEM. (n = 4, *P<0.05) (One-way ANOVA).

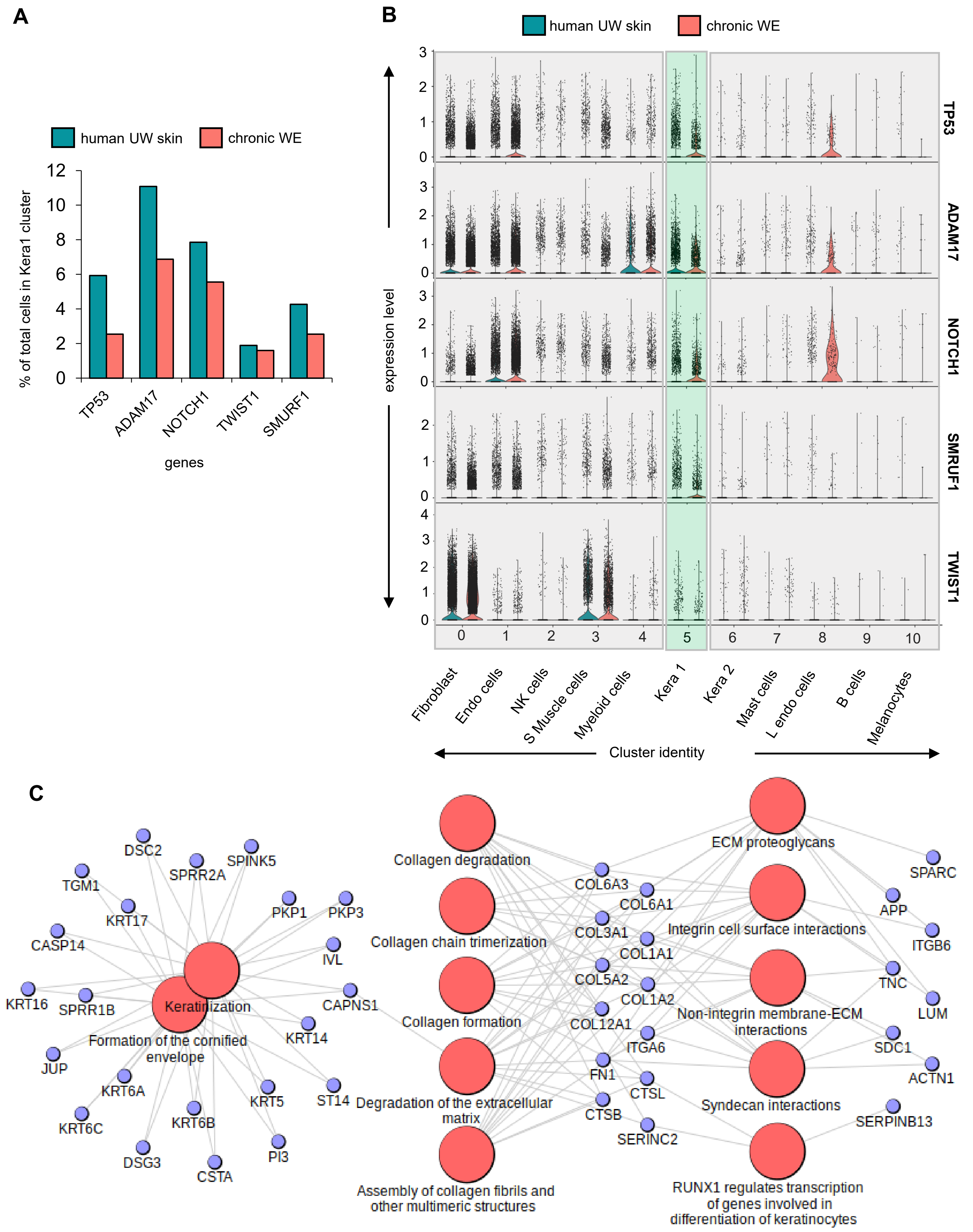
A
human UW skin
chronic WE
% of total cells in Kera1 cluster
TP53
ADAM17
NOTCH1
TWIST1
SMURF1
genes
B
human UW skin
chronic WE
expression level
TP53
ADAM17
NOTCH1
SMRUF1
TWIST1
Fibroblast
Endo cells
NK cells
S Muscle cells
Myeloid cells
Kera 1
Kera 2
Mast cells
L endo cells
B cells
Melanocytes
Cluster identity
C
Keratinization
Formation of the cornified envelope
DSC2
SPINK5
SPRR2A
TGM1
KRT17
PKP1
PKP3
CASP14
IVL
KRT16
SPRR1B
CAPNS1
KRT14
JUP
KRT6A
ST14
KRT5
KRT6C
KRT6B
PI3
DSG3
CSTA
Collagen degradation
Collagen chain trimerization
Collagen formation
Degradation of the extracellular matrix
Assembly of collagen fibrils and other multimeric structures
COL6A3
COL6A1
COL3A1
COL1A1
COL5A2
COL1A2
COL12A1
ITGA6
FN1
CTSL
CTSB
SERINC2
ECM proteoglycans
Integrin cell surface interactions
Non-integrin membrane-ECM interactions
Syndecan interactions
RUNX1 regulates transcription of genes involved in differentiation of keratinocytes
SPARC
APP
ITGB6
TNC
LUM
SDC1
ACTN1
SERPINB13

Supplemental Figure 5. Kera1 specific downregulation of identified EMT markers in chronic WE. (A) Bar graph representing total percentage of Kera1 cells expressing *TP53*, *ADAM17*, *NOTCH1*, *TWIST1* and *SMURF1* in Kera1 cluster obtained from human skin or chronic WE. (**B**) Violin plots comparing the expression level of *TP53*, *ADAM17*, *NOTCH1*, *SMURF1* and *TWIST1* in each cluster between human skin (left) and chronic WE (right). (**C**) Network of reactome enrichment of the upregulated pathways in Kera-1 cluster of chronic WE (adjusted p value < 0.001 and at least 8% of the pathway genes found in the DEG).

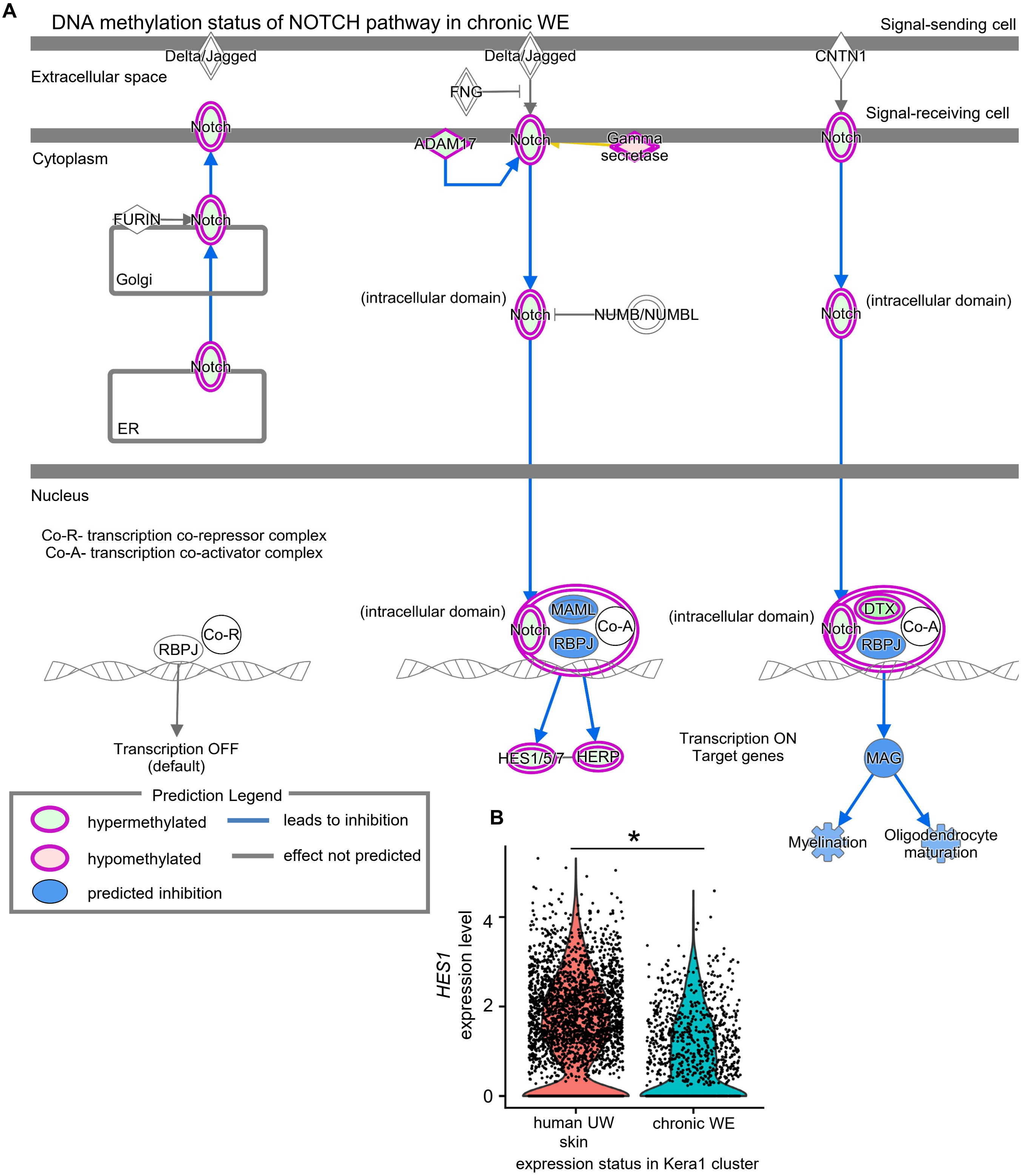

Supplemental Figure 6. **Methylation analysis of the NOTCH pathway in chronic WE**. (**A**) To investigate the methylation status of NOTCH signaling pathway in chronic WE, the list of molecules involved were retrieved from the EMBL-EBI database (taxon: Homo Sapiens). The intersection analysis between NOTCH molecules and differentially methylated genes in chronic WE revealed 28 DMRs significantly associated with chronic WE (24 hypermethylated, 4 hypomethylated). IPA analysis was done to visualize the methylation status of NOTCH signaling molecules. (**B**) Violin plot showing expression level of HES1 in Kera1 sub-cluster from human UW skin and chronic WE. * adjusted P value <0.00001, Wilcoxon Rank Sum test).

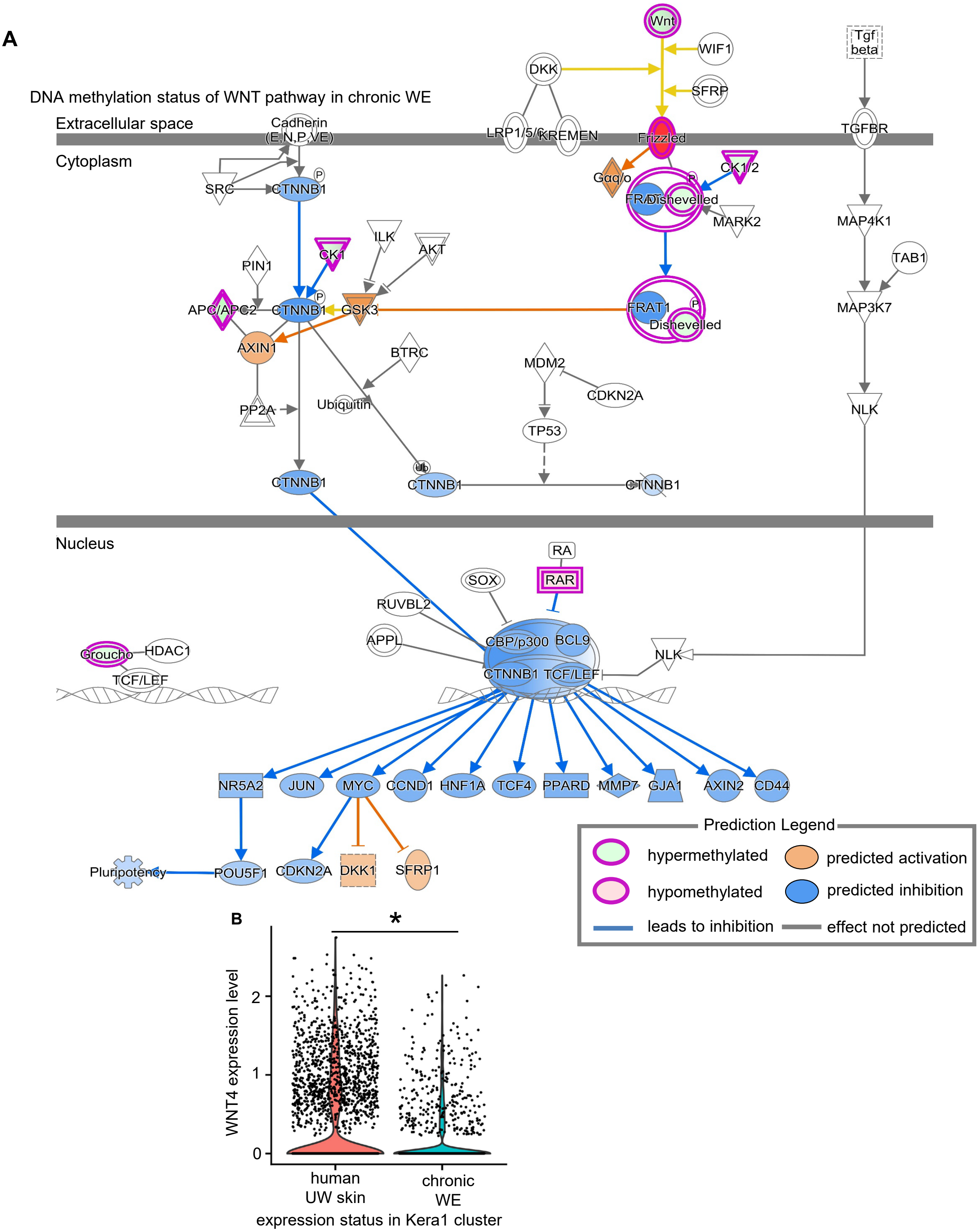

Supplemental Figure 7. **Methylation analysis of the WNT pathway in chronic WE**. (**A**) To investigate the methylation status of WNT signaling pathway in chronic WE, the list of molecules involved were retrieved from the EMBL-EBI database (taxon: Homo Sapiens). The intersection analysis between WNT molecules and differentially methylated genes in chronic WE revealed 44 DMRs significantly associated with chronic WE (36 hypermethylated, 8 hypomethylated). IPA analysis was done to visualize the methylation status of WNT signaling molecules. (**B**) Violin plot showing expression level of WNT4 in Kera1 sub-cluster from human UW skin and chronic WE. * adjusted P value <0.00001, Wilcoxon Rank Sum test.

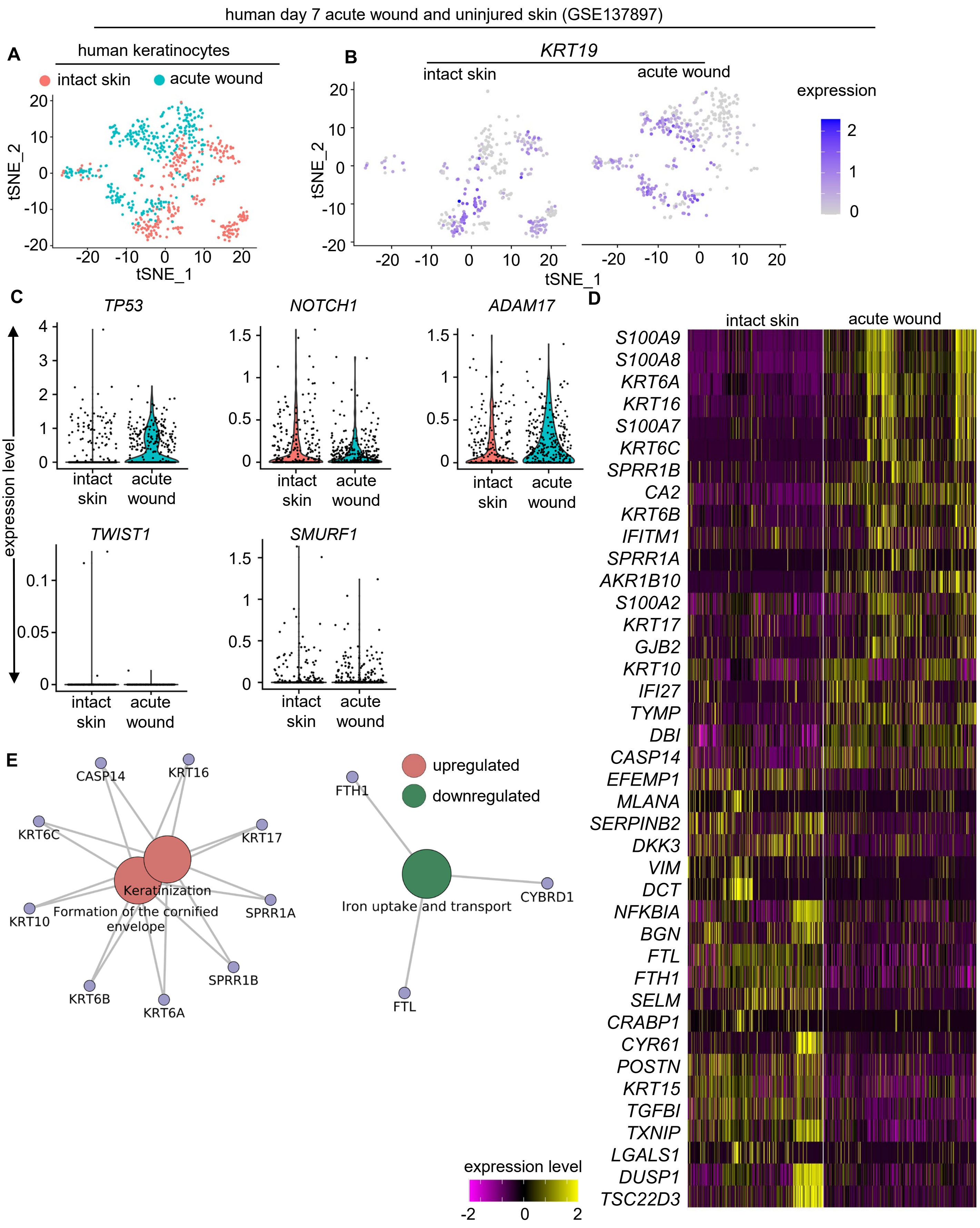

human day 7 acute wound and uninjured skin (GSE137897)
A
human keratinocytes
intact skin
acute wound
tSNE_2
tSNE_1
B
KRT19
intact skin
acute wound
expression
tSNE_2
tSNE_1
C
TP53
NOTCH1
ADAM17
TWIST1
SMURF1
expression level
intact skin
acute wound
D
intact skin
acute wound
S100A9
S100A8
KRT6A
KRT16
S100A7
KRT6C
SPRR1B
CA2
KRT6B
IFITM1
SPRR1A
AKR1B10
S100A2
KRT17
GJB2
KRT10
IFI27
TYMP
DBI
CASP14
EFEMP1
MLANA
SERPINB2
DKK3
VIM
DCT
NFKBIA
BGN
FTL
FTH1
SELM
CRABP1
CYR61
POSTN
KRT15
TGFBI
TXNIP
LGALS1
DUSP1
TSC22D3
expression level
-2
0
2
E
CASP14
KRT16
KRT6C
KRT17
Keratinization
Formation of the cornified envelope
KRT10
SPRR1A
SPRR1B
KRT6B
KRT6A
upregulated
downregulated
FTH1
Iron uptake and transport
CYBRD1
FTL

Supplemental Figure 8. Gene expression changes in keratinocytes from day 7 human acute wounds. **(A)** tSNE plot showing keratinocytes in the intact skin (314 cells) and in day 7 acute wound (355 cells) from (data from NCBI Gene Expression Omnibus (GSE137897). **(B)** Expression level of KRT19 (**Kera2 marker**) in keratinocytes from intact skin and day 7 acute wounds. **(C)** Violin plots showing expression level of genes of interest in intact skin and day 7 acute wound keratinocytes. These genes were not significantly different between intact skin and day 7 acute wound (based on fold change cut off). **(D)** Heatmap representing the top 20 upregulated and downregulated genes in keratinocytes from acute wound compared to intact skin. **(E)** Top upregulated and downregulated pathways in keratinocytes from acute wound compared to intact skin.

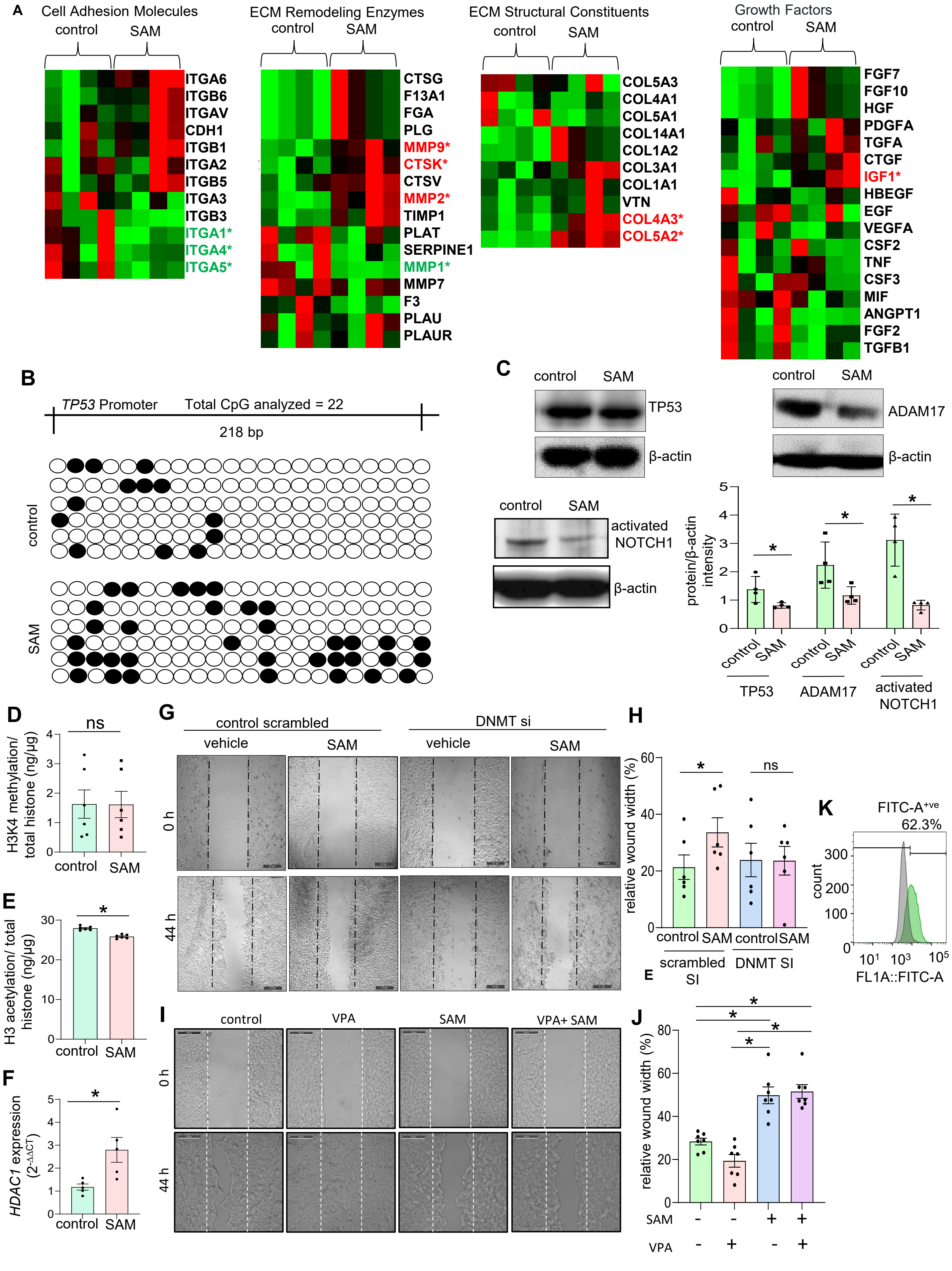

A
Cell Adhesion Molecules
control SAM
ITGA6
ITGB6
ITGAV
CDH1
ITGB1
ITGA2
ITGB5
ITGA3
ITGB3
ITGA1*
ITGA4*
ITGA5*
ECM Remodeling Enzymes
control SAM
CTSG
F13A1
FGA
PLG
MMP9*
CTSK*
CTSV
MMP2*
TIMP1
PLAT
SERPINE1
MMP1*
MMP7
F3
PLAU
PLAUR
ECM Structural Constituents
control SAM
COL5A3
COL4A1
COL5A1
COL14A1
COL1A2
COL3A1
COL1A1
VTN
COL4A3*
COL5A2*
Growth Factors
control SAM
FGF7
FGF10
HGF
PDGFA
TGFA
CTGF
IGF1*
HBEGF
EGF
VEGFA
CSF2
TNF
CSF3
MIF
ANGPT1
FGF2
TGFB1
B
TP53 Promoter Total CpG analyzed = 22
218 bp
control
SAM
C
control SAM
TP53
β-actin
control SAM
ADAM17
β-actin
control SAM
activated NOTCH1
β-actin
protein/β-actin intensity
control SAM control SAM control SAM
TP53 ADAM17 activated NOTCH1
D
ns
H3K4 methylation/ total histone (ng/µg)
control SAM
E
H3 acetylation/ total histone (ng/µg)
control SAM
F
HDAC1 expression (2-ΔΔCT)
control SAM
G
control scrambled DNMT si
vehicle SAM vehicle SAM
0 h
44 h
H
ns
relative wound width (%)
control SAM control SAM
scrambled SI DNMT SI
K
FITC-A+ve
62.3%
count
FL1A::FITC-A
I
control VPA SAM VPA+ SAM
0 h
44 h
J
relative wound width (%)
SAM - - + +
VPA - + - +

Supplemental Figure 9. SAM treatment induce DNA methylation in keratinocytes in a DNMT dependent fashion. (**A**) Heat map showing gene expression changes of wound healing related genes as catalogued by RT² Profiler™ PCR array (PAHS-121ZC) was done in HaCaT keratinocytes post 44h of scratch migration assay. Scratch migration assay was performed after 48 h of pretreatment with vehicle control or SAM (80 μM). Red, upregulated in SAM; Green, downregulated in SAM treated cells. Fold change cutoff = ±1.5. n = 4, *p< 0.05. (**B**) Schematic diagram showing the regions of *TP53* promoter (-1069bp to -821bp) analyzed through bisulfite genomic sequencing of DNA from HaCaT keratinocytes exposed to vehicle control or S-adenosylmethionine (SAM) (80 μM, 48 h). Diagrammatic representation of the promoter methylation status has been shown (methylated CpG, black; unmethylated CpG, white). The level of hypermethylated CpGs in TP53 promoter increased from basal 18% (vehicle control treated cells) to 45% in SAM treated keratinocytes. (**C**) Western blot analysis showing the expression of TP53 (left), ADAM17 (right) and activated NOTCH1 (bottom) in HaCaT keratinocytes exposed to vehicle control or S-adenosylmethionine (SAM) (80 μM, 48 h). β-actin was used as loading control. n =4; *P<0.05, Student's t-test. (**D**) Global histone H3K4 methylation and (**E**) total acetylated histone H3 enzyme-linked immunosorbent assay (ELISA) in HaCaT keratinocytes exposed to vehicle control or S-adenosylmethionine (SAM) (80 μM, 48 h). (n =6; *P<0.05, Student's t-test). **(F)** qRT-PCR analysis of HDAC1 expression in HaCaT keratinocytes exposed to vehicle control or SAM (80 μM). (n =5; *P<0.05, Student's t-test). **(G)** Representative images of scratch-wound migration assay of HaCaT keratinocytes . Scratch migration assay was performed after 48 h of pretreatment with vehicle control or SAM (80 μM) in presence of control scrambled or DNMT si and followed for 44 h after wounding. (**H**) Quantitation of relative wound with post-44 h of scratch wound was presented as percentage of wound area. n = 6, *p< 0.05 (One-way ANOVA). **(I)** Representative images of scratch-wound migration assay of HaCaT keratinocytes. Scratch migration assay was performed after 48 h of pretreatment with vehicle control or SAM (80 μM) followed for 44 h after wounding in presence of HDAC1 inhibitor valproic acid (VPA, 100 μM) or vehicle control. Scale bar = 500μm. (**J**) Quantitation of relative wound with post-44 h of scratch wound was presented as percentage of wound area. n = 6,7, *p< 0.05 (One-way ANOVA). (**K**) Flow cytometric detection of GFP expression in HaCaT keratinocytes as a indicative of the efficiency of dCas-9-DNMT3A-EGFP-ANV plasmid transfection.

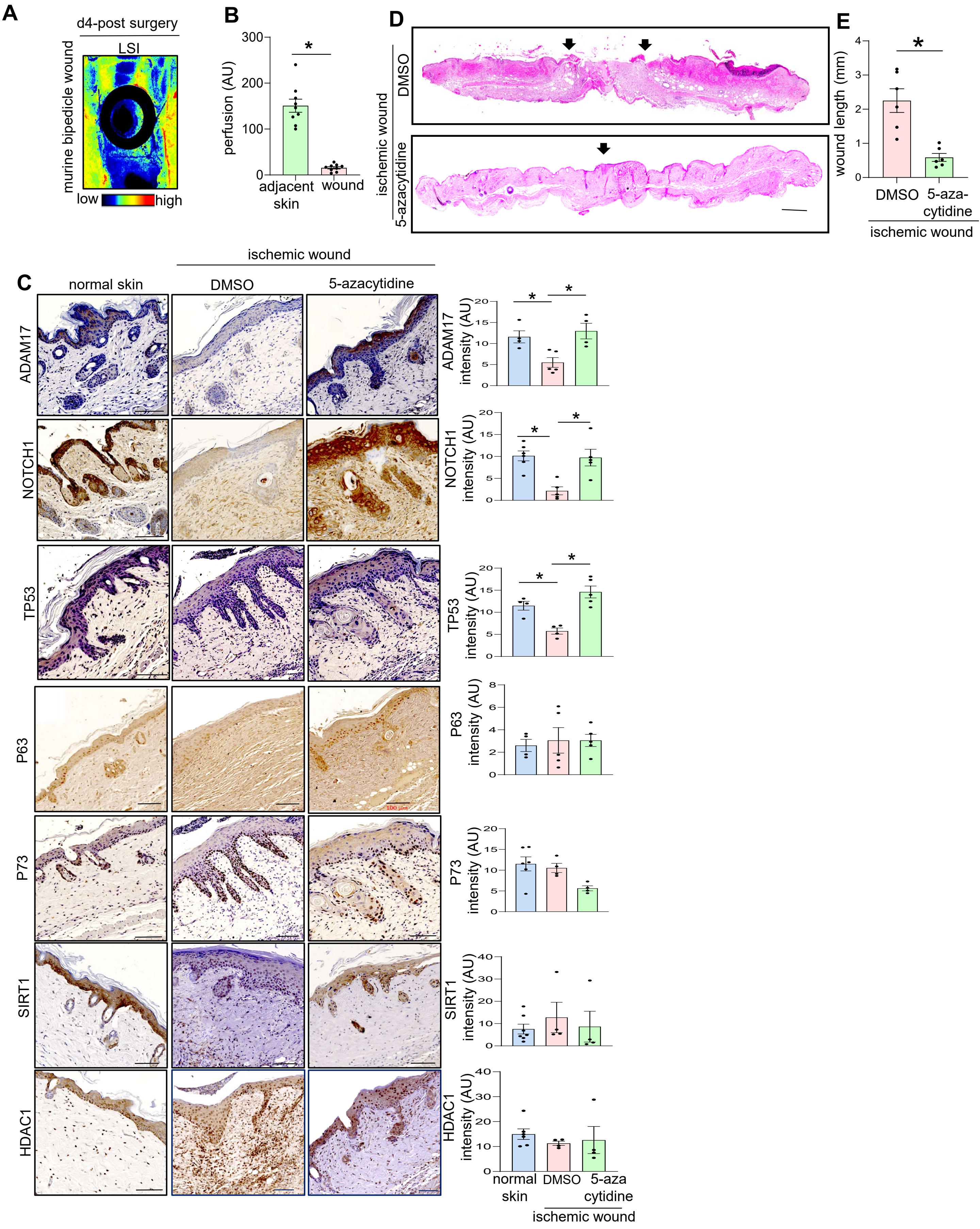

A
d4-post surgery
LSI
murine bipedicle wound
low
high
B
300
200
100
0
perfusion (AU)
*
adjacent skin
wound
D
DMSO
5-azacytidine
ischemic wound
E
4
3
2
1
0
wound length (mm)
*
DMSO
5-aza-cytidine
ischemic wound
C
ischemic wound
normal skin
DMSO
5-azacytidine
ADAM17
NOTCH1
TP53
P63
P73
SIRT1
HDAC1
ADAM17 intensity (AU)
NOTCH1 intensity (AU)
TP53 intensity (AU)
P63 intensity (AU)
P73 intensity (AU)
SIRT1 intensity (AU)
HDAC1 intensity (AU)
normal skin
DMSO
5-aza cytidine
ischemic wound

Supplemental Figure 10. Correction of hypermethylation improves ischemic wound closure by rescuing identified EMT regulators (**A**) Laser speck imaging (LSI) image of ischemic bipedicle wound at day 4 post-wounding. (**B**) Quantification of LSI images of bipedicle wound and the adjacent non-ischemic skin. (Scale = 0.5mm; n = 9; *P<0.05, Student's t-test). (**C**) Representative IHC analysis of TP53, ADAM17, NOTCH1, P63, P73, SIRT1 and HDAC1 in paraffin sections from murine skin and ischemic bipedicle wounds treated with either vehicle control or 5-azacytidine. Right panels represent the intensity analysis of the images. Scale bar, 100 µm. n = 4-7, *p< 0.05, One-way ANOVA. (**D**) Representative images of vehicle control or 5-azacytidine treated ischemic bipedicle wound biopsy tissue sections (10µm) of (day 10 post-wounding) stained using hematoxylin and eosin. (**E**) Bar graph represents the wound contraction in abovementioned mice groups. (n = 6,*p< 0.05, Student's t-test).

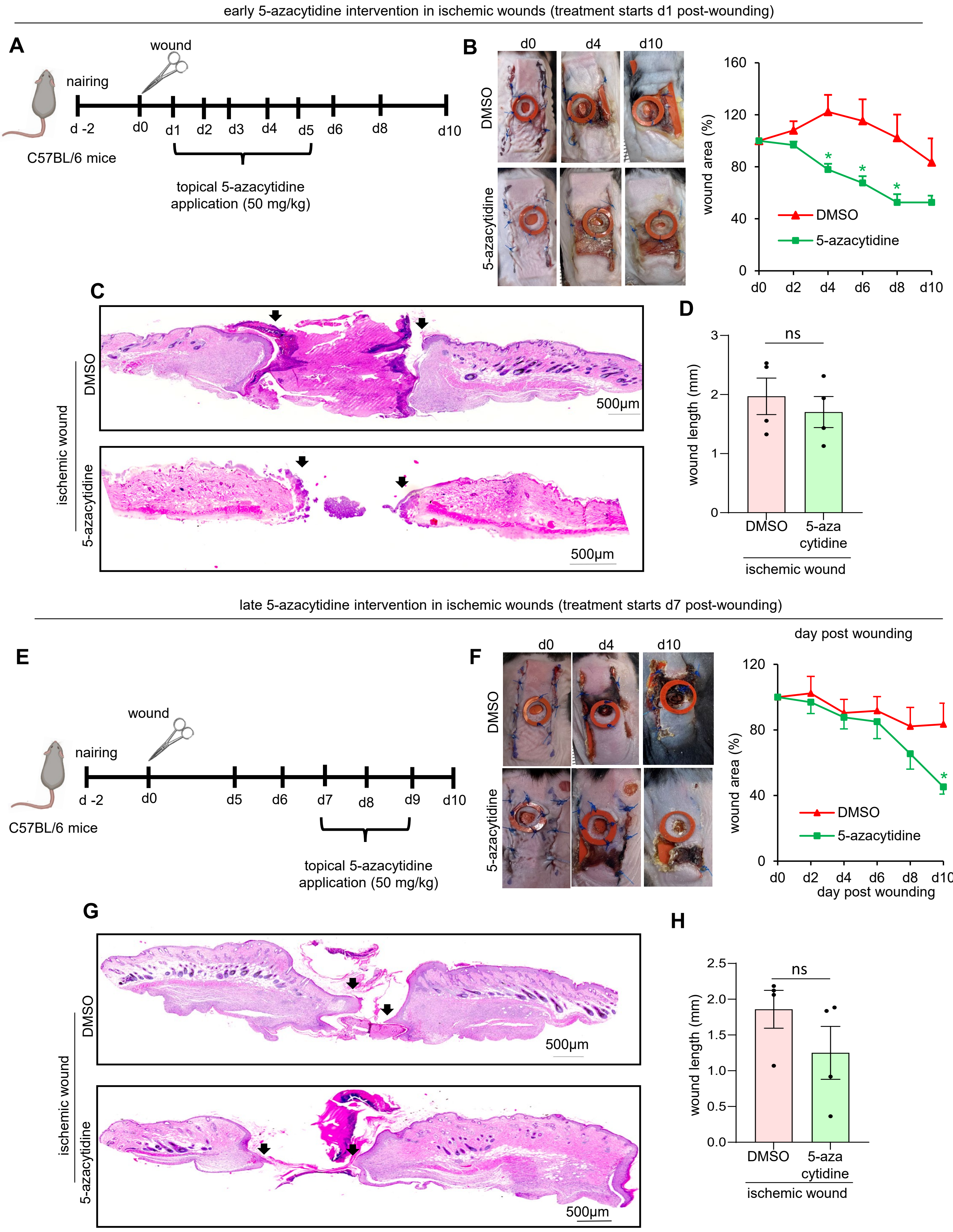
early 5-azacytidine intervention in ischemic wounds (treatment starts d1 post-wounding)
A
wound
nairing
d -2
d0
d1
d2
d3
d4
d5
d6
d8
d10
C57BL/6 mice
topical 5-azacytidine application (50 mg/kg)
B
d0
d4
d10
DMSO
5-azacytidine
wound area (%)
160
120
80
40
0
d0
d2
d4
d6
d8
d10
*
DMSO
5-azacytidine
C
DMSO
ischemic wound
5-azacytidine
500μm
500μm
D
ns
wound length (mm)
3
2
1
0
DMSO
5-aza cytidine
ischemic wound
late 5-azacytidine intervention in ischemic wounds (treatment starts d7 post-wounding)
E
wound
nairing
d -2
d0
d5
d6
d7
d8
d9
d10
C57BL/6 mice
topical 5-azacytidine application (50 mg/kg)
F
d0
d4
d10
DMSO
5-azacytidine
day post wounding
wound area (%)
120
80
40
0
d0
d2
d4
d6
d8
d10
*
DMSO
5-azacytidine
day post wounding
G
DMSO
ischemic wound
5-azacytidine
500μm
500μm
H
ns
wound length (mm)
2.5
2.0
1.5
1.0
0.5
0.0
DMSO
5-aza cytidine
ischemic wound

Supplemental Figure 11. **Effect of early (d1 post-wounding) and late (d7 post-wounding) 5-azacytidine treatment on ischemic wounds**. **(A)** Schematic diagram showing topical delivery of 5-azacytidine to the ischemic bipedicle wound created on dorsal skin of C57BL/6 mice starting from day 1 post-wounding. (**B**) Representative images (**left**) and wound closure analysis (**right**) at different days post-wounding in bipedicle ischemic wounds of mice treated with either vehicle control (DMSO) or 5-azacytidine by digital planimetry. Data was presented as percentage of wound area (**right**). n = 6; *$p < 0.05$ (Student's t-test). Data represented as the mean ± SEM. (**C**) Representative images of vehicle control or 5-azacytidine treated ischemic bipedicle wound biopsy tissue sections (10μm) of (day 10 post-wounding) stained using hematoxylin and eosin. (**D**) Bar graph represents the wound contraction in abovementioned mice groups. (n = 4). **(E)** Schematic diagram showing topical delivery of 5-azacytidine to the ischemic bipedicle wound created on dorsal skin of C57BL/6 mice starting from day 7 post-wounding. (**F**) Representative images (**left**) and wound closure analysis (**right**) at different days post-wounding in bipedicle ischemic wounds of mice treated with either vehicle control (DMSO) or 5-azacytidine by digital planimetry. Data was presented as percentage of wound area (**right**). n = 5,6; *$p < 0.05$ (Student's t-test). Data represented as the mean ± SEM. (**G**) Representative images of vehicle control or 5-azacytidine treated ischemic bipedicle wound biopsy tissue sections (10μm) of (day 10 post-wounding) stained using hematoxylin and eosin. (**H**) Bar graph represents the wound contraction in abovementioned mice groups. (n = 4).

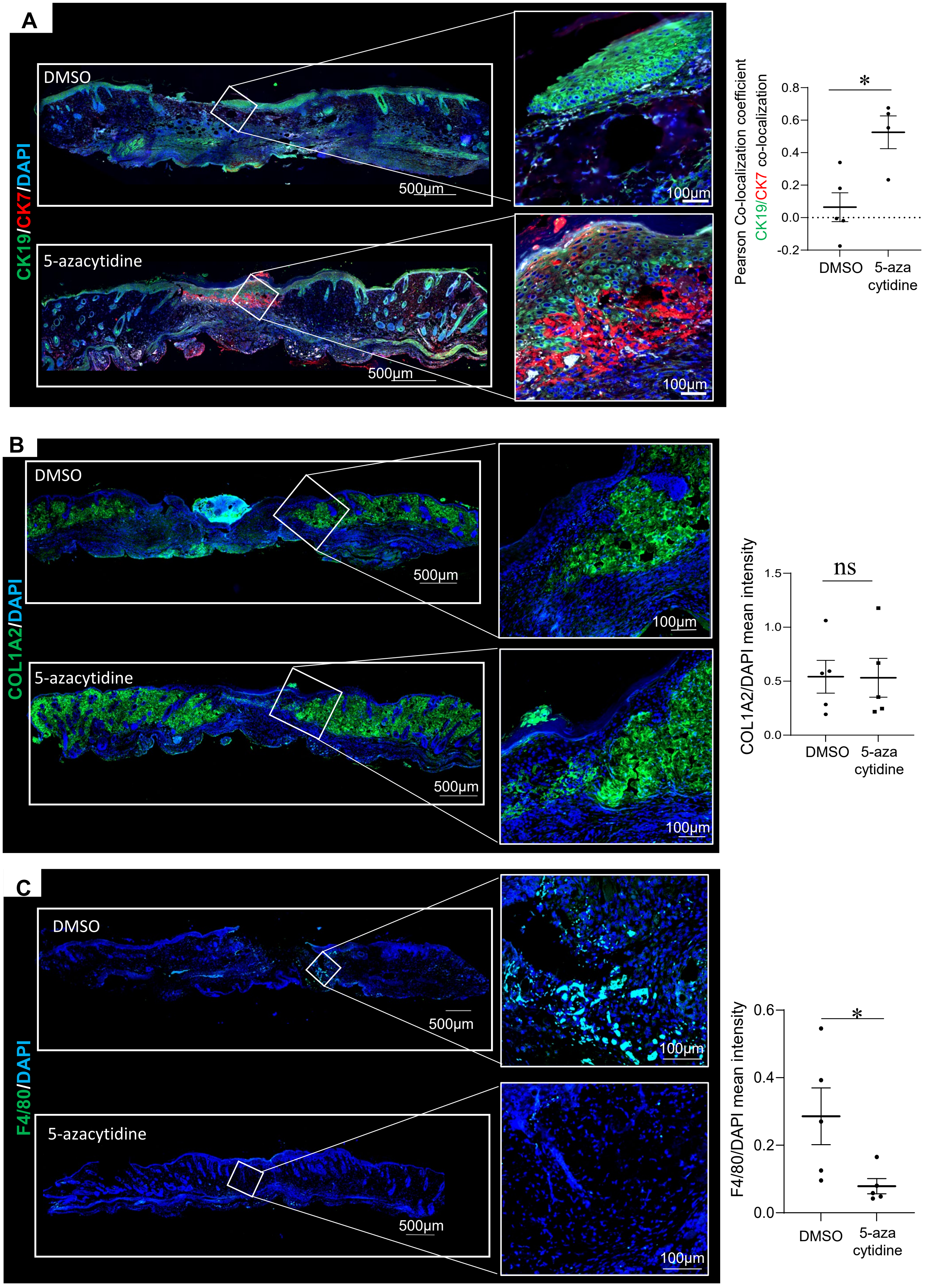
A
CK19/CK7/DAPI
DMSO
500μm
100μm
5-azacytidine
500μm
100μm
Pearson Co-localization coefficient
CK19/CK7 co-localization
*
0.8
0.6
0.4
0.2
0.0
-0.2
DMSO
5-aza cytidine
B
COL1A2/DAPI
DMSO
500μm
100μm
5-azacytidine
500μm
100μm
COL1A2/DAPI mean intensity
ns
1.5
1.0
0.5
0.0
DMSO
5-aza cytidine
C
F4/80/DAPI
DMSO
500μm
100μm
5-azacytidine
500μm
100μm
F4/80/DAPI mean intensity
*
0.6
0.4
0.2
0.0
DMSO
5-aza cytidine

Supplemental Figure 12. **Effect of 5-azacytidne mediated change on EMT program on different cellular compartments in murine wound-edge**. (**A**) Representative IHC images of d10 wound-edge for CK19–CK7 colocalization in vehicle control or 5-azacytidine treated (treatment started day 5 post-wounding) ischemic bipedicle wound biopsy tissue sections (10µm). Right panel represents the Pearson colocalization calculation. Data represented as the mean ± SEM. (n = 5,4,*p< 0.05, Student's t-test). (**B**) Representative IHC images of d10 wound-edge for COL1A2 intensity in vehicle control or 5-azacytidine treated (treatment started day 5 post-wounding) ischemic bipedicle wound biopsy tissue sections (10µm). Right panel represents the mean intensity analysis of the IHC images (n=5). (**C**) Representative IHC images of d10 wound-edge for F4/80 intensity in vehicle control or 5-azacytidine treated (treatment started day 5 post-wounding) ischemic bipedicle wound biopsy tissue sections (10µm). Right panel represents the mean intensity analysis of the IHC images. (*P<0.05, Student's t-test, n=5).

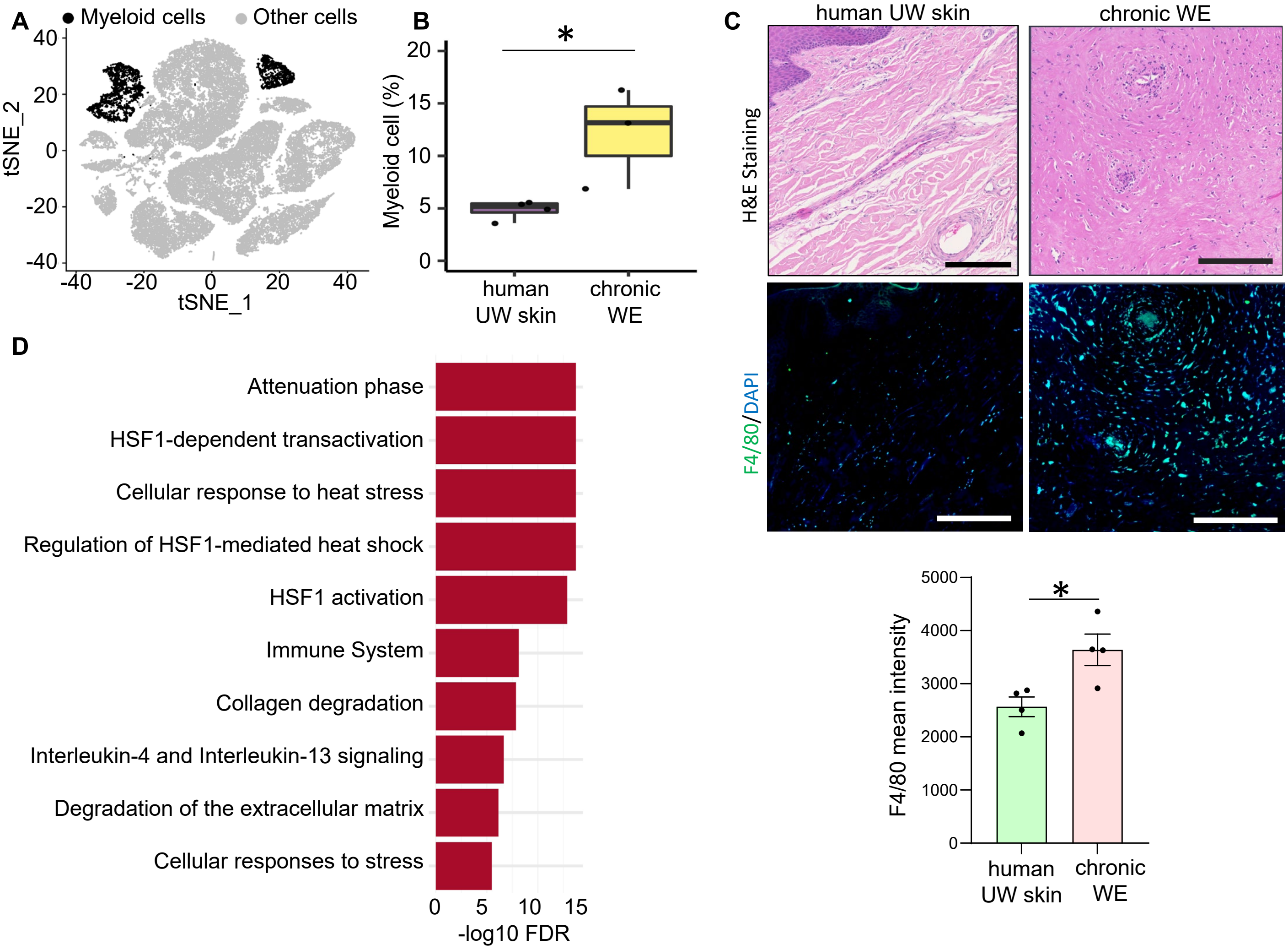

Supplemental Figure 13. More abundance of myeloid cells in chronic WE. (A) tSNE plot showing of myeloid cells (black dots) and other cells (grey dots) (Please also see **Figure 3A)**. Markers of myeloid cells has been defined as *LYZ*^{hi}*IL1B*^{hi}*CXCL8*^{hi} in **Supplemental Figures 3A, B**. **(B)** Box plot showing percentage of myeloid cells relative to other cells in each sample of human unwounded (UW) skin and in chronic wound-edge (WE) (*P<0.05, Student's t-test, n=4,3). (**C**) Representative H&E (top) and IHC analysis of F4/80 in paraffin sections of human UW skin and chronic WE. Bottom panel represent the mean intensity analysis of the IHC images. (Scale = 200μm; *P<0.05, Student's t-test, n=4). **(D)** Top 10 upregulated pathways in myeloid cells of chronic WE compared to human UW skin based on gene ontology analyses.

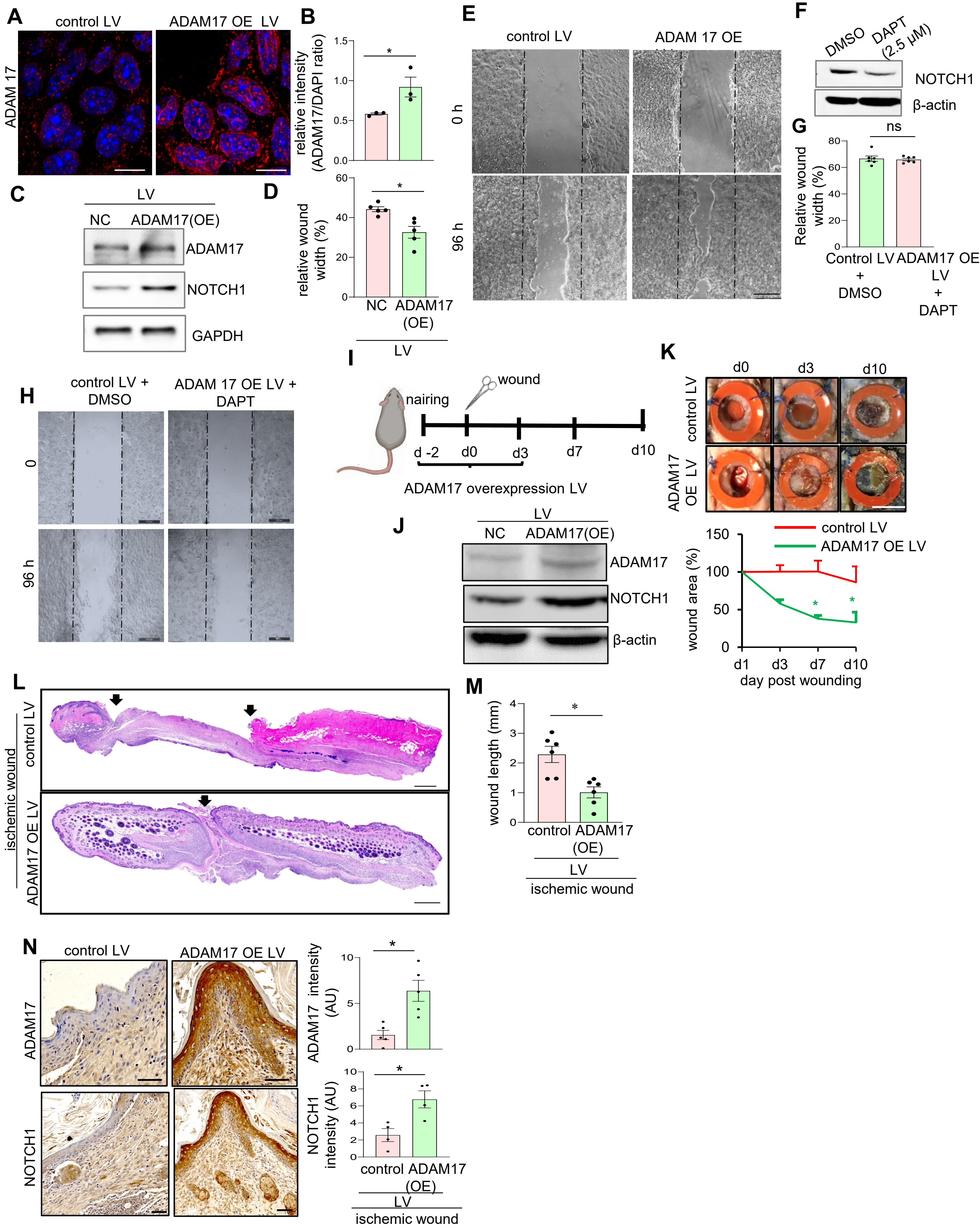

A
control LV
ADAM17 OE LV
ADAM 17
B
relative intensity (ADAM17/DAPI ratio)
C
LV
NC
ADAM17(OE)
ADAM17
NOTCH1
GAPDH
D
relative wound width (%)
NC
ADAM17 (OE)
LV
E
control LV
ADAM 17 OE
0 h
96 h
F
DMSO
DAPT (2.5 μM)
NOTCH1
β-actin
G
ns
Relative wound width (%)
Control LV + DMSO
ADAM17 OE LV + DAPT
H
control LV + DMSO
ADAM 17 OE LV + DAPT
0
96 h
I
nairing
wound
d -2
d0
d3
d7
d10
ADAM17 overexpression LV
J
LV
NC
ADAM17(OE)
ADAM17
NOTCH1
β-actin
K
d0
d3
d10
control LV
ADAM17 OE LV
control LV
ADAM17 OE LV
wound area (%)
d1
d3
d7
d10
day post wounding
L
ischemic wound
control LV
ADAM17 OE LV
M
wound length (mm)
control
ADAM17 (OE)
LV
ischemic wound
N
control LV
ADAM17 OE LV
ADAM17
NOTCH1
ADAM17 intensity (AU)
NOTCH1 intensity (AU)
control
ADAM17 (OE)
LV
ischemic wound

Supplemental Figure 14. Overexpression of ADAM17 was able to promote ischemic wound closure via enabling NOTCH1. Immunofluorescence analysis of ADAM17 in Kera-308 cells exposed to negative Control Lentifect™ (control LV) or ADAM17 NM_009615.6 Lentifect™ (ADAM17 OE LV) particles and (**B**) their intensity analysis. DAPI intensity was used for normalization. (Scale = 20µm; *P<0.001, Student's t-test). (**C**) Western blot analysis showing the lentivirus (LV) mediated overexpression of ADAM17 protein also increased the protein expression of NOTCH1. GAPDH was used as loading control. (**D**) Quantitation and (**E**) representative images of scratch-wound migration assay of Kera-308 cells. Scratch wound assay was performed after 48 h of transduction with (Control LV) or ADAM17 OE LV and followed for 96 h after wounding. (Scale = 200µm; n =5; *P<0.05, Student's t-test). (**F**) Western blot analysis showing the addition of DAPT (2.5 µM) mediated inhibition of NOTCH1 expression. B-actin was used as loading control. (**G**) Quantitation and (**H**) representative images of scratch-wound healing assay of Kera-308 cells. Scratch wound migration assay was performed after 48 h of transduction with Control LV or ADAM17 OE LV in presence or absence of NOTCH1 inhibitor DAPT and followed for 96 h after wounding. Data was presented as relative wound width. (n = 6, *p< 0.05) (Student's t-test). (**I**) Schematic diagram showing subcutaneous delivery of control LV or ADAM17 OE LV to the ischemic bipedicle wound created on dorsal skin of C57BL/6 mice. (**J**) Western blot analysis showing the lentivirus (LV) mediated overexpression of ADAM17 protein *in vivo* also increased the protein expression of NOTCH1. β-actin was used as loading control. (**K**) Wound closure was monitored at different days post-wounding in bipedicle ischemic wounds of mice transduced with either control LV or ADAM17 OE LV by digital planimetry (**top**). Data was presented as percentage of wound area (**bottom**). n = 5, 6, *P< 0.05 (Student's t-test). (**L**) Representative images of control LV or ADAM17 OE LV transduced ischemic wound biopsy tissue sections (10µm) of (day 10 post-wounding) stained using hematoxylin and eosin. Scale bar, 500 µm. (**M**) Bar graph represents the wound contraction in abovementioned mice groups. n = 6. *P, 0.05 (Student t-test). (**N**) Representative IHC analysis of ADAM17 (**top**) and NOTCH1 (**bottom**) in paraffin sections from ischemic bipedicle wounds transduced with either control LV or ADAM17 OE LV. Right panels represent the intensity analysis of the images. Scale bar, 50 µm. n = 4, 5, *p< 0.05. Data represented as the mean ± SEM.

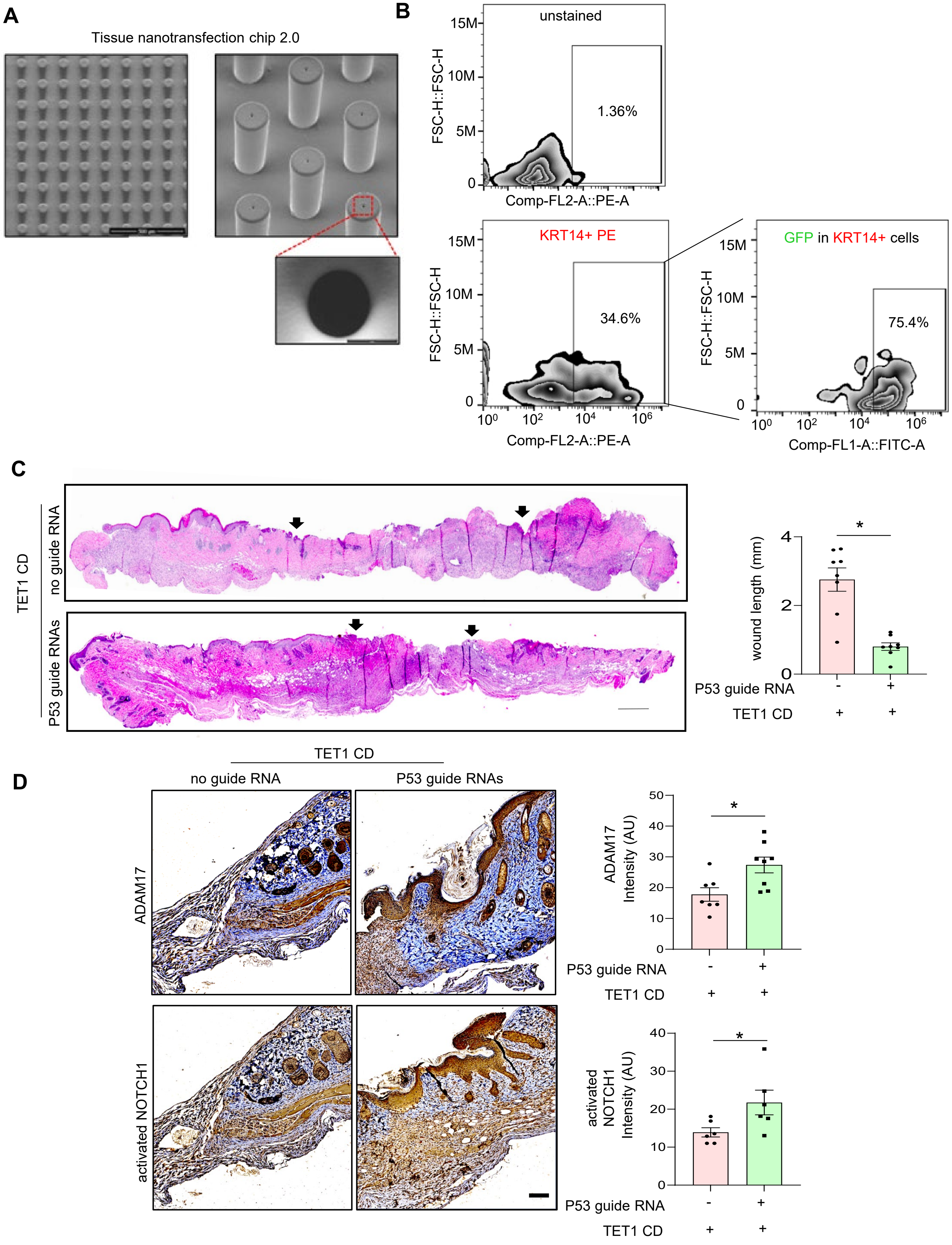
A
Tissue nanotransfection chip 2.0
B
unstained
15M
10M
5M
0
FSC-H::FSC-H
1.36%
Comp-FL2-A::PE-A
KRT14+ PE
34.6%
10^0 10^2 10^4 10^6
GFP in KRT14+ cells
75.4%
Comp-FL1-A::FITC-A
C
TET1 CD
no guide RNA
P53 guide RNAs
wound length (mm)
*
P53 guide RNA - +
TET1 CD + +
D
TET1 CD
no guide RNA
P53 guide RNAs
ADAM17
activated NOTCH1
ADAM17 Intensity (AU)
activated NOTCH1 Intensity (AU)
P53 guide RNA - +
TET1 CD + +

Supplemental Figure 15. TNT2.0 based demethylation of TP53 promoter. (**A**) Representative scanning electron microscopic images of tissue nanotransfection (TNT) chip 2.0. This modified TNT silicon chip ($TNT_{2.0}$) has a longer needle height of 170 µm and a pore diameter of 4 µm. Figure is reprinted (adapted) with permission from Zhou *et al.*, ACS Nano 2020, 14, 10, 12732–12748. Copyright (2020) American Chemical Society. (**B**) Flow cytometric validation of keratinocyte specific delivery of KRT-14 promoter driven P53 gRNA targets through TNT. A total of 70.9 ± 3.1 % $KRT14^{+}$ cells were also expressing GFP (n = 6 different skin sites from 3 animals). (**C**) (**Left**) Representative hematoxylin and eosin images bipedicle wound biopsy (day 10 post-wounding) tissue sections (10µm) of mice nanotransfected with TET1CD and peptide repeat in presence or absence of KRT14 promoter driven gRNA targets. (**Right**) Bar graph represents the wound contraction in abovementioned mice groups. n= 8, *P< 0.05 (Student's t-test), Scale bar, 500 µm. (**D**) Representative IHC analysis of ADAM17 (**top**) and activated NOTCH1 (**bottom**) in paraffin sections from murine ischemic bipedicle wounds nanotransfected with TET1CD and peptide repeat in presence or absence of KRT14 promoter driven P53 gRNA targets. For TET1CD-ADAM17 antibody staining, serial section from the same tissue block as in Figure 6K was used. Right panel represent the intensity analysis of the images. (Scale = 100µm; n = 6-7; *P<0.05, Student's t-test).

Table S1: List of genes associated with regulation of epithelial to mesenchymal transition pathway hypermethylated in chronic WE compared to human skin

Symbol	Entrez Gene Name	Location	Type(s)	Entrez Gene ID for Human
ADAM17	ADAM metallopeptidase domain 17	Plasma Membrane	peptidase	6868
DVL1	dishevelled segment polarity protein 1	Cytoplasm	other	1855
FGFR1	fibroblast growth factor receptor 1	Plasma Membrane	kinase	2260
FOXC2	forkhead box C2	Nucleus	transcription regulator	2303
HIF1A	hypoxia inducible factor 1 alpha subunit	Nucleus	transcription regulator	3091
KLB	klotho beta	Plasma Membrane	enzyme	152831
KRAS	KRAS proto-oncogene, GTPase	Cytoplasm	enzyme	3845
LOX	lysyl oxidase	Extracellular Space	enzyme	4015
MAP2K3	mitogen-activated protein kinase kinase 3	Cytoplasm	kinase	5606
MAP2K5	mitogen-activated protein kinase kinase 5	Cytoplasm	kinase	5607
MET	MET proto-oncogene, receptor tyrosine kinase	Plasma Membrane	kinase	4233
mir-155	microRNA 155	Cytoplasm	microRNA	406947
NFKB2	nuclear factor kappa B subunit 2	Nucleus	transcription regulator	4791
NOTCH4	notch 4	Plasma Membrane	transcription regulator	4855
PIK3R2	phosphoinositide-3-kinase regulatory subunit 2	Cytoplasm	kinase	5296
RRAS	RAS related	Cytoplasm	enzyme	6237
RRAS2	RAS related 2	Plasma Membrane	enzyme	22800
SMAD4	SMAD family member 4	Nucleus	transcription regulator	4089
SMURF1	SMAD specific E3 ubiquitin protein ligase 1	Cytoplasm	enzyme	57154
TCF4	transcription factor 4	Nucleus	transcription regulator	6925
TWIST1	twist family bHLH transcription factor 1	Nucleus	transcription regulator	7291
TWIST2	twist family bHLH transcription factor 2	Nucleus	transcription regulator	117581
WNT2	Wnt family member 2	Extracellular Space	cytokine	7472
WNT16	Wnt family member 16	Extracellular Space	other	51384
WNT8B	Wnt family member 8B	Extracellular Space	other	7479
WNT9A	Wnt family member 9A	Extracellular Space	other	7483

Table S2: List of significant upstream regulators predicted through IPA based on the hypomethylated or hypomethylated DMRs in chronic WE.

Upstream Regulator	Molecule Type	p-value of overlap
TP53	transcription regulator	0.0000144
EGF	growth factor	0.0000244
URI1	transcription regulator	0.0000651
ESR1	ligand-dependent nuclear receptor	0.0000734
BRCA1	transcription regulator	0.000124
miR-22-3p (miRNAs w/seed AGCUGCC)	mature microRNA	0.000145
ADAM10	peptidase	0.000173
dinoprost	chemical - endogenous mammalian	0.000176
alpha-estradiol	chemical - endogenous mammalian	0.000186
TGFB1	growth factor	0.000215
HIF1A	transcription regulator	0.000224
taxifolin	chemical - endogenous non-mammalian	0.000225
FSH	complex	0.000234
miR-27a-3p (and other miRNAs w/seed UCACAGU)	mature microRNA	0.000365
HNF4A	transcription regulator	0.000531
flufenamic acid	chemical drug	0.000785
tyrosine kinase	group	0.000859
WT1	transcription regulator	0.000887
puromycin	chemical - endogenous non-mammalian	0.00093

Table S3: Genes related to TP53 pathway that were downregulated in chronic WE through RNA-seq analysis

Symbol	Entrez Gene Name	Expr Fold Change	Location	Type(s)	Entrez Gene ID for Human
COQ8A	coenzyme Q8A	-1.81	Cytoplasm	kinase	56997
FGFR2	fibroblast growth factor receptor 2	-3.92	Plasma Membrane	kinase	2263
GADD45A	growth arrest and DNA damage inducible alpha	-1.5	Nucleus	other	1647
KAT2B	lysine acetyltransferase 2B	-1.52	Nucleus	transcription regulator	8850
PIAS1	protein inhibitor of activated STAT 1	-1.66	Nucleus	transcription regulator	8554
PIK3C3	phosphatidylinositol 3-kinase catalytic subunit type 3	-1.49	Cytoplasm	kinase	5289
PIK3CA	phosphatidylinositol-4,5-bisphosphate 3-kinase catalytic subunit alpha	-1.64	Cytoplasm	kinase	5290
PIK3CB	phosphatidylinositol-4,5-bisphosphate 3-kinase catalytic subunit beta	-1.86	Cytoplasm	kinase	5291
STAG1	stromal antigen 1	-1.46	Nucleus	transcription regulator	10274

Table S4: Genes belonging to EMT pathway hypermethylated and low abundant in chronic WE compared to normal skin

Hypermethylated	
Symbol	Entrez Gene Name
ADAM17	ADAM metallopeptidase domain 17
DVL1	dishevelled segment polarity protein 1
FGFR1	fibroblast growth factor receptor 1
FOXC2	forkhead box C2
HIF1A	hypoxia inducible factor 1 alpha subunit
KLB	klotho beta
KRAS	KRAS proto-oncogene, GTPase
LOX	lysyl oxidase
MAP2K3	mitogen-activated protein kinase kinase 3
MAP2K5	mitogen-activated protein kinase kinase 5
MET	MET proto-oncogene, receptor tyrosine kinase
mir-155	microRNA 155
NFKB2	nuclear factor kappa B subunit 2
NOTCH4	notch 4
PIK3R2	phosphoinositide-3-kinase regulatory subunit 2
RRAS	RAS related
RRAS2	RAS related 2
SMAD4	SMAD family member 4
SMURF1	SMAD specific E3 ubiquitin protein ligase 1
TCF4	transcription factor 4
TWIST1	twist family bHLH transcription factor 1
TWIST2	twist family bHLH transcription factor 2
WNT2	Wnt family member 2
WNT16	Wnt family member 16
WNT8B	Wnt family member 8B
WNT9A	Wnt family member 9A

Low expression	
**Symbol**	**Entrez Gene Name**
ADAM17	ADAM metallopeptidase domain 17
BRAF	B-Raf proto-oncogene, serine/threonine kinase
EGFR	epidermal growth factor receptor
FGF2	fibroblast growth factor 2
FGFR2	fibroblast growth factor receptor 2
FZD10	frizzled class receptor 10
PIK3C3	phosphatidylinositol 3-kinase catalytic subunit type 3
PIK3CA	phosphatidylinositol-4,5-bisphosphate 3-kinase catalytic subunit alpha
PIK3CB	phosphatidylinositol-4,5-bisphosphate 3-kinase catalytic subunit beta
SMURF1	SMAD specific E3 ubiquitin protein ligase 1
TWIST1	twist family bHLH transcription factor 1

Table S5: cluster 5 (kera 1) vs cluster 6 (kera 2) comparision; + means upregulated in cluster 5

Gene	p_val	avg_logFC	p_val_adj	Describtion
KRT1	2.5423E-218	3.128385814	6.6649E-214	keratin 1
KRT10	4.143E-192	2.941134864	1.0861E-187	keratin 10
DMKN	2.3226E-295	2.911094476	6.089E-291	dermokine
KRT5	7.7929E-292	2.864902733	2.043E-287	keratin 5
LGALS7B	1.4824E-291	2.824944467	3.8862E-287	galectin 7
KRT14	2.5128E-205	2.702580236	6.5875E-201	keratin 14
KRTDAP	3.6954E-141	2.45273742	9.6878E-137	keratinocyte differentiation associated protein
LY6D	1.7342E-298	2.426880012	4.5464E-294	lymphocyte antigen 6 family member D
DSP	0	2.392322267	0	desmoplakin
SFN	0	2.373954833	0	stratifin
DSC3	0	2.010354842	0	desmocollin 3
PERP	0	1.996891897	0	p53 apoptosis effector related to PMP22
CCL27	2.6172E-191	1.966346599	6.8612E-187	C-C motif chemokine ligand 27
AQP3	0	1.939206702	0	aquaporin 3 (Gill blood group)
CXCL14	7.7444E-272	1.931920639	2.0303E-267	C-X-C motif chemokine ligand 14
DST	8.6013E-169	1.923490951	2.2549E-164	dystonin
NFKBIA	0	1.82605017	0	NFKB inhibitor alpha
DSG1	5.203E-253	1.814735474	1.364E-248	desmoglein 1
RND3	1.8314E-292	1.804489003	4.8011E-288	Rho family GTPase 3
ZFP36L1	0	1.784170161	0	ZFP36 ring finger protein like 1
NEAT1	4.592E-275	1.776554977	1.2038E-270	nuclear paraspeckle assembly transcript 1
COL17A1	8.7409E-162	1.758832603	2.2915E-157	collagen type XVII alpha 1 chain
S100A14	8.628E-271	1.746595708	2.2619E-266	S100 calcium binding protein A14
SLC38A2	0	1.725596776	0	solute carrier family 38 member 2
SERPINB5	4.5826E-294	1.689940321	1.2014E-289	serpin family B member 5
EGR1	4.7354E-259	1.688249963	1.2414E-254	early growth response 1
LGALS7	2.2662E-152	1.683595367	5.941E-148	galectin 7
FOSB	9.02E-272	1.677605909	2.3647E-267	FosB proto-oncogene, AP-1 transcription factor subunit
ANXA1	3.8911E-304	1.649918505	1.0201E-299	annexin A1
INTS6	1.0266E-262	1.647869382	2.6913E-258	integrator complex subunit 6
DUSP1	3.2653E-271	1.647766597	8.5604E-267	dual specificity phosphatase 1
JUN	5.8762E-254	1.594443197	1.5405E-249	Jun proto-oncogene, AP-1 transcription factor subunit
HOPX	2.9461E-246	1.583354811	7.7235E-242	HOP homeobox
TNFAIP3	1.2489E-232	1.572187551	3.2742E-228	TNF alpha induced protein 3
ETS2	3.2523E-265	1.549399427	8.5262E-261	ETS proto-oncogene 2, transcription factor
KLF4	5.8841E-290	1.53841172	1.5426E-285	Kruppel like factor 4
MYC	1.8577E-250	1.538061198	4.8703E-246	MYC proto-oncogene, bHLH transcription factor
DNAJB1	4.0021E-278	1.525853977	1.0492E-273	DnaJ heat shock protein family (Hsp40) member B1
KLF5	1.6493E-283	1.523301319	4.3237E-279	Kruppel like factor 5
S100A2	3.2698E-132	1.50894048	8.572E-128	S100 calcium binding protein A2
PKP1	2.945E-242	1.490587487	7.7206E-238	plakophilin 1
MT1X	2.6129E-228	1.486867859	6.85E-224	metallothionein 1X
JUNB	3.0311E-276	1.479653963	7.9462E-272	JunB proto-oncogene, AP-1 transcription factor subunit
TACSTD2	7.3195E-225	1.47960008	1.9189E-220	tumor associated calcium signal transducer 2
PMAIP1	2.835E-231	1.476308886	7.4322E-227	phorbol-12-myristate-13-acetate-induced protein 1
ADRB2	4.5605E-225	1.469381171	1.1956E-220	adrenoceptor beta 2
AHNAK	1.7752E-272	1.461708532	4.6539E-268	AHNAK nucleoprotein
HSPB1	1.9635E-280	1.438036475	5.1476E-276	heat shock protein family B (small) member 1
GPNMB	1.6616E-251	1.433926243	4.356E-247	glycoprotein nmb
APOE	5.8376E-180	1.427522292	1.5304E-175	apolipoprotein E
FGFBP1	7.1417E-161	1.386946516	1.8723E-156	fibroblast growth factor binding protein 1
EMP1	1.2614E-237	1.360974116	3.3068E-233	epithelial membrane protein 1
SERPINB2	2.7218E-149	1.353677131	7.1355E-145	serpin family B member 2
CLDN1	1.8134E-187	1.341750232	4.754E-183	claudin 1
RORA	5.5876E-217	1.341391161	1.4649E-212	RAR related orphan receptor A
HLA-A	3.6234E-247	1.320879407	9.4991E-243	major histocompatibility complex, class I, A
PPP1R15A	1.1571E-254	1.316056654	3.0334E-250	protein phosphatase 1 regulatory subunit 15A
MAFB	5.4311E-198	1.302635412	1.4238E-193	MAF bZIP transcription factor B
NRARP	3.1182E-190	1.300996205	8.1746E-186	NOTCH regulated ankyrin repeat protein
GADD45A	1.3797E-169	1.291852581	3.6169E-165	growth arrest and DNA damage inducible alpha
DEGS1	1.9831E-226	1.291670615	5.199E-222	delta 4-desaturase, sphingolipid 1
MARCKS	1.2207E-224	1.286388938	3.2002E-220	myristoylated alanine rich protein kinase C substrate
MTRNR2L12	1.4451E-192	1.261907059	3.7886E-188	MT-RNR2 like 12
KLF6	3.9895E-208	1.253365958	1.0459E-203	Kruppel like factor 6
MT-CYB	1.3667E-210	1.242004648	3.583E-206	cytochrome b
B2M	8.5201E-283	1.23218084	2.2336E-278	beta-2-microglobulin
CRIP1	3.9058E-176	1.230008138	1.0239E-171	cysteine rich protein 1
MT-ND2	9.4549E-207	1.22862365	2.4787E-202	MTND2
PPP1R10	2.0576E-204	1.228437675	5.3941E-200	protein phosphatase 1 regulatory subunit 10
SBSN	3.49218E-87	1.218042984	9.15511E-83	suprabasin
SMG1	8.982E-208	1.214344341	2.3547E-203	SMG1 nonsense mediated mRNA decay associated PI3K related kinase
FUS	8.1566E-198	1.210524262	2.1383E-193	FUS RNA binding protein

BTG1	1.2178E-218	1.205265666	3.1925E-214	BTG anti-proliferation factor 1
FOS	3.1432E-184	1.203264389	8.2401E-180	Fos proto-oncogene, AP-1 transcription factor subunit
KLF10	1.4284E-202	1.192641073	3.7447E-198	Kruppel like factor 10
SYNE2	4.291E-192	1.189818672	1.1249E-187	spectrin repeat containing nuclear envelope protein 2
ID3	2.0141E-182	1.18957756	5.2802E-178	inhibitor of DNA binding 3, HLH protein
TRIM29	3.6941E-214	1.189494949	9.6844E-210	tripartite motif containing 29
RHOB	1.6476E-196	1.187345288	4.3193E-192	ras homolog family member B
BRD2	5.2021E-202	1.186805953	1.3638E-197	bromodomain containing 2
MCL1	4.5945E-217	1.177214229	1.2045E-212	MCL1 apoptosis regulator, BCL2 family member
GJA1	1.5722E-190	1.167653424	4.1217E-186	gap junction protein alpha 1
ANXA2	6.8346E-254	1.163019235	1.7918E-249	annexin A2
NFKBIZ	8.1388E-183	1.157200602	2.1337E-178	NFKB inhibitor zeta
AHNAK2	3.6822E-185	1.144951266	9.6533E-181	AHNAK nucleoprotein 2
TXNIP	4.9695E-156	1.13720137	1.3028E-151	thioredoxin interacting protein
MALAT1	1.2439E-197	1.136114862	3.2611E-193	metastasis associated lung adenocarcinoma transcript 1
EMP2	1.2379E-179	1.129802707	3.2453E-175	epithelial membrane protein 2
HLA-B	8.8141E-224	1.128009028	2.3107E-219	major histocompatibility complex, class I, B
BAZ1A	1.2905E-186	1.1252208	3.3833E-182	bromodomain adjacent to zinc finger domain 1A
XIST	2.7353E-187	1.111543915	7.1708E-183	X inactive specific transcript
RSRP1	1.7132E-200	1.106243517	4.4914E-196	arginine and serine rich protein 1
IFRD1	3.4551E-179	1.103663636	9.0579E-175	interferon related developmental regulator 1
MFSD2A	3.2168E-203	1.094809175	8.4332E-199	major facilitator superfamily domain containing 2A
MT-CO2	2.1273E-190	1.094124283	5.5768E-186	cytochrome c oxidase subunit II
SPAG9	3.5185E-177	1.092205836	9.224E-173	sperm associated antigen 9
HEXIM1	7.2226E-185	1.091257577	1.8935E-180	HEXIM P-TEFb complex subunit 1
REL	9.4361E-181	1.091206151	2.4738E-176	REL proto-oncogene, NF-kB subunit
CD55	1.3303E-161	1.089078503	3.4874E-157	CD55 molecule (Cromer blood group)
TLE4	6.6846E-185	1.072828127	1.7524E-180	TLE family member 4, transcriptional corepressor
JUP	2.9571E-183	1.071683749	7.7524E-179	junction plakoglobin
YWHAZ	5.1082E-211	1.066976571	1.3392E-206	tyrosine 3-monooxygenase/tryptophan 5-monooxygenase activation protein zeta
LYPD3	1.9498E-132	1.065031568	5.1116E-128	LY6/PLAUR domain containing 3
MT-ND1	9.0471E-185	1.056080598	2.3718E-180	NADH dehydrogenase, subunit 1 (complex I)
CCNL1	2.1179E-191	1.054460827	5.5522E-187	cyclin L1
ZFP36	1.5668E-192	1.05396743	4.1076E-188	ZFP36 ring finger protein
NDRG1	1.612E-169	1.053657283	4.226E-165	N-myc downstream regulated 1
FGFR3	5.4977E-186	1.051691964	1.4413E-181	fibroblast growth factor receptor 3
KRT17	2.83557E-30	1.050464985	7.43372E-26	keratin 17
MT-ATP6	4.7806E-178	1.048358269	1.2533E-173	ATP synthase F0 subunit 6
MT-CO3	3.4202E-175	1.04750532	8.9663E-171	cytochrome c oxidase III
TP63	2.4719E-187	1.0447928	6.4804E-183	tumor protein p63
DEFB1	8.3688E-130	1.038005059	2.194E-125	defensin beta 1
GATA3	2.4118E-159	1.036904331	6.3228E-155	GATA binding protein 3
IL18	9.0235E-187	1.035690768	2.3656E-182	interleukin 18
SYT8	6.3922E-130	1.034952282	1.6758E-125	synaptotagmin 8
ERRFI1	2.9064E-141	1.03407567	7.6194E-137	ERBB receptor feedback inhibitor 1
TAF1D	8.1665E-172	1.032249935	2.1409E-167	TATA-box binding protein associated factor, RNA polymerase I subunit D
MAF	1.536E-183	1.031172398	4.0267E-179	MAF bZIP transcription factor
PLP2	3.591E-198	1.027017413	9.4142E-194	proteolipid protein 2
MACF1	2.7047E-160	1.023387791	7.0907E-156	microtubule actin crosslinking factor 1
FABP5	2.73139E-60	1.020253669	7.16061E-56	fatty acid binding protein 5
MTRNR2L8	2.7339E-123	1.011212643	7.1673E-119	MT-RNR2 like 8
EXPH5	8.4779E-162	1.005288335	2.2226E-157	exophilin 5
PLK2	2.1878E-161	1.002954626	5.7356E-157	polo like kinase 2
DAPL1	4.5329E-168	0.99365852	1.1883E-163	death associated protein like 1
TRA2B	1.1363E-184	0.993649951	2.9789E-180	transformer 2 beta homolog
PLCG2	6.89438E-60	0.989579999	1.80743E-55	phospholipase C gamma 2
TIPARP	1.1994E-175	0.988209716	3.1445E-171	TCDD inducible poly(ADP-ribose) polymerase
BMP2	2.1504E-154	0.987835058	5.6374E-150	bone morphogenetic protein 2
BCL6	2.7018E-168	0.987808302	7.0831E-164	BCL6 transcription repressor
TENT5B	3.6633E-173	0.985934801	9.6038E-169	terminal nucleotidyltransferase 5B
DSG3	1.5149E-159	0.979247186	3.9715E-155	desmoglein 3
CLEC2B	8.0564E-172	0.976876641	2.1121E-167	C-type lectin domain family 2 member B
ID1	5.5003E-128	0.970842684	1.442E-123	inhibitor of DNA binding 1, HLH protein
ATP1B3	2.4273E-187	0.97057322	6.3633E-183	ATPase Na+/K+ transporting subunit beta 3
FBXW7	2.5146E-169	0.968735031	6.5922E-165	F-box and WD repeat domain containing 7
CSTA	2.1159E-135	0.961804051	5.547E-131	cystatin A
TPPP3	1.8548E-150	0.960358057	4.8625E-146	tubulin polymerization promoting protein family member 3
SFPQ	8.3948E-167	0.959310978	2.2008E-162	splicing factor proline and glutamine rich
KTN1	3.3449E-173	0.958376166	8.7689E-169	kinectin 1
TUBA1C	1.1218E-172	0.957062558	2.941E-168	tubulin alpha 1c
DENND2C	2.4221E-157	0.956965009	6.3499E-153	DENN domain containing 2C
HNRNPU	2.6801E-148	0.95426056	7.0261E-144	heterogeneous nuclear ribonucleoprotein U
KCNQ1OT1	9.591E-110	0.952953506	2.5144E-105	KCNQ1 opposite strand/antisense transcript 1
EZR	9.8451E-158	0.94968372	2.581E-153	ezrin
HLA-E	1.9623E-199	0.946561412	5.1443E-195	major histocompatibility complex, class I, E
HSPA5	4.7471E-163	0.942519088	1.2445E-158	heat shock protein family A (Hsp70) member 5

EPHB6	3.1977E-166	0.938215341	8.3832E-162	EPH receptor B6
HLA-C	5.4204E-163	0.935831054	1.421E-158	major histocompatibility complex, class I, C
NFE2L2	4.9389E-180	0.929966666	1.2948E-175	nuclear factor, erythroid 2 like 2
HES1	1.5437E-109	0.920876537	4.047E-105	hes family bHLH transcription factor 1
WSB1	1.3181E-159	0.92004678	3.4555E-155	WD repeat and SOCS box containing 1
DAAM1	8.1303E-157	0.912374718	2.1314E-152	dishevelled associated activator of morphogenesis 1
CDHR1	6.5365E-155	0.909611334	1.7136E-150	cadherin related family member 1
IFI27	1.76168E-42	0.905273925	4.61842E-38	interferon alpha inducible protein 27
HNRNPH1	4.0982E-147	0.904767401	1.0744E-142	heterogeneous nuclear ribonucleoprotein H1
TUBB2A	8.3105E-158	0.903766076	2.1787E-153	tubulin beta 2A class IIa
KRT16	3.95192E-56	0.902520696	1.03604E-51	keratin 16
DDX5	2.1174E-168	0.900180244	5.551E-164	DEAD-box helicase 5
AREG	6.79142E-65	0.8917195	1.78044E-60	amphiregulin
BTG2	3.8633E-143	0.891355213	1.0128E-138	BTG anti-proliferation factor 2
IER5	8.4696E-158	0.890963036	2.2204E-153	immediate early response 5
IRF6	2.0427E-158	0.889950926	5.3553E-154	interferon regulatory factor 6
ZNF217	1.9613E-122	0.889394133	5.1419E-118	zinc finger protein 217
RAP2B	1.3369E-142	0.884082405	3.5047E-138	RAP2B, member of RAS oncogene family
MT-ND5	2.6702E-158	0.881940272	7.0002E-154	NADH dehydrogenase, subunit 5 (complex I)
TRA2A	2.1755E-151	0.87410021	5.7034E-147	transformer 2 alpha homolog
DNAJA1	2.2939E-143	0.872259135	6.0136E-139	DnaJ heat shock protein family (Hsp40) member A1
SON	3.5578E-167	0.869364466	9.3271E-163	SON DNA and RNA binding protein
DDX3X	1.0386E-152	0.868609467	2.7227E-148	DEAD-box helicase 3 X-linked
IGFBP3	9.56697E-49	0.857632957	2.50808E-44	insulin like growth factor binding protein 3
MIDN	3.2296E-132	0.857540022	8.4667E-128	midnolin
MT-ND4	4.9533E-162	0.855013044	1.2986E-157	NADH dehydrogenase, subunit 4 (complex I)
CA12	4.1804E-145	0.854668401	1.0959E-140	carbonic anhydrase 12
PCF11	3.4233E-130	0.852968516	8.9746E-126	PCF11 cleavage and polyadenylation factor subunit
SLC2A1	8.0251E-145	0.851583027	2.1039E-140	solute carrier family 2 member 1
IFFO2	3.0676E-135	0.848236018	8.042E-131	intermediate filament family orphan 2
EPHA2	3.855E-129	0.84655673	1.0106E-124	EPH receptor A2
ZC3H12A	6.4447E-132	0.843354154	1.6895E-127	zinc finger CCCH-type containing 12A
WEE1	3.4411E-154	0.842584986	9.0211E-150	WEE1 G2 checkpoint kinase
CDKN1A	5.0161E-152	0.841755018	1.315E-147	cyclin dependent kinase inhibitor 1A
EGFR	7.4072E-145	0.840289453	1.9419E-140	epidermal growth factor receptor
PNRC1	4.2278E-163	0.8353148	1.1084E-158	proline rich nuclear receptor coactivator 1
MT1E	1.2147E-151	0.833321037	3.1844E-147	metallothionein 1E
KDM6B	1.1436E-125	0.83197925	2.9981E-121	lysine demethylase 6B
IER2	1.0271E-159	0.82441635	2.6927E-155	immediate early response 2
H2AFX	9.3051E-143	0.824134041	2.4394E-138	H2A.X variant histone
MT-ND3	8.3745E-148	0.823217778	2.1955E-143	NADH dehydrogenase, subunit 3 (complex I)
CCND1	5.1003E-111	0.81664846	1.3371E-106	cyclin D1
CKS2	9.585E-126	0.813940008	2.5128E-121	CDC28 protein kinase regulatory subunit 2
MT-CO1	1.2069E-161	0.813294809	3.164E-157	cytochrome c oxidase subunit I
ALDH3A2	2.6149E-141	0.810751284	6.8552E-137	aldehyde dehydrogenase 3 family member A2
PHLDA2	3.43854E-81	0.807264861	9.01447E-77	pleckstrin homology like domain family A member 2
PTP4A1	2.3853E-136	0.806243945	6.2534E-132	protein tyrosine phosphatase 4A1
PELI1	1.604E-118	0.802205402	4.205E-114	pellino E3 ubiquitin protein ligase 1
ANKRD12	4.4619E-139	0.799915155	1.1697E-134	ankyrin repeat domain 12
LIMA1	3.3287E-120	0.798505836	8.7266E-116	LIM domain and actin binding 1
RPL22L1	5.0335E-136	0.797088515	1.3196E-131	ribosomal protein L22 like 1
SDC1	2.6834E-122	0.79694923	7.0348E-118	syndecan 1
DSC1	9.24563E-81	0.795346732	2.42383E-76	desmocollin 1
CHD1	4.3784E-120	0.7940674	1.1478E-115	chromodomain helicase DNA binding protein 1
ARID5B	2.1515E-105	0.793241668	5.6403E-101	AT-rich interaction domain 5B
RPS20	4.0126E-131	0.792578624	1.0519E-126	ribosomal protein S20
SOCS3	1.40681E-92	0.786139912	3.68809E-88	suppressor of cytokine signaling 3
NUFIP2	2.4574E-138	0.78364015	6.4424E-134	nuclear FMR1 interacting protein 2
CSRNP1	2.5614E-128	0.779622889	6.715E-124	cysteine and serine rich nuclear protein 1
COL7A1	4.49841E-80	0.776146712	1.1793E-75	collagen type VII alpha 1 chain
ARL4D	1.3189E-124	0.773592651	3.4577E-120	ADP ribosylation factor like GTPase 4D
PRNP	2.005E-138	0.77066369	5.2564E-134	prion protein
IRF2BP2	1.3123E-143	0.766907041	3.4403E-139	interferon regulatory factor 2 binding protein 2
POU3F1	4.245E-131	0.763529355	1.1129E-126	POU class 3 homeobox 1
KLF13	1.6383E-141	0.760720734	4.2949E-137	Kruppel like factor 13
RPLP1	1.5297E-183	0.752037207	4.0103E-179	ribosomal protein lateral stalk subunit P1
TOB2	1.9225E-116	0.751611586	5.0399E-112	transducer of ERBB2, 2
TMEM154	5.5946E-118	0.747747701	1.4667E-113	transmembrane protein 154
CAV1	1.1973E-119	0.747586992	3.1389E-115	caveolin 1
GOLGA4	7.1686E-116	0.745706103	1.8793E-111	golgin A4
FOXQ1	2.9956E-115	0.744287345	7.8532E-111	forkhead box Q1
MT-ND4L	7.4959E-137	0.742970862	1.9651E-132	NADH dehydrogenase, subunit 4L (complex I)
C9orf3	6.8234E-136	0.742761911	1.7888E-131	aminopeptidase O (putative)
PABPC1	3.3192E-133	0.740782556	8.7016E-129	poly(A) binding protein cytoplasmic 1
SERTAD2	5.1279E-128	0.740340648	1.3443E-123	SERTA domain containing 2
DDIT4	2.6701E-116	0.738408557	7E-112	DNA damage inducible transcript 4

SNHG12	1.19448E-95	0.73515988	3.13144E-91	small nucleolar RNA host gene 12
THBD	1.50814E-83	0.733501756	3.95373E-79	thrombomodulin
SLC3A2	8.7263E-125	0.732124389	2.2877E-120	solute carrier family 3 member 2
SLC20A1	1.0591E-103	0.731662158	2.7767E-99	solute carrier family 20 member 1
TGFBI	2.07366E-97	0.728089861	5.4363E-93	transforming growth factor beta induced
TAGLN2	9.1232E-128	0.727335129	2.3917E-123	transgelin 2
PTPN13	1.3171E-128	0.726872245	3.4529E-124	protein tyrosine phosphatase non-receptor type 13
PPP1R14C	8.0672E-133	0.725200778	2.1149E-128	protein phosphatase 1 regulatory inhibitor subunit 14C
FOXN3	7.6304E-129	0.72381257	2.0004E-124	forkhead box N3
CXADR	6.0016E-129	0.723383004	1.5734E-124	CXADR Ig-like cell adhesion molecule
POSTN	2.50689E-50	0.722254322	6.57206E-46	periostin
MT4	3.34571E-66	0.721273337	8.77111E-62	metallothionein 4
RTN4	8.3944E-122	0.721225903	2.2007E-117	reticulon 4
ZFP36L2	2.3984E-128	0.721017985	6.2876E-124	ZFP36 ring finger protein like 2
EIF4G2	6.5729E-123	0.719038482	1.7232E-118	eukaryotic translation initiation factor 4 gamma 2
TGIF1	2.4375E-122	0.715981491	6.3902E-118	TGFB induced factor homeobox 1
CYLD	3.74793E-97	0.713363286	9.82556E-93	CYLD lysine 63 deubiquitinase
RBM39	3.6425E-132	0.712574076	9.5492E-128	RNA binding motif protein 39
PTMA	2.8491E-236	0.712100058	7.4691E-232	prothymosin alpha
AMD1	7.9831E-115	0.711260183	2.0928E-110	adenosylmethionine decarboxylase 1
LUC7L3	9.5074E-115	0.709634505	2.4925E-110	LUC7 like 3 pre-mRNA splicing factor
GOLGB1	7.6522E-111	0.70947312	2.0061E-106	golgin B1
APCDD1	3.6665E-112	0.707907607	9.6121E-108	APC down-regulated 1
TOR1AIP2	9.6742E-113	0.702552013	2.5362E-108	torsin 1A interacting protein 2
TOB1	1.2323E-112	0.702147795	3.2305E-108	transducer of ERBB2, 1
IRX2	1.6642E-107	0.700844221	4.3628E-103	iroquois homeobox 2
GJB3	8.1461E-126	0.699783312	2.1356E-121	gap junction protein beta 3
MBNL1	1.7728E-110	0.698708856	4.6475E-106	muscleblind like splicing regulator 1
LONRF1	6.415E-112	0.691603673	1.6817E-107	LON peptidase N-terminal domain and ring finger 1
MYCBP2	3.8988E-107	0.69151477	1.0221E-102	MYC binding protein 2
HSPH1	9.5928E-109	0.68979657	2.5149E-104	heat shock protein family H (Hsp110) member 1
TSC22D2	3.6324E-101	0.688068677	9.52266E-97	TSC22 domain family member 2
CLK1	1.0617E-114	0.684386846	2.7833E-110	CDC like kinase 1
C19orf33	1.4107E-108	0.683423273	3.6983E-104	chromosome 19 open reading frame 33
GSTM3	6.1801E-118	0.682892759	1.6202E-113	glutathione S-transferase mu 3
OVOL1	1.90945E-96	0.682446813	5.00582E-92	ovo like transcriptional repressor 1
PDLIM1	5.2238E-125	0.682152146	1.3695E-120	PDZ and LIM domain 1
ATP2B4	1.0322E-119	0.681232309	2.7061E-115	ATPase plasma membrane Ca2+ transporting 4
LDLR	4.7664E-102	0.68103751	1.24956E-97	low density lipoprotein receptor
NXF1	3.8946E-105	0.680045915	1.021E-100	nuclear RNA export factor 1
YBX3	3.8791E-130	0.678775175	1.0169E-125	Y-box binding protein 3
ATP2B1	9.9357E-109	0.678764055	2.6047E-104	ATPase plasma membrane Ca2+ transporting 1
CPEB2	1.8593E-103	0.6787469	4.8743E-99	cytoplasmic polyadenylation element binding protein 2
WNK1	7.8318E-115	0.677679664	2.0532E-110	WNK lysine deficient protein kinase 1
CYP3A5	1.52599E-88	0.669921012	4.00053E-84	cytochrome P450 family 3 subfamily A member 5
JAG1	7.2819E-115	0.668737711	1.909E-110	jagged canonical Notch ligand 1
MYLIP	3.3781E-110	0.666329081	8.8559E-106	myosin regulatory light chain interacting protein
TAF4B	7.21105E-91	0.665299044	1.89045E-86	TATA-box binding protein associated factor 4b
ATF4	6.9901E-115	0.66526192	1.8325E-110	activating transcription factor 4
ZBTB7A	4.2469E-110	0.664263553	1.1134E-105	zinc finger and BTB domain containing 7A
TNFRSF19	1.6285E-115	0.66285329	4.2694E-111	TNF receptor superfamily member 19
CASP14	3.57408E-91	0.662167971	9.3698E-87	caspase 14
NR1D1	2.8502E-100	0.660208832	7.47205E-96	nuclear receptor subfamily 1 group D member 1
BCL11B	4.1258E-108	0.65912055	1.0816E-103	BAF chromatin remodeling complex subunit BCL11B
NDFIP2	1.0533E-122	0.65896543	2.7612E-118	Nedd4 family interacting protein 2
PLS3	8.446E-128	0.657919049	2.2142E-123	plastin 3
ERF	2.32555E-83	0.653050205	6.09665E-79	ETS2 repressor factor
MAST4	1.7509E-105	0.652477236	4.5902E-101	microtubule associated serine/threonine kinase family member 4
EFNA1	1.6612E-122	0.651253541	4.3549E-118	ephrin A1
CSNK1A1	2.4606E-106	0.650411916	6.4506E-102	casein kinase 1 alpha 1
GATM	3.55723E-98	0.650316513	9.32562E-94	glycine amidinotransferase
BTG3	3.1439E-108	0.646730845	8.2421E-104	BTG anti-proliferation factor 3
TFAP2A	4.9778E-114	0.646720983	1.305E-109	transcription factor AP-2 alpha
LGALSL	1.1241E-100	0.645541247	2.94698E-96	galectin like
NABP1	1.92761E-81	0.643310395	5.05342E-77	nucleic acid binding protein 1
G3BP1	3.8059E-102	0.640987087	9.9776E-98	G3BP stress granule assembly factor 1
SNHG8	1.24225E-97	0.638576137	3.25668E-93	small nucleolar RNA host gene 8
NSG1	2.7045E-115	0.635463137	7.0901E-111	neuronal vesicle trafficking associated 1
CYR61	1.20075E-73	0.635248518	3.14788E-69	cellular communication network factor 1
EIF4A2	4.65444E-96	0.635105657	1.22021E-91	eukaryotic translation initiation factor 4A2
EIF3A	5.882E-107	0.634844659	1.542E-102	eukaryotic translation initiation factor 3 subunit A
NUPR1	6.18071E-89	0.630408103	1.62033E-84	nuclear protein 1, transcriptional regulator
IRS2	6.222E-102	0.630166923	1.63116E-97	insulin receptor substrate 2
BBC3	2.19356E-90	0.628721362	5.75063E-86	BCL2 binding component 3
WDR47	9.3181E-110	0.628677812	2.4428E-105	WD repeat domain 47
CAPG	2.3338E-121	0.627932695	6.1182E-117	capping actin protein, gelsolin like

ZBTB43	2.25871E-95	0.627911092	5.92144E-91	zinc finger and BTB domain containing 43
HNRNPC	7.4929E-106	0.626095323	1.9644E-101	heterogeneous nuclear ribonucleoprotein C
UBC	5.4699E-135	0.625739536	1.434E-130	ubiquitin C
GNAI1	1.6878E-118	0.625576633	4.4248E-114	G protein subunit alpha i1
RAPGEFL1	2.2593E-107	0.623270802	5.923E-103	Rap guanine nucleotide exchange factor like 1
ITPRIP	7.38707E-98	0.623218616	1.93659E-93	inositol 1,4,5-trisphosphate receptor interacting protein
SRSF2	3.5177E-99	0.622979167	9.22192E-95	serine and arginine rich splicing factor 2
BICD2	3.4172E-108	0.622722923	8.9586E-104	BICD cargo adaptor 2
CLCA2	1.6322E-105	0.621565004	4.279E-101	chloride channel accessory 2
HERPUD1	1.0601E-109	0.618964012	2.7791E-105	homocysteine inducible ER protein with ubiquitin like domain 1
SOX15	6.0445E-104	0.618131334	1.5846E-99	SRY-box transcription factor 15
HMGCS1	5.91234E-86	0.616897985	1.54998E-81	3-hydroxy-3-methylglutaryl-CoA synthase 1
EIF4A1	3.6067E-112	0.615317417	9.4554E-108	eukaryotic translation initiation factor 4A1
CCDC186	7.1005E-107	0.614812808	1.8615E-102	coiled-coil domain containing 186
ABI1	6.5841E-103	0.614081727	1.72609E-98	abl interactor 1
NECTIN1	1.1462E-109	0.613395516	3.0049E-105	nectin cell adhesion molecule 1
GPBP1	2.3124E-100	0.613264517	6.06209E-96	GC-rich promoter binding protein 1
MT2A	2.3963E-109	0.611275405	6.2822E-105	metallothionein 2A
HSPA2	1.29289E-97	0.610771344	3.38945E-93	heat shock protein family A (Hsp70) member 2
DDX21	9.261E-99	0.610292884	2.42787E-94	DExD-box helicase 21
AKAP9	1.3922E-103	0.610278432	3.6498E-99	A-kinase anchoring protein 9
PER1	4.87698E-86	0.608378322	1.27855E-81	period circadian regulator 1
ACTN4	3.1772E-110	0.607293793	8.3295E-106	actinin alpha 4
ETV3	1.9128E-104	0.605757607	5.0147E-100	ETS variant transcription factor 3
ITM2B	1.41378E-95	0.605705735	3.70637E-91	integral membrane protein 2B
KDSR	3.5639E-99	0.604611712	9.34325E-95	3-ketodihydrosphingosine reductase
IFI16	3.8119E-106	0.604324829	9.9932E-102	interferon gamma inducible protein 16
TRIM28	1.44153E-96	0.603855629	3.77912E-92	tripartite motif containing 28
CYB5R1	1.9412E-103	0.602122192	5.089E-99	cytochrome b5 reductase 1
KLHL21	3.10797E-79	0.60191746	8.14785E-75	kelch like family member 21
CEBPA	5.3298E-104	0.601273553	1.3973E-99	CCAAT enhancer binding protein alpha
KCNK7	1.88939E-86	0.600961857	4.95321E-82	potassium two pore domain channel subfamily K member 7
SF1	9.1586E-109	0.599625002	2.401E-104	splicing factor 1
CHD2	1.07338E-87	0.599238173	2.81398E-83	chromodomain helicase DNA binding protein 2
SPINK5	2.71908E-49	0.598582239	7.12834E-45	serine peptidase inhibitor Kazal type 5
DUSP7	2.1486E-101	0.595505724	5.63271E-97	dual specificity phosphatase 7
WFDC5	1.7809E-76	0.595013926	4.6688E-72	WAP four-disulfide core domain 5
MT1G	1.02544E-91	0.594397658	2.68829E-87	metallothionein 1G
PKP3	6.5564E-108	0.593582715	1.7188E-103	plakophilin 3
ZNF706	1.8165E-108	0.591372946	4.7622E-104	zinc finger protein 706
HBEGF	1.30496E-70	0.591020867	3.42108E-66	heparin binding EGF like growth factor
ARL5B	8.02363E-85	0.590858237	2.10347E-80	ADP ribosylation factor like GTPase 5B
ABLIM1	5.65529E-92	0.590454948	1.48259E-87	actin binding LIM protein 1
MED13L	3.38455E-93	0.587813376	8.87293E-89	mediator complex subunit 13L
TENT5A	1.32484E-86	0.58734035	3.47321E-82	terminal nucleotidyltransferase 5A
TNS4	7.79597E-85	0.585550564	2.04379E-80	tensin 4
EFNB2	3.22168E-85	0.585427339	8.44595E-81	ephrin B2
SPEN	1.07072E-81	0.584251419	2.807E-77	spen family transcriptional repressor
DYNLL1	1.52849E-90	0.581562297	4.00709E-86	dynein light chain LC8-type 1
RXRA	1.14422E-98	0.580944832	2.99968E-94	retinoid X receptor alpha
RPL23	9.24392E-83	0.580922223	2.42339E-78	ribosomal protein L23
QKI	1.42172E-95	0.580303183	3.72719E-91	QKI, KH domain containing RNA binding
CBX4	4.51694E-92	0.579236934	1.18416E-87	chromobox 4
NPEPPS	4.1922E-99	0.579044221	1.09903E-94	aminopeptidase puromycin sensitive
MAPK6	5.97452E-97	0.578887593	1.56628E-92	mitogen-activated protein kinase 6
MIR22HG	2.1347E-101	0.57692304	5.59622E-97	MIR22 host gene
CAPNS2	4.8439E-101	0.576843824	1.26986E-96	calpain small subunit 2
ELL2	5.53302E-78	0.576573818	1.45054E-73	elongation factor for RNA polymerase II 2
TINCR	5.2343E-108	0.574768336	1.3722E-103	TINCR ubiquitin domain containing
SQSTM1	1.16055E-74	0.572192643	3.0425E-70	sequestosome 1
BCLAF1	6.90559E-90	0.569579478	1.81037E-85	BCL2 associated transcription factor 1
C6orf62	2.58244E-85	0.568302348	6.77012E-81	chromosome 6 open reading frame 62
EPN2	5.6001E-104	0.56823092	1.4681E-99	epsin 2
IL1RN	6.38818E-96	0.567788627	1.67473E-91	interleukin 1 receptor antagonist
TOP1	1.90457E-86	0.567609692	4.99301E-82	DNA topoisomerase I
CHD4	2.45518E-84	0.566986243	6.4365E-80	chromodomain helicase DNA binding protein 4
EPPK1	3.26661E-68	0.566016294	8.56373E-64	epiplakin 1
MEF2A	1.22668E-80	0.565085399	3.21587E-76	myocyte enhancer factor 2A
MTDH	1.1777E-106	0.563018875	3.0876E-102	metadherin
ING1	7.3913E-104	0.562978846	1.9377E-99	inhibitor of growth family member 1
TUBA1A	5.38742E-90	0.562894199	1.41237E-85	tubulin alpha 1a
FLNA	4.94095E-82	0.562500486	1.29532E-77	filamin A
ITGA6	1.09911E-70	0.56169642	2.88142E-66	integrin subunit alpha 6
SYNCRIP	6.3004E-99	0.561501746	1.65171E-94	synaptotagmin binding cytoplasmic RNA interacting protein
HLA-F	1.4907E-104	0.561257763	3.9081E-100	major histocompatibility complex, class I, F
LMNA	3.66115E-94	0.561187605	9.59807E-90	lamin A/C

PLEC	3.17841E-91	0.560647929	8.33252E-87	plectin
CDH1	7.09423E-80	0.560396934	1.85982E-75	cadherin 1
ZNF207	3.18826E-95	0.560231845	8.35834E-91	zinc finger protein 207
RAB10	9.6269E-100	0.559202478	2.5238E-95	RAB10, member RAS oncogene family
KLF9	2.78221E-85	0.559054529	7.29385E-81	Kruppel like factor 9
GABPB1-AS1	5.31807E-65	0.558642288	1.39418E-60	GABPB1 antisense RNA 1
IL20RB	3.8935E-99	0.55763581	1.02073E-94	interleukin 20 receptor subunit beta
WNT4	1.29353E-89	0.557286916	3.39113E-85	Wnt family member 4
TMEM43	2.1759E-101	0.557136849	5.70438E-97	transmembrane protein 43
BAIAP2	8.68538E-94	0.555907173	2.27696E-89	BAR/IMD domain containing adaptor protein 2
GAS6	2.63476E-92	0.554593022	6.90728E-88	growth arrest specific 6
NFAT5	8.19717E-85	0.55457718	2.14897E-80	nuclear factor of activated T cells 5
YME1L1	8.36864E-93	0.554111465	2.19392E-88	YME1 like 1 ATPase
FOSL2	5.50263E-89	0.553276205	1.44257E-84	FOS like 2, AP-1 transcription factor subunit
DDX17	1.10886E-96	0.552613205	2.907E-92	DEAD-box helicase 17
BAG3	4.44054E-87	0.55245327	1.16413E-82	BAG cochaperone 3
GPC1	2.06269E-89	0.551465366	5.40755E-85	glypican 1
RBM25	1.39365E-94	0.551421581	3.6536E-90	RNA binding motif protein 25
ARID1B	1.89841E-91	0.551331016	4.97688E-87	AT-rich interaction domain 1B
SNHG15	2.31876E-91	0.551296006	6.07886E-87	small nucleolar RNA host gene 15
MSN	1.34715E-94	0.550959743	3.5317E-90	moesin
RPLP0	1.9657E-115	0.550399664	5.1534E-111	ribosomal protein lateral stalk subunit P0
SPPL3	3.15798E-93	0.550320587	8.27896E-89	signal peptide peptidase like 3
SRSF3	1.34453E-89	0.549499859	3.52481E-85	serine and arginine rich splicing factor 3
PRRC2C	1.5046E-104	0.547881603	3.9445E-100	proline rich coiled-coil 2C
EIF5	5.61099E-88	0.547094773	1.47098E-83	eukaryotic translation initiation factor 5
TFAP2C	1.51738E-95	0.545544599	3.97796E-91	transcription factor AP-2 gamma
IVNS1ABP	1.85559E-74	0.544611487	4.86462E-70	influenza virus NS1A binding protein
YY1	1.36259E-93	0.544151135	3.57217E-89	YY1 transcription factor
PCDH7	1.19204E-81	0.543831732	3.12504E-77	protocadherin 7
UBR5	1.51576E-77	0.543583777	3.97371E-73	ubiquitin protein ligase E3 component n-recognin 5
RBBP6	2.6081E-91	0.542688534	6.83739E-87	RB binding protein 6, ubiquitin ligase
ZFC3H1	6.4204E-79	0.539677756	1.68317E-74	zinc finger C3H1-type containing
PPP1R13L	5.7297E-101	0.538674581	1.50211E-96	protein phosphatase 1 regulatory subunit 13 like
KMT2E	8.8416E-96	0.538103677	2.31791E-91	lysine methyltransferase 2E (inactive)
CDKN2AIP	6.68058E-86	0.537949269	1.75138E-81	CDKN2A interacting protein
ITGB1	2.42329E-71	0.53691482	6.35291E-67	integrin subunit beta 1
S100A16	7.0331E-81	0.536895271	1.8438E-76	S100 calcium binding protein A16
ADM	2.75115E-69	0.536542207	7.21243E-65	adrenomedullin
SYTL1	2.3844E-100	0.536484202	6.25106E-96	synaptotagmin like 1
RB1CC1	1.09986E-87	0.536174805	2.88339E-83	RB1 inducible coiled-coil 1
IMPA2	4.33453E-96	0.535202064	1.13634E-91	inositol monophosphatase 2
POLR2A	1.513E-75	0.534892787	3.96648E-71	RNA polymerase II subunit A
NASP	1.36391E-90	0.534660257	3.57562E-86	nuclear autoantigenic sperm protein
DNAJB4	1.34556E-88	0.533663918	3.52752E-84	DnaJ heat shock protein family (Hsp40) member B4
LRRC8A	3.38082E-79	0.532925271	8.86315E-75	leucine rich repeat containing 8 VRAC subunit A
JMJD1C	3.31161E-81	0.532710875	8.68171E-77	jumonji domain containing 1C
LSP1	7.39094E-88	0.531807208	1.93761E-83	lymphocyte specific protein 1
CTNNBIP1	1.60362E-80	0.531281073	4.20406E-76	catenin beta interacting protein 1
AFF4	7.65759E-87	0.530972902	2.00751E-82	AF4/FMR2 family member 4
NAB1	8.05537E-90	0.529922571	2.1118E-85	NGFI-A binding protein 1
ZNF703	1.66191E-79	0.52912972	4.35686E-75	zinc finger protein 703
HBP1	9.11791E-96	0.527064144	2.39035E-91	HMG-box transcription factor 1
KMT2C	4.52865E-83	0.526554487	1.18723E-78	lysine methyltransferase 2C
MIR222HG	4.75943E-70	0.52646641	1.24773E-65	
ID2	4.70592E-67	0.525252908	1.2337E-62	inhibitor of DNA binding 2
PRRG4	5.58799E-85	0.524871902	1.46495E-80	proline rich and Gla domain 4
CHMP1B	4.71815E-86	0.524659398	1.23691E-81	charged multivesicular body protein 1B
HSP90AA1	2.72032E-76	0.523116636	7.13159E-72	heat shock protein 90 alpha family class A member 1
LAMA3	1.34591E-66	0.522232992	3.52843E-62	laminin subunit alpha 3
HNRNPAB	2.50743E-90	0.521511513	6.57348E-86	heterogeneous nuclear ribonucleoprotein A/B
BCL11A	1.29743E-83	0.518573676	3.40135E-79	BAF chromatin remodeling complex subunit BCL11A
TNKS1BP1	2.21056E-84	0.518219296	5.7952E-80	tankyrase 1 binding protein 1
TNFAIP8	6.30192E-79	0.517315794	1.65211E-74	TNF alpha induced protein 8
ZBTB11	1.02917E-87	0.516902133	2.69808E-83	zinc finger and BTB domain containing 11
EDN1	4.71146E-62	0.515424746	1.23516E-57	endothelin 1
RSRC2	1.15419E-86	0.515125501	3.02583E-82	arginine and serine rich coiled-coil 2
SRRM2	5.76868E-89	0.514417327	1.51232E-84	serine/arginine repetitive matrix 2
MAL2	1.38804E-70	0.513890325	3.63889E-66	mal, T cell differentiation protein 2 (gene/pseudogene)
MED13	9.80976E-84	0.513810806	2.57173E-79	mediator complex subunit 13
LUZP1	1.37407E-80	0.51363939	3.60226E-76	leucine zipper protein 1
LAMP1	5.41187E-84	0.512438683	1.41878E-79	lysosomal associated membrane protein 1
ARIH1	1.20518E-90	0.509512084	3.1595E-86	ariadne RBR E3 ubiquitin protein ligase 1
KDM3A	6.99798E-76	0.509394291	1.83459E-71	lysine demethylase 3A
NOLC1	1.03315E-74	0.508491958	2.70849E-70	nucleolar and coiled-body phosphoprotein 1
ZNF750	1.92893E-64	0.505715851	5.05688E-60	zinc finger protein 750

PNRC2	4.31158E-72	0.502803822	1.13032E-67	proline rich nuclear receptor coactivator 2
ELF1	1.54744E-89	0.50275875	4.05678E-85	E74 like ETS transcription factor 1
NR3C1	3.88261E-90	0.50269704	1.01786E-85	nuclear receptor subfamily 3 group C member 1
CREBRF	5.25602E-68	0.500977558	1.37792E-63	CREB3 regulatory factor
HNRNPA0	5.50244E-94	0.500344913	1.44252E-89	heterogeneous nuclear ribonucleoprotein A0
LYPLA1	1.46963E-08	-0.500809382	0.000385278	lysophospholipase 1
SOD2	1.24914E-21	-0.502911103	3.27474E-17	superoxide dismutase 2
SELENOH	3.5148E-10	-0.503843768	9.21441E-06	selenoprotein H
SLC25A6	3.79825E-36	-0.504139786	9.95749E-32	solute carrier family 25 member 6
GABARAPL1	7.8575E-18	-0.505075442	2.05992E-13	GABA type A receptor associated protein like 1
TPT1	3.8624E-118	-0.505273941	1.0126E-113	tumor protein, translationally-controlled 1
FDFT1	3.76011E-10	-0.505677634	9.8575E-06	farnesyl-diphosphate farnesyltransferase 1
ACAA1	3.18291E-16	-0.507821816	8.34431E-12	acetyl-CoA acyltransferase 1
LAMTOR5	3.4847E-08	-0.508733961	0.000913548	late endosomal/lysosomal adaptor, MAPK and MTOR activator 5
DDAH2	6.46027E-14	-0.509314385	1.69362E-09	dimethylarginine dimethylaminohydrolase 2
SERPINB1	3.82069E-07	-0.51077363	0.010016312	serpin family B member 1
RPL19	2.9063E-123	-0.511010874	7.6191E-119	ribosomal protein L19
CIB1	4.21901E-16	-0.513053888	1.10605E-11	calcium and integrin binding 1
PCBD1	2.87721E-15	-0.514598243	7.54291E-11	pterin-4 alpha-carbinolamine dehydratase 1
MRPL14	3.94662E-07	-0.514667377	0.010346468	mitochondrial ribosomal protein L14
RPS10	2.1036E-89	-0.515081289	5.51479E-85	ribosomal protein S10
PSMC5	1.6516E-08	-0.517430597	0.000432984	proteasome 26S subunit, ATPase 5
GLS	8.61275E-08	-0.519588244	0.00225792	glutaminase
SEC61G	1.24028E-13	-0.519669639	3.25151E-09	SEC61 translocon subunit gamma
PRDX6	6.13079E-16	-0.520366785	1.60725E-11	peroxiredoxin 6
TCEA3	4.76209E-12	-0.521230054	1.24843E-07	transcription elongation factor A3
NEDD8	4.63854E-16	-0.52225308	1.21604E-11	NEDD8 ubiquitin like modifier
POLR2I	4.10989E-10	-0.522677838	1.07745E-05	RNA polymerase II subunit I
COA1	2.61908E-27	-0.52332215	6.86617E-23	cytochrome c oxidase assembly factor 1 homolog
SEC11C	4.65806E-15	-0.523930043	1.22116E-10	SEC11 homolog C, signal peptidase complex subunit
NDUFB6	3.05173E-11	-0.524658859	8.00042E-07	NADH:ubiquinone oxidoreductase subunit B6
OST4	2.82134E-38	-0.524786126	7.39642E-34	oligosaccharyltransferase complex subunit 4, non-catalytic
CALM1	1.79907E-57	-0.525100545	4.71645E-53	calmodulin 1
NDUFA7	7.53145E-31	-0.527788728	1.97444E-26	NADH:ubiquinone oxidoreductase subunit A7
ALDOA	4.79327E-12	-0.527872383	1.2566E-07	aldolase, fructose-bisphosphate A
DNPH1	4.12081E-12	-0.527915374	1.08031E-07	2'-deoxynucleoside 5'-phosphate N-hydrolase 1
GTF3C6	6.88833E-12	-0.529246784	1.80585E-07	general transcription factor IIIC subunit 6
NAA38	2.69286E-11	-0.529556744	7.0596E-07	N-alpha-acetyltransferase 38, NatC auxiliary subunit
SEC61B	8.04779E-19	-0.53068236	2.10981E-14	SEC61 translocon subunit beta
FDPS	7.41114E-09	-0.532078388	0.00019429	farnesyl diphosphate synthase
S100A6	1.34302E-97	-0.532347322	3.52087E-93	S100 calcium binding protein A6
MRPL21	1.01073E-13	-0.533038707	2.64974E-09	mitochondrial ribosomal protein L21
MRPS15	4.29109E-14	-0.534070309	1.12495E-09	mitochondrial ribosomal protein S15
ANXA5	2.91577E-21	-0.534907288	7.64399E-17	annexin A5
FIS1	1.17306E-13	-0.535630959	3.0753E-09	fission, mitochondrial 1
TMA7	6.9373E-61	-0.53567058	1.81868E-56	translation machinery associated 7 homolog
CAMTA1	8.5204E-11	-0.535793323	2.23371E-06	calmodulin binding transcription activator 1
MRPS7	1.83744E-20	-0.536111966	4.81703E-16	mitochondrial ribosomal protein S7
FAM96A	1.14394E-26	-0.536575827	2.99896E-22	cytosolic iron-sulfur assembly component 2A
NDFIP1	8.84711E-12	-0.540322628	2.31936E-07	Nedd4 family interacting protein 1
CCNB1IP1	7.27391E-12	-0.540506225	1.90693E-07	cyclin B1 interacting protein 1
CLDN7	2.52534E-51	-0.542805938	6.62044E-47	claudin 7
POLR2K	1.10357E-10	-0.543748855	2.89311E-06	RNA polymerase II subunit K
TALDO1	7.8801E-12	-0.54446376	2.06585E-07	transaldolase 1
CAPNS1	1.02454E-24	-0.544696064	2.68593E-20	calpain small subunit 1
MRPL40	1.67339E-14	-0.549488914	4.38697E-10	mitochondrial ribosomal protein L40
GTF2A2	5.10796E-12	-0.550447308	1.3391E-07	general transcription factor IIA subunit 2
PGAM1	1.55945E-12	-0.552188006	4.08825E-08	phosphoglycerate mutase 1
SDCBP	5.4732E-15	-0.554004088	1.43485E-10	syndecan binding protein
HEBP2	1.16289E-17	-0.558260622	3.04862E-13	heme binding protein 2
CFL1	5.07665E-50	-0.558446916	1.33089E-45	cofilin 1
MRPL12	9.43278E-24	-0.558776174	2.4729E-19	mitochondrial ribosomal protein L12
SSU72	2.64685E-13	-0.560417094	6.93899E-09	SSU72 homolog, RNA polymerase II CTD phosphatase
SCP2	7.95855E-15	-0.562182598	2.08641E-10	sterol carrier protein 2
SUMO1	1.69296E-12	-0.562245929	4.43827E-08	small ubiquitin like modifier 1
SUCLA2	3.80945E-17	-0.562310155	9.98686E-13	succinate-CoA ligase ADP-forming subunit beta
MTIF3	3.27582E-10	-0.562389257	8.5879E-06	mitochondrial translational initiation factor 3
MORF4L1	1.61604E-17	-0.562860791	4.23661E-13	mortality factor 4 like 1
CA2	3.82475E-17	-0.566645502	1.0027E-12	carbonic anhydrase 2
SDHC	6.42583E-15	-0.567472408	1.68459E-10	succinate dehydrogenase complex subunit C
COPS9	7.22747E-16	-0.567480471	1.89475E-11	COP9 signalosome subunit 9
C7orf50	1.64784E-31	-0.56805317	4.31999E-27	chromosome 7 open reading frame 50
TMEM14B	1.74972E-13	-0.568583025	4.58707E-09	transmembrane protein 14B
WWC1	1.85043E-44	-0.569202808	4.85108E-40	WW and C2 domain containing 1
RABEP1	7.0807E-30	-0.569572437	1.85628E-25	rabaptin, RAB GTPase binding effector protein 1
EPCAM	1.54517E-39	-0.57223906	4.05081E-35	epithelial cell adhesion molecule

FAM114A1	1.17905E-35	-0.572565007	3.091E-31	family with sequence similarity 114 member A1
COX20	1.26794E-10	-0.572750211	3.32404E-06	cytochrome c oxidase assembly factor COX20
KCNN4	8.401E-106	-0.574056586	2.2024E-101	potassium calcium-activated channel subfamily N member 4
GMPR	6.3417E-88	-0.574461485	1.66254E-83	guanosine monophosphate reductase
PSMA7	8.89072E-24	-0.574966374	2.33079E-19	proteasome 20S subunit alpha 7
C1orf43	1.08329E-10	-0.575187181	2.83995E-06	chromosome 1 open reading frame 43
AP2M1	9.67178E-12	-0.576656236	2.53555E-07	adaptor related protein complex 2 subunit mu 1
SLC25A11	1.66991E-15	-0.580828315	4.37783E-11	solute carrier family 25 member 11
FMC1	7.68252E-22	-0.581074395	2.01405E-17	formation of mitochondrial complex V assembly factor 1 homolog
HMGN1	2.5718E-24	-0.582737073	6.74224E-20	high mobility group nucleosome binding domain 1
VDAC3	1.02717E-11	-0.583136848	2.69283E-07	voltage dependent anion channel 3
OAZ1	1.97214E-79	-0.583373116	5.17016E-75	ornithine decarboxylase antizyme 1
COX7A2L	2.18739E-14	-0.585215532	5.73445E-10	cytochrome c oxidase subunit 7A2 like
BLOC1S1	2.71164E-13	-0.585679999	7.10884E-09	biogenesis of lysosomal organelles complex 1 subunit 1
MB	8.059E-122	-0.586091504	2.1127E-117	myoglobin
FTH1	6.3638E-168	-0.586235971	1.6683E-163	ferritin heavy chain 1
MOCS2	8.5978E-36	-0.586263323	2.254E-31	molybdenum cofactor synthesis 2
SMIM37	2.54899E-11	-0.587063968	6.68243E-07	mitoregulin
LAMTOR1	1.3097E-14	-0.587463418	3.43352E-10	late endosomal/lysosomal adaptor, MAPK and MTOR activator 1
ARHGDIB	7.85687E-37	-0.587900602	2.05976E-32	Rho GDP dissociation inhibitor beta
METTL5	8.52638E-16	-0.588785602	2.23527E-11	methyltransferase like 5
PYGB	4.9553E-26	-0.589273033	1.29908E-21	glycogen phosphorylase B
GTF2H5	5.02151E-14	-0.589370959	1.31644E-09	general transcription factor IIH subunit 5
NDUFB11	3.32376E-26	-0.593868107	8.71357E-22	NADH:ubiquinone oxidoreductase subunit B11
PRDX1	4.15509E-67	-0.594389996	1.0893E-62	peroxiredoxin 1
HIGD2A	4.94534E-17	-0.594963271	1.29647E-12	HIG1 hypoxia inducible domain family member 2A
ARPC3	4.8225E-31	-0.598187392	1.26427E-26	actin related protein 2/3 complex subunit 3
SMIM1	2.3976E-120	-0.600854545	6.2856E-116	small integral membrane protein 1 (Vel blood group)
TMEM256	4.34404E-19	-0.601144556	1.13883E-14	transmembrane protein 256
CARD19	1.11751E-25	-0.601458512	2.92967E-21	caspase recruitment domain family member 19
CRACR2B	4.52712E-93	-0.6018158	1.18683E-88	calcium release activated channel regulator 2B
TIMM8B	6.2645E-18	-0.603574588	1.6423E-13	translocase of inner mitochondrial membrane 8 homolog B
AC009041.2	1.109E-120	-0.60401594	2.9073E-116	
CIRBP	1.37273E-54	-0.604184434	3.59875E-50	cold inducible RNA binding protein
ELF5	4.1387E-129	-0.606000762	1.085E-124	E74 like ETS transcription factor 5
METRN	3.07696E-58	-0.60610667	8.06656E-54	meteorin, glial cell differentiation regulator
PSMD8	1.07598E-19	-0.608374558	2.82078E-15	proteasome 26S subunit, non-ATPase 8
BEX4	3.74841E-36	-0.608753083	9.82684E-32	brain expressed X-linked 4
RNF152	4.23904E-27	-0.609029163	1.11131E-22	ring finger protein 152
LINC01554	2.702E-118	-0.609717237	7.0835E-114	long intergenic non-protein coding RNA 1554
HIBADH	1.37917E-50	-0.611775582	3.61564E-46	3-hydroxyisobutyrate dehydrogenase
GTF3A	5.66715E-16	-0.615599198	1.4857E-11	general transcription factor IIIA
TMEM219	3.11126E-20	-0.616919365	8.15647E-16	transmembrane protein 219
TOMM20	6.81991E-41	-0.618213384	1.78791E-36	translocase of outer mitochondrial membrane 20
STK32A	8.1439E-124	-0.619500633	2.135E-119	serine/threonine kinase 32A
ACP1	2.38429E-19	-0.619888647	6.25066E-15	acid phosphatase 1
SNCA	9.03493E-24	-0.624528779	2.3686E-19	synuclein alpha
CENPX	8.09649E-22	-0.627330352	2.12258E-17	centromere protein X
LSM4	8.85418E-23	-0.631846634	2.32121E-18	LSM4 homolog, U6 small nuclear RNA and mRNA degradation associated
CKB	8.40935E-22	-0.633195727	2.2046E-17	creatine kinase B
TSPO	1.96453E-74	-0.6333172	5.15021E-70	translocator protein
MRPL34	8.55977E-18	-0.634208857	2.24403E-13	mitochondrial ribosomal protein L34
GRHPR	1.85948E-18	-0.634376598	4.87483E-14	glyoxylate and hydroxypyruvate reductase
ANAPC11	1.95781E-21	-0.634941782	5.1326E-17	anaphase promoting complex subunit 11
GCSH	2.49767E-37	-0.635635282	6.54788E-33	glycine cleavage system protein H
SDHB	2.72777E-28	-0.636575405	7.15113E-24	succinate dehydrogenase complex iron sulfur subunit B
NOSTRIN	1.4954E-101	-0.638546853	3.92046E-97	nitric oxide synthase trafficking
ECI2	4.46973E-42	-0.638949599	1.17178E-37	enoyl-CoA delta isomerase 2
AP2S1	9.60926E-25	-0.639868473	2.51916E-20	adaptor related protein complex 2 subunit sigma 1
TMEM230	1.02992E-29	-0.640938268	2.70005E-25	transmembrane protein 230
GLO1	7.81815E-20	-0.641092842	2.04961E-15	glyoxalase I
GNG5	5.17439E-39	-0.64218245	1.35652E-34	G protein subunit gamma 5
SPTSSA	1.34485E-16	-0.643239696	3.52566E-12	serine palmitoyltransferase small subunit A
COMT	3.8622E-26	-0.643487102	1.01251E-21	catechol-O-methyltransferase
MSRB2	8.54577E-46	-0.64401172	2.24036E-41	methionine sulfoxide reductase B2
NDUFS2	4.89774E-21	-0.645328789	1.28399E-16	NADH:ubiquinone oxidoreductase core subunit S2
SH3YL1	3.13231E-19	-0.646459659	8.21167E-15	SH3 and SYLF domain containing 1
ZFYVE21	9.739E-22	-0.646503557	2.55318E-17	zinc finger FYVE-type containing 21
CYCS	1.24642E-38	-0.646653589	3.26762E-34	cytochrome c, somatic
SELENBP1	2.61558E-75	-0.646732687	6.85701E-71	selenium binding protein 1
GPR12	7.3352E-116	-0.646991741	1.923E-111	G protein-coupled receptor 12
DYNLRB1	2.66478E-18	-0.6477442	6.98599E-14	dynein light chain roadblock-type 1
CHMP2A	5.21983E-26	-0.651046201	1.36843E-21	charged multivesicular body protein 2A
CLTA	1.77972E-34	-0.651291033	4.66571E-30	clathrin light chain A
MRPL52	5.79901E-20	-0.652163805	1.52027E-15	mitochondrial ribosomal protein L52
NDUFV1	1.63717E-24	-0.652942989	4.292E-20	NADH:ubiquinone oxidoreductase core subunit V1

OLIG1	2.6061E-127	-0.654565391	6.8323E-123	oligodendrocyte transcription factor 1
RPS19BP1	6.02936E-22	-0.654925762	1.58066E-17	ribosomal protein S19 binding protein 1
NDUFC2	3.80795E-28	-0.655474905	9.98293E-24	NADH:ubiquinone oxidoreductase subunit C2
OSBPL1A	1.55422E-23	-0.655502903	4.07453E-19	oxysterol binding protein like 1A
SRSF9	1.38474E-27	-0.657713426	3.63024E-23	serine and arginine rich splicing factor 9
TXN2	1.71049E-22	-0.658229114	4.48421E-18	thioredoxin 2
NUDT8	1.05968E-32	-0.662914475	2.77805E-28	nudix hydrolase 8
S100A11	6.5028E-91	-0.663272402	1.70477E-86	S100 calcium binding protein A11
SNU13	2.43272E-35	-0.663370233	6.37761E-31	small nuclear ribonucleoprotein 13
TRIM2	2.0021E-114	-0.663464799	5.2488E-110	tripartite motif containing 2
SMS	2.65223E-32	-0.666906263	6.95309E-28	spermine synthase
ROPN1	2.8151E-133	-0.667666297	7.38E-129	rhophilin associated tail protein 1
APOO	9.79132E-74	-0.675861051	2.56689E-69	apolipoprotein O
ALKBH7	1.44609E-25	-0.677485073	3.79106E-21	alkB homolog 7
MRPL51	3.79065E-25	-0.678771847	9.93757E-21	mitochondrial ribosomal protein L51
UBE2M	8.0281E-25	-0.679945699	2.10465E-20	ubiquitin conjugating enzyme E2 M
GLRX	1.19998E-51	-0.687742639	3.14586E-47	glutaredoxin
GNG7	9.7914E-107	-0.696533174	2.5669E-102	G protein subunit gamma 7
LMO7-AS1	4.6159E-108	-0.698321822	1.2101E-103	LMO7 antisense RNA 1
CD59	3.09883E-08	-0.699777639	0.00081239	CD59 molecule (CD59 blood group)
MRPL20	1.32406E-29	-0.700620788	3.47114E-25	mitochondrial ribosomal protein L20
ATP6V0E2	1.5329E-91	-0.702772167	4.01865E-87	ATPase H+ transporting V0 subunit e2
DECR1	1.1098E-26	-0.703956432	2.90944E-22	2,4-dienoyl-CoA reductase 1
PRELID1	3.93426E-31	-0.709993753	1.0314E-26	PRELI domain containing 1
TM4SF1	7.30141E-15	-0.713133919	1.91414E-10	transmembrane 4 L six family member 1
COX6A1	2.31809E-59	-0.713887159	6.0771E-55	cytochrome c oxidase subunit 6A1
NDUFA12	5.95192E-28	-0.714525499	1.56036E-23	NADH:ubiquinone oxidoreductase subunit A12
PFDN5	1.8285E-124	-0.715736479	4.7937E-120	prefoldin subunit 5
DDT	1.70233E-36	-0.719653085	4.46283E-32	D-dopachrome tautomerase
NDUFB5	6.23797E-27	-0.722430057	1.63535E-22	NADH:ubiquinone oxidoreductase subunit B5
UQCRC2	2.72799E-29	-0.723678463	7.15169E-25	ubiquinol-cytochrome c reductase core protein 2
PSMB6	1.65354E-28	-0.724993846	4.33493E-24	proteasome 20S subunit beta 6
HIF1A	5.53828E-32	-0.725825991	1.45192E-27	hypoxia inducible factor 1 subunit alpha
SNRPN	1.42698E-48	-0.726111014	3.74096E-44	small nuclear ribonucleoprotein polypeptide N
LRRC26	2.1962E-160	-0.733352165	5.7576E-156	leucine rich repeat containing 26
PPIA	9.7018E-143	-0.734061195	2.5434E-138	peptidylprolyl isomerase A
LITAF	4.50408E-27	-0.735215455	1.18079E-22	lipopolysaccharide induced TNF factor
FRZB	3.4798E-108	-0.735880672	9.1226E-104	frizzled related protein
COPE	3.49095E-37	-0.736902292	9.15188E-33	COPI coat complex subunit epsilon
C1orf122	1.86961E-39	-0.737960988	4.90138E-35	chromosome 1 open reading frame 122
TMEM141	3.53083E-36	-0.739818988	9.25643E-32	transmembrane protein 141
ATP6V1F	2.27899E-32	-0.743670866	5.97461E-28	ATPase H+ transporting V1 subunit F
SDHD	3.1518E-32	-0.745680023	8.26276E-28	succinate dehydrogenase complex subunit D
PKM	7.19048E-43	-0.746579819	1.88506E-38	pyruvate kinase M1/2
PART1	9.59311E-69	-0.747924471	2.51493E-64	prostate androgen-regulated transcript 1
RWDD4	2.80049E-43	-0.751076524	7.34177E-39	RWD domain containing 4
VTI1B	1.02526E-37	-0.751409761	2.68783E-33	vesicle transport through interaction with t-SNAREs 1B
IDH2	1.21884E-47	-0.753593984	3.19531E-43	isocitrate dehydrogenase (NADP(+)) 2
ALDH9A1	9.69457E-42	-0.75487663	2.54153E-37	aldehyde dehydrogenase 9 family member A1
LMO4	2.18297E-45	-0.756129398	5.72287E-41	LIM domain only 4
TOMM7	1.7082E-123	-0.756754643	4.4782E-119	translocase of outer mitochondrial membrane 7
SURF1	1.8835E-42	-0.760478322	4.93778E-38	SURF1 cytochrome c oxidase assembly factor
FMNL1	2.26021E-79	-0.762630314	5.92538E-75	formin like 1
NDUFS3	6.36314E-30	-0.763016229	1.66816E-25	NADH:ubiquinone oxidoreductase core subunit S3
PGK1	5.17864E-37	-0.765986515	1.35763E-32	phosphoglycerate kinase 1
HSPE1	6.5236E-68	-0.768314318	1.71023E-63	heat shock protein family E (Hsp10) member 1
NDUFS4	1.42233E-35	-0.773968463	3.72879E-31	NADH:ubiquinone oxidoreductase subunit S4
ELF3	8.81951E-54	-0.774910421	2.31212E-49	E74 like ETS transcription factor 3
PPP1CB	5.54322E-57	-0.777068672	1.45321E-52	protein phosphatase 1 catalytic subunit beta
BRK1	5.61113E-36	-0.777382367	1.47101E-31	BRICK1 subunit of SCAR/WAVE actin nucleating complex
AC025154.2	1.7167E-170	-0.778426608	4.5006E-166	
NDUFA5	9.60466E-38	-0.778484648	2.51796E-33	NADH:ubiquinone oxidoreductase subunit A5
SRP14	1.3285E-115	-0.780818269	3.4828E-111	signal recognition particle 14
EID1	1.08125E-58	-0.781597949	2.8346E-54	EP300 interacting inhibitor of differentiation 1
CHPT1	3.39272E-60	-0.782202502	8.89437E-56	choline phosphotransferase 1
GHITM	1.3624E-30	-0.783702902	3.57167E-26	growth hormone inducible transmembrane protein
SCOC	2.03107E-38	-0.784766702	5.32464E-34	short coiled-coil protein
FMO5	6.5977E-135	-0.789918648	1.7297E-130	flavin containing dimethylaniline monoxygenase 5
CD24	3.90211E-77	-0.792444209	1.02298E-72	CD24 molecule
ARL6IP4	5.18432E-52	-0.793745543	1.35912E-47	ADP ribosylation factor like GTPase 6 interacting protein 4
CMPK1	6.76022E-35	-0.794206356	1.77226E-30	cytidine/uridine monophosphate kinase 1
EN1	1.41004E-98	-0.80318139	3.69657E-94	engrailed homeobox 1
HMGN3	7.69921E-43	-0.805145841	2.01842E-38	high mobility group nucleosomal binding domain 3
SAT1	2.31321E-28	-0.805354823	6.0643E-24	spermidine/spermine N1-acetyltransferase 1
PADI2	3.9621E-180	-0.805703677	1.0387E-175	peptidyl arginine deiminase 2
C2orf88	1.9378E-143	-0.806489314	5.0801E-139	chromosome 2 open reading frame 88

COX14	2.0858E-45	-0.810144766	5.46813E-41	cytochrome c oxidase assembly factor COX14
VDAC2	1.49537E-65	-0.81044598	3.92025E-61	voltage dependent anion channel 2
FBXO2	1.11476E-64	-0.810896765	2.92246E-60	F-box protein 2
ZNF428	2.15923E-69	-0.811119297	5.66063E-65	zinc finger protein 428
PHLDA1	4.63252E-69	-0.817603224	1.21446E-64	pleckstrin homology like domain family A member 1
NANS	1.15747E-55	-0.820443323	3.03442E-51	N-acetylneuraminate synthase
MRAS	5.9333E-142	-0.828243743	1.5555E-137	muscle RAS oncogene homolog
DSTN	2.6166E-151	-0.83214433	6.8598E-147	destrin, actin depolymerizing factor
NDUFA8	5.68848E-49	-0.832306092	1.49129E-44	NADH:ubiquinone oxidoreductase subunit A8
AURKAIP1	1.27776E-52	-0.833646713	3.34977E-48	aurora kinase A interacting protein 1
HSBP1	9.2444E-49	-0.836098582	2.42351E-44	heat shock factor binding protein 1
S100A13	1.86806E-60	-0.841332607	4.89731E-56	S100 calcium binding protein A13
FKBP8	1.47434E-50	-0.842232544	3.86512E-46	FKBP prolyl isomerase 8
ESRRA	3.31426E-60	-0.848895468	8.68867E-56	estrogen related receptor alpha
ERH	1.69399E-62	-0.848921773	4.44097E-58	ERH mRNA splicing and mitosis factor
BNIP3	2.14168E-72	-0.849440516	5.61463E-68	BCL2 interacting protein 3
C19orf70	3.10574E-51	-0.851700019	8.14201E-47	mitochondrial contact site and cristae organizing system subunit 13
MRPL33	1.28557E-56	-0.855687303	3.37026E-52	mitochondrial ribosomal protein L33
PYURF	7.22088E-44	-0.859366478	1.89303E-39	PIGY upstream reading frame
SRP9	3.69793E-66	-0.860129385	9.69449E-62	signal recognition particle 9
FKBP4	1.68942E-31	-0.861487086	4.42899E-27	FKBP prolyl isomerase 4
GLRX5	8.66291E-47	-0.862171981	2.27107E-42	glutaredoxin 5
MPC2	2.95844E-52	-0.86993268	7.75585E-48	mitochondrial pyruvate carrier 2
HPRT1	7.98603E-79	-0.872544521	2.09362E-74	hypoxanthine phosphoribosyltransferase 1
LGALS1	1.33092E-75	-0.876034035	3.48914E-71	galectin 1
MCRIP2	8.0134E-134	-0.876794199	2.1008E-129	MAPK regulated corepressor interacting protein 2
NDUFA13	2.75315E-63	-0.879172168	7.21767E-59	NADH:ubiquinone oxidoreductase subunit A13
SPTSSB	1.772E-109	-0.879906054	4.6454E-105	serine palmitoyltransferase small subunit B
ANAPC16	2.0205E-58	-0.883666775	5.29694E-54	anaphase promoting complex subunit 16
NDUFA2	2.90889E-63	-0.885248217	7.62596E-59	NADH:ubiquinone oxidoreductase subunit A2
CYC1	3.58234E-53	-0.886154584	9.39145E-49	cytochrome c1
MPC1	1.71659E-52	-0.886777103	4.5002E-48	mitochondrial pyruvate carrier 1
NIPSNAP2	1.55341E-65	-0.888196595	4.07241E-61	nipsnap homolog 2
MDH2	4.7937E-61	-0.890059914	1.25672E-56	malate dehydrogenase 2
REXO2	1.256E-44	-0.896828646	3.29274E-40	RNA exonuclease 2
SKP1	6.4903E-124	-0.898039186	1.7015E-119	S-phase kinase associated protein 1
PNKD	2.55921E-59	-0.900575908	6.70922E-55	PNKD metallo-beta-lactamase domain containing
POLR2L	1.2749E-102	-0.904110339	3.3422E-98	RNA polymerase II subunit L
BEX3	8.80138E-66	-0.904309979	2.30737E-61	brain expressed X-linked 3
NDUFA11	1.97196E-70	-0.904402547	5.1697E-66	NADH:ubiquinone oxidoreductase subunit A11
CCNI	1.12872E-94	-0.910887587	2.95906E-90	cyclin I
MRPL41	2.28285E-75	-0.913644614	5.98472E-71	mitochondrial ribosomal protein L41
ATP6V1G1	6.51721E-89	-0.914804908	1.70855E-84	ATPase H+ transporting V1 subunit G1
UBL5	2.2193E-118	-0.916940182	5.8181E-114	ubiquitin like 5
DYNLT1	1.88382E-57	-0.918571821	4.93861E-53	dynein light chain Tctex-type 1
TPI1	5.3181E-91	-0.927281376	1.39419E-86	triosephosphate isomerase 1
HINT2	9.66065E-83	-0.934164722	2.53264E-78	histidine triad nucleotide binding protein 2
STK39	1.7669E-147	-0.941326358	4.6322E-143	serine/threonine kinase 39
SH3BGRL2	1.1716E-163	-0.943878941	3.0714E-159	SH3 domain binding glutamate rich protein like 2
SMIM22	1.929E-209	-0.946790541	5.0571E-205	small integral membrane protein 22
AC011247.1	1.9224E-176	-0.950011036	5.0399E-172	
NDUFS6	3.86852E-82	-0.953874637	1.01417E-77	NADH:ubiquinone oxidoreductase subunit S6
VAMP8	3.24203E-85	-0.96126804	8.49931E-81	vesicle associated membrane protein 8
ATP1B1	8.97603E-29	-0.968712245	2.35316E-24	ATPase Na+/K+ transporting subunit beta 1
HOMER2	2.6302E-166	-0.97375259	6.8954E-162	homer scaffold protein 2
PRDX2	2.61655E-98	-0.974371255	6.85954E-94	peroxiredoxin 2
EIF3K	6.6325E-140	-0.976893381	1.7388E-135	eukaryotic translation initiation factor 3 subunit K
NDUFS7	2.70662E-69	-0.979938391	7.09568E-65	NADH:ubiquinone oxidoreductase core subunit S7
GNAS	1.8969E-149	-0.981651185	4.973E-145	GNAS complex locus
GABARAPL2	3.29702E-95	-0.981869701	8.64348E-91	GABA type A receptor associated protein like 2
CNN3	7.8918E-108	-0.988145126	2.0689E-103	calponin 3
MTCH1	2.18152E-69	-0.989112402	5.71907E-65	mitochondrial carrier 1
COX4I1	7.1549E-175	-0.989888146	1.8757E-170	cytochrome c oxidase subunit 4I1
MDH1	9.08632E-64	-0.989983678	2.38207E-59	malate dehydrogenase 1
AES	2.46583E-88	-0.990292164	6.46441E-84	TLE family member 5, transcriptional modulator
NDUFAF3	1.51055E-91	-0.994430297	3.96005E-87	NADH:ubiquinone oxidoreductase complex assembly factor 3
ATP5MC2	6.0438E-182	-0.999644266	1.5845E-177	ATP synthase membrane subunit c locus 2
PARK7	2.49345E-90	-1.002364636	6.53684E-86	Parkinsonism associated deglycase
PRDX3	2.64375E-89	-1.002381381	6.93085E-85	peroxiredoxin 3
ATP5MC1	1.3969E-102	-1.005859912	3.66214E-98	ATP synthase membrane subunit c locus 1
HIGD1A	1.21259E-88	-1.009324334	3.17893E-84	HIG1 hypoxia inducible domain family member 1A
MGST3	6.656E-107	-1.015363866	1.7449E-102	microsomal glutathione S-transferase 3
NDUFV2	2.2012E-99	-1.016567468	5.77079E-95	NADH:ubiquinone oxidoreductase core subunit V2
SMDT1	2.49862E-86	-1.025166615	6.55037E-82	single-pass membrane protein with aspartate rich tail 1
PDK3	8.0274E-174	-1.029124763	2.1045E-169	pyruvate dehydrogenase kinase 3
NDUFB4	8.3048E-123	-1.032922461	2.1772E-118	NADH:ubiquinone oxidoreductase subunit B4

SUCLG1	1.89068E-85	-1.037535374	4.95659E-81	succinate-CoA ligase GDP/ADP-forming subunit alpha
UQCRFS1	1.43506E-88	-1.039690387	3.76216E-84	ubiquinol-cytochrome c reductase, Rieske iron-sulfur polypeptide 1
SLIRP	1.7332E-88	-1.04504283	4.54375E-84	SRA stem-loop interacting RNA binding protein
AC007906.2	1.7878E-121	-1.047174704	4.6869E-117	
ATP5PD	8.1099E-118	-1.049130998	2.1261E-113	ATP synthase peripheral stalk subunit d
SPINT1-AS1	1.61707E-94	-1.050845792	4.23931E-90	SPINT1 antisense RNA 1
ACTR3B	1.6178E-179	-1.052370797	4.2412E-175	actin related protein 3B
RPS27L	3.28655E-94	-1.052919543	8.61601E-90	ribosomal protein S27 like
HDDC2	4.8874E-99	-1.054204792	1.28128E-94	HD domain containing 2
NDUFB10	2.03823E-91	-1.056509844	5.34343E-87	NADH:ubiquinone oxidoreductase subunit B10
NDUFB9	8.78791E-88	-1.062303393	2.30384E-83	NADH:ubiquinone oxidoreductase subunit B9
ID4	6.70328E-71	-1.064363714	1.75733E-66	inhibitor of DNA binding 4, HLH protein
ECHS1	8.96856E-87	-1.066074102	2.3512E-82	enoyl-CoA hydratase, short chain 1
ATP5ME	2.9114E-131	-1.073951371	7.6326E-127	ATP synthase membrane subunit e
ATP5F1A	6.1306E-110	-1.076045386	1.6072E-105	ATP synthase F1 subunit alpha
ATP5MF	7.5248E-129	-1.087506145	1.9727E-124	ATP synthase membrane subunit f
TSTD1	7.09906E-94	-1.090969657	1.86109E-89	thiosulfate sulfurtransferase like domain containing 1
PDHA1	4.5597E-100	-1.090983097	1.19537E-95	pyruvate dehydrogenase E1 subunit alpha 1
MINOS1	1.8504E-108	-1.093924294	4.851E-104	mitochondrial contact site and cristae organizing system subunit 10
MIEN1	4.87897E-95	-1.095473284	1.27907E-90	migration and invasion enhancer 1
NDUFS8	2.40038E-90	-1.102362831	6.29285E-86	NADH:ubiquinone oxidoreductase core subunit S8
ATP5PO	2.2117E-106	-1.10265651	5.7982E-102	ATP synthase peripheral stalk subunit OSCP
VDAC1	9.5754E-106	-1.102980631	2.5103E-101	voltage dependent anion channel 1
ATP5F1D	2.2941E-152	-1.106670538	6.0143E-148	ATP synthase F1 subunit delta
BOLA3	2.8777E-119	-1.111571163	7.5441E-115	bolA family member 3
COX17	2.95941E-89	-1.114776245	7.75839E-85	cytochrome c oxidase copper chaperone COX17
NDUFB8	2.9857E-108	-1.119887575	7.8274E-104	NADH:ubiquinone oxidoreductase subunit B8
ATP5PF	1.0379E-148	-1.12475192	2.7209E-144	ATP synthase peripheral stalk subunit F6
SERF2	9.2408E-248	-1.12744852	2.4226E-243	small EDRK-rich factor 2
PRDX5	4.1685E-126	-1.13390291	1.0928E-121	peroxiredoxin 5
NDUFB2	6.4067E-132	-1.137522309	1.6796E-127	NADH:ubiquinone oxidoreductase subunit B2
CHCHD2	2.0651E-202	-1.138516818	5.4138E-198	coiled-coil-helix-coiled-coil-helix domain containing 2
CITED4	2.08678E-86	-1.148355756	5.4707E-82	Cbp/p300 interacting transactivator with Glu/Asp rich carboxy-terminal domain 4
PPA1	6.6452E-123	-1.155893269	1.7421E-118	inorganic pyrophosphatase 1
HINT1	1.047E-230	-1.1606951	2.7449E-226	histidine triad nucleotide binding protein 1
ENO1	4.6391E-149	-1.162155104	1.2162E-144	enolase 1
ECI1	2.2066E-128	-1.163674302	5.7847E-124	enoyl-CoA delta isomerase 1
NDUFAB1	1.4418E-131	-1.173228626	3.7799E-127	NADH:ubiquinone oxidoreductase subunit AB1
ATP5F1E	1.0627E-269	-1.175629061	2.786E-265	ATP synthase F1 subunit epsilon
NDUFA3	8.3707E-114	-1.17942351	2.1945E-109	NADH:ubiquinone oxidoreductase subunit A3
ADI1	1.9569E-102	-1.1809509	5.13027E-98	acireductone dioxygenase 1
COX6B1	1.161E-180	-1.183205643	3.0436E-176	cytochrome c oxidase subunit 6B1
ADIRF	1.1476E-158	-1.189475371	3.0085E-154	adipogenesis regulatory factor
UQCR10	5.589E-153	-1.199361463	1.4652E-148	ubiquinol-cytochrome c reductase, complex III subunit X
NDUFA6	1.2745E-119	-1.203747185	3.3413E-115	NADH:ubiquinone oxidoreductase subunit A6
SLC25A5	2.6257E-184	-1.208046447	6.8836E-180	solute carrier family 25 member 5
RCAN2	3.2649E-206	-1.215836052	8.5593E-202	regulator of calcineurin 2
ISCU	4.7767E-122	-1.216479626	1.2523E-117	iron-sulfur cluster assembly enzyme
COX5A	6.108E-124	-1.218311145	1.6013E-119	cytochrome c oxidase subunit 5A
ROMO1	4.3424E-124	-1.218747302	1.1384E-119	reactive oxygen species modulator 1
UQCRQ	7.8448E-167	-1.220317794	2.0566E-162	ubiquinol-cytochrome c reductase complex III subunit VII
GOT1	2.1704E-150	-1.225659071	5.6898E-146	glutamic-oxaloacetic transaminase 1
NDUFS5	3.0211E-182	-1.225712901	7.9202E-178	NADH:ubiquinone oxidoreductase subunit S5
SLC25A3	1.4895E-192	-1.230244238	3.9049E-188	solute carrier family 25 member 3
ETHE1	4.0594E-161	-1.233318762	1.0642E-156	ETHE1 persulfide dioxygenase
ATP5MPL	5.2318E-146	-1.236850366	1.3716E-141	ATP synthase membrane subunit 6.8PL
CYSTM1	3.3768E-166	-1.239810276	8.8526E-162	cysteine rich transmembrane module containing 1
NDUFB7	8.5603E-143	-1.241209224	2.2442E-138	NADH:ubiquinone oxidoreductase subunit B7
QDPR	4.9814E-180	-1.247188389	1.3059E-175	quinoid dihydropteridine reductase
COX6C	5.4349E-227	-1.25973951	1.4248E-222	cytochrome c oxidase subunit 6C
CALM2	2.7698E-236	-1.265650854	7.2613E-232	calmodulin 1
ATP5MD	9.7181E-171	-1.280521488	2.5477E-166	ATP synthase membrane subunit DAPIT
PPDPF	2.4988E-239	-1.291438953	6.551E-235	pancreatic progenitor cell differentiation and proliferation factor
UQCRB	8.4568E-227	-1.295493771	2.217E-222	ubiquinol-cytochrome c reductase binding protein
UQCRH	1.6146E-208	-1.295713813	4.2327E-204	ubiquinol-cytochrome c reductase hinge protein
NDUFB3	5.5643E-149	-1.301156855	1.4587E-144	NADH:ubiquinone oxidoreductase subunit B3
COX7A2	2.0939E-223	-1.305094013	5.4894E-219	cytochrome c oxidase subunit 7A2
NDUFC1	4.7751E-165	-1.306540766	1.2518E-160	NADH:ubiquinone oxidoreductase subunit C1
SLC25A4	3.6194E-203	-1.313733108	9.4887E-199	solute carrier family 25 member 4
GCHFR	1.6968E-172	-1.319644016	4.4483E-168	GTP cyclohydrolase I feedback regulator
PHB	8.6443E-142	-1.32661012	2.2662E-137	prohibitin
GPX4	7.4293E-206	-1.337824729	1.9477E-201	glutathione peroxidase 4
AZGP1	2.9816E-96	-1.380060372	7.81656E-92	alpha-2-glycoprotein 1, zinc-binding
CA6	5.209E-267	-1.389233173	1.3656E-262	carbonic anhydrase 6
CLDN10	3.7382E-202	-1.394512531	9.8001E-198	claudin 10
COX8A	2.3707E-230	-1.401617524	6.215E-226	cytochrome c oxidase subunit 8A

NDUFB1	2.8889E-193	-1.426116396	7.5734E-189	NADH:ubiquinone oxidoreductase subunit B1
COX7B	7.7619E-207	-1.431887622	2.0349E-202	cytochrome c oxidase subunit 7B
UQCR11	1.3342E-206	-1.434101793	3.4978E-202	ubiquinol-cytochrome c reductase, complex III subunit XI
ATP5F1B	7.7939E-191	-1.437848323	2.0432E-186	ATP synthase F1 subunit beta
NDUFA1	3.0613E-226	-1.45058793	8.0255E-222	NADH:ubiquinone oxidoreductase subunit A1
ROPN1B	0	-1.488373444	0	rhophilin associated tail protein 1B
ATP5MG	1.0076E-266	-1.491234192	2.6414E-262	ATP synthase membrane subunit g
DCXR	7.3058E-205	-1.542512334	1.9153E-200	dicarbonyl and L-xylulose reductase
ZG16B	6.39113E-88	-1.544997691	1.6755E-83	zymogen granule protein 16B
ATP5F1C	1.4634E-203	-1.551908599	3.8363E-199	ATP synthase F1 subunit gamma
STAC2	0	-1.556499529	0	SH3 and cysteine rich domain 2
SFRP1	9.3491E-139	-1.55970355	2.451E-134	secreted frizzled related protein 1
COX5B	4.7112E-260	-1.560049315	1.2351E-255	cytochrome c oxidase subunit 5B
PEBP1	2.5085E-257	-1.586634018	6.5763E-253	phosphatidylethanolamine binding protein 1
LDHA	1.2246E-222	-1.594946811	3.2105E-218	lactate dehydrogenase A
PDCD4	4.1775E-210	-1.596713942	1.0952E-205	programmed cell death 4
ETFB	1.1861E-203	-1.61847729	3.1096E-199	electron transfer flavoprotein subunit beta
NDUFA4	1.2078E-294	-1.621379426	3.1663E-290	NDUFA4 mitochondrial complex associated
SLC12A2	6.68391E-46	-1.631237211	1.75225E-41	solute carrier family 12 member 2
ELOB	1.6677E-284	-1.63521184	4.3721E-280	elongin B
COX7C	2.3982E-292	-1.658324089	6.2872E-288	cytochrome c oxidase subunit 7C
AKR1C1	2.1189E-216	-1.677747212	5.5549E-212	aldo-keto reductase family 1 member C2
NDRG2	1.8917E-260	-1.73628469	4.9593E-256	NDRG family member 2
NCALD	0	-1.754261678	0	neurocalcin delta
TPD52L1	1.444E-304	-1.803515831	3.7857E-300	TPD52 like 1
DBI	2.8706E-297	-1.871212838	7.5256E-293	diazepam binding inhibitor, acyl-CoA binding protein
ATP5MC3	1.3149E-292	-1.903568668	3.4473E-288	ATP synthase membrane subunit c locus 3
CSRP1	0	-1.944564098	0	cysteine and glycine rich protein 1
COX7A1	0	-1.95084576	0	cytochrome c oxidase subunit 7A1
TESC	0	-2.077593806	0	tescalcin
CHCHD10	0	-2.103842131	0	coiled-coil-helix-coiled-coil-helix domain containing 10
KRT8	0	-2.149572927	0	keratin 8
GAPDH	3.4074E-281	-2.226090632	8.9329E-277	glyceraldehyde-3-phosphate dehydrogenase
PPP1R1B	0	-2.288001771	0	protein phosphatase 1 regulatory inhibitor subunit 1B
CAMK2N1	0	-2.288524557	0	calcium/calmodulin dependent protein kinase II inhibitor 1
S100A1	0	-2.373223914	0	S100 calcium binding protein A1
KRT19	0	-2.482586791	0	keratin 19
KRT18	0	-2.486532004	0	keratin 18
KRT7	0	-2.517890837	0	keratin 7
SNORC	0	-2.539399458	0	secondary ossification center associated regulator of chondrocyte maturation
SCGB1D2	3.16767E-44	-2.819998113	8.30435E-40	secretoglobin family 1D member 2
AQP5	0	-2.879641773	0	aquaporin 5
SCGB2A2	4.377E-71	-3.38446038	1.14748E-66	secretoglobin family 2A member 2
MUCL1	2.01049E-74	-3.402440739	5.2707E-70	mucin like 1
DCD	1.40087E-89	-3.494107754	3.67253E-85	dermcidin
HBB	1.85086E-44	-3.581896071	4.8522E-40	hemoglobin subunit beta

Table S6: chronic WE vs unwounded skin comparison; + means upregulated in chronic WE

Gene	p_val	avg_logFC	p_val_adj	Description
S100A8	0	3.558167231	0	S100 calcium binding protein A8
S100A9	0	3.408363162	0	S100 calcium binding protein A9
KRT6B	0	3.361669662	0	keratin 6B
KRT6C	0	3.27519539	0	keratin 6C
KRT6A	0	3.227283044	0	keratin 6A
KRT16	0	3.217679454	0	keratin 16
S100A7	0	2.897074806	0	S100 calcium binding protein A7
KRT17	0	2.748031387	0	keratin 17
FABP5	1.0379E-272	2.426823834	2.7209E-268	fatty acid binding protein 5
GJB2	0	2.280668999	0	gap junction protein beta 2
DSC2	1.0042E-224	2.075991132	2.6325E-220	desmocollin 2
CALML3	0	1.908194097	0	calmodulin like 3
SPRR1B	2.3824E-180	1.828244604	6.2456E-176	small proline rich protein 1B
S100A2	3.2136E-153	1.80966861	8.4247E-149	S100 calcium binding protein A2
PI3	1.8405E-178	1.729183139	4.8251E-174	peptidase inhibitor 3
GJB6	0	1.529250761	0	gap junction protein beta 6
COL3A1	5.1566E-207	1.512937505	1.3519E-202	collagen type III alpha 1 chain
SPRR2A	3.9649E-172	1.46296883	1.0394E-167	small proline rich protein 2A
IVL	9.3909E-199	1.462579476	2.4619E-194	involucrin
TYMP	1.2389E-257	1.422061941	3.2479E-253	thymidine phosphorylase
SERPINB3	0	1.401716011	0	serpin family B member 3
CNFN	6.2572E-136	1.390766302	1.6404E-131	cornifelin
COL1A2	1.0576E-182	1.334461813	2.7725E-178	collagen type I alpha 2 chain
SERPINB4	5.1425E-306	1.311588863	1.3482E-301	serpin family B member 4
SLPI	1.65347E-76	1.298328213	4.33473E-72	secretory leukocyte peptidase inhibitor
SERPINB13	0	1.25286817	0	serpin family B member 13
RPS4Y1	0	1.221779084	0	ribosomal protein S4 Y-linked 1
ERO1A	2.0313E-115	1.215386273	5.3254E-111	endoplasmic reticulum oxidoreductase 1 alpha
CD24	4.5276E-198	1.200855571	1.187E-193	CD24 molecule
COL1A1	1.2032E-172	1.196780861	3.1543E-168	collagen type I alpha 1 chain
CSTB	2.7365E-150	1.129141447	7.1741E-146	cystatin B
CLCA2	3.0057E-183	1.108958399	7.8798E-179	chloride channel accessory 2
CALML5	7.32772E-66	1.040492688	1.92103E-61	calmodulin like 5
NEAT1	3.11719E-95	1.034677642	8.17204E-91	nuclear paraspeckle assembly transcript 1
SERPINB1	1.3981E-201	1.016753332	3.6653E-197	serpin family B member 1
CTSB	5.2259E-293	0.999667545	1.37E-288	cathepsin B
GPX3	3.14351E-77	0.9980128	8.24103E-73	glutathione peroxidase 3
DSG3	9.2887E-136	0.969483677	2.4351E-131	desmoglein 3
NDRG1	6.90792E-85	0.960033594	1.81098E-80	N-myc downstream regulated 1
C10orf99	3.2038E-239	0.95189952	8.3992E-235	chromosome 10 open reading frame 99
FAM83A	5.1579E-230	0.941447852	1.3522E-225	family with sequence similarity 83 member A
CDH3	2.2235E-246	0.941363054	5.8291E-242	cadherin 3
UPP1	5.4659E-95	0.927583125	1.43294E-90	uridine phosphorylase 1
TNC	2.2329E-206	0.927178529	5.8539E-202	tenascin C
IGFBP2	1.058E-123	0.895043096	2.7738E-119	insulin like growth factor binding protein 2
QSOX1	7.204E-180	0.884584076	1.8886E-175	quiescin sulfhydryl oxidase 1
VMP1	8.74E-147	0.870245515	2.2913E-142	vacuole membrane protein 1
CXCL8	4.4486E-90	0.856461156	1.16624E-85	C-X-C motif chemokine ligand 8
TXNDC17	1.2907E-105	0.851058267	3.3836E-101	thioredoxin domain containing 17
SDC1	1.26957E-89	0.848007506	3.3283E-85	syndecan 1
MPZL2	2.0436E-130	0.844300329	5.3576E-126	myelin protein zero like 2
PKM	2.4398E-118	0.834773183	6.3961E-114	pyruvate kinase M1/2
SCD	6.5347E-263	0.834664963	1.7131E-258	stearoyl-CoA desaturase
NRIP1	4.0509E-268	0.831329699	1.062E-263	nuclear receptor interacting protein 1
KRT14	3.3623E-94	0.822057645	8.8146E-90	keratin 14
FAM129B	1.0788E-165	0.81640218	2.8281E-161	niban apoptosis regulator 2
CA2	1.0833E-120	0.813555455	2.84E-116	carbonic anhydrase 2
STEAP4	1.0832E-219	0.810766065	2.8398E-215	STEAP4 metalloreductase
MALL	4.4186E-244	0.809841474	1.1584E-239	mal, T cell differentiation protein like
SPARC	2.93315E-79	0.799947428	7.68955E-75	secreted protein acidic and cysteine rich
S100A7A	4.8647E-158	0.790716881	1.2753E-153	S100 calcium binding protein A7A
PITX1	3.284E-248	0.784527027	8.6094E-244	paired like homeodomain 1
SBSN	1.28292E-29	0.77669649	3.3633E-25	suprabasin
JUP	1.86221E-89	0.772403908	4.88197E-85	junction plakoglobin
ACTN1	8.4202E-205	0.767275153	2.2075E-200	actinin alpha 1
MXD1	4.41666E-66	0.766251637	1.15787E-61	MAX dimerization protein 1
MYH9	1.6175E-152	0.765471248	4.2405E-148	myosin heavy chain 9
GJA1	6.27597E-50	0.75363845	1.64531E-45	gap junction protein alpha 1
ITGA6	1.57776E-28	0.752515411	4.13625E-24	integrin subunit alpha 6
HIF1A	2.4595E-158	0.749072463	6.4479E-154	hypoxia inducible factor 1 subunit alpha
ITGB6	1.6171E-242	0.744262877	4.2395E-238	integrin subunit beta 6
CLIP1	2.9497E-131	0.743714065	7.7329E-127	CAP-Gly domain containing linker protein 1
F3	2.7413E-139	0.73895578	7.1865E-135	coagulation factor III, tissue factor

RHCG	1.4144E-122	0.738704028	3.708E-118	Rh family C glycoprotein
IGFBP6	1.2606E-99	0.728376707	3.30471E-95	insulin like growth factor binding protein 6
CRABP2	6.4017E-122	0.728091237	1.6783E-117	cellular retinoic acid binding protein 2
PALLD	2.6328E-190	0.720840104	6.902E-186	palladin, cytoskeletal associated protein
TPM4	2.3186E-137	0.714734472	6.0785E-133	tropomyosin 4
CTSL	1.5796E-144	0.713199884	4.1411E-140	cathepsin L
LUM	3.40419E-85	0.712640816	8.92441E-81	lumican
IL13RA1	2.9469E-228	0.707204477	7.7257E-224	interleukin 13 receptor subunit alpha 1
PGAM1	3.1276E-145	0.691540414	8.1993E-141	phosphoglycerate mutase 1
GRN	5.1556E-117	0.682665985	1.3516E-112	granulin precursor
CARHSP1	1.7594E-181	0.680378372	4.6124E-177	calcium regulated heat stable protein 1
ENAH	6.5747E-132	0.67288014	1.7236E-127	ENAH actin regulator
TMEM54	3.5365E-141	0.668555226	9.2714E-137	transmembrane protein 54
IFI27	6.61553E-91	0.668333857	1.73433E-86	interferon alpha inducible protein 27
PKP1	6.19821E-32	0.667310736	1.62492E-27	plakophilin 1
FLNB	1.3734E-104	0.66230129	3.6004E-100	filamin B
PTBP3	6.4991E-89	0.660345417	1.7038E-84	polypyrimidine tract binding protein 3
NDUFA4L2	1.7053E-86	0.653896587	4.47062E-82	NDUFA4 mitochondrial complex associated like 2
MBOAT2	1.0473E-212	0.645495025	2.7456E-208	membrane bound O-acyltransferase domain containing 2
LTB4R	5.0641E-106	0.644146522	1.3276E-101	leukotriene B4 receptor
RAB31	2.3501E-267	0.637838771	6.1609E-263	RAB31, member RAS oncogene family
CTSC	4.1152E-192	0.637189246	1.0788E-187	cathepsin C
S100A16	8.5235E-102	0.63642095	2.23453E-97	S100 calcium binding protein A16
SEMA4B	1.2392E-167	0.630137177	3.2486E-163	semaphorin 4B
LAD1	2.4608E-108	0.626857875	6.4513E-104	ladinin 1
CASP14	2.92109E-48	0.624478905	7.65794E-44	caspase 14
ENO1	2.53938E-87	0.623975897	6.65724E-83	enolase 1
CSTA	1.65074E-33	0.61861745	4.32759E-29	cystatin A
GSTP1	6.16359E-89	0.618124963	1.61585E-84	glutathione S-transferase pi 1
COL5A2	2.4752E-125	0.615532114	6.489E-121	collagen type V alpha 2 chain
IFITM2	1.6139E-130	0.607536717	4.231E-126	interferon induced transmembrane protein 2
CAPNS1	1.2915E-204	0.607477419	3.3859E-200	calpain small subunit 1
FN1	7.2135E-104	0.607153584	1.8911E-99	fibronectin 1
APP	4.9772E-120	0.606602648	1.3048E-115	amyloid beta precursor protein
RPL13A	4.8754E-132	0.603820194	1.2781E-127	ribosomal protein L13a
DUOXA1	3.2895E-123	0.601397214	8.6238E-119	dual oxidase maturation factor 1
EPS8L1	3.6704E-101	0.599147239	9.62241E-97	EPS8 like 1
LTBP1	1.2325E-237	0.598514566	3.2311E-233	latent transforming growth factor beta binding protein 1
SPINK5	4.92845E-36	0.596823647	1.29204E-31	serine peptidase inhibitor, Kazal type 5
FAT1	2.8501E-168	0.587427253	7.4719E-164	FAT atypical cadherin 1
CD59	2.0835E-120	0.587207232	5.4621E-116	CD59 molecule (CD59 blood group)
ST14	2.5587E-160	0.584651899	6.7078E-156	suppression of tumorigenicity 14
PLCG2	2.41491E-33	0.583949392	6.33092E-29	phospholipase C gamma 2
KRT5	1.12637E-46	0.582577252	2.95288E-42	keratin 5
NECTIN1	7.55836E-74	0.577380672	1.9815E-69	nectin cell adhesion molecule 1
COL12A1	1.3143E-112	0.57734201	3.4455E-108	collagen type XII alpha 1 chain
COL6A3	6.07285E-46	0.576249077	1.59206E-41	collagen type VI alpha 3 chain
FSCN1	6.4865E-100	0.572499802	1.70049E-95	fascin actin-bundling protein 1
A2ML1	1.54206E-68	0.570629699	4.04266E-64	alpha-2-macroglobulin like 1
LYPD3	9.37015E-36	0.570087268	2.45648E-31	LY6/PLAUR domain containing 3
IFI16	5.16178E-84	0.568833915	1.35321E-79	interferon gamma inducible protein 16
THBS2	1.72578E-95	0.567854342	4.5243E-91	thrombospondin 2
GRHL1	4.3309E-67	0.556875487	1.13539E-62	grainyhead like transcription factor 1
TGM1	3.727E-137	0.556673927	9.7708E-133	transglutaminase 1
PROM2	1.0216E-125	0.550750907	2.6781E-121	prominin 2
MAP4	1.0494E-100	0.54930356	2.75103E-96	microtubule associated protein 4
TRPS1	1.9992E-221	0.547971033	5.2412E-217	transcriptional repressor GATA binding 1
EREG	3.4065E-106	0.54774278	8.9305E-102	epiregulin
IL4R	4.9273E-194	0.543244862	1.2917E-189	interleukin 4 receptor
HSPB1	7.10555E-82	0.542379109	1.86279E-77	heat shock protein family B (small) member 1
TMEM45A	8.96434E-51	0.54193676	2.35009E-46	transmembrane protein 45A
YWHAZ	1.55617E-66	0.541887579	4.07966E-62	tyrosine 3-monooxygenase/tryptophan 5-monooxygenase activation protein zeta
SLC2A1	8.61452E-56	0.54099482	2.25838E-51	solute carrier family 2 member 1
KLK10	1.47516E-39	0.540703194	3.86728E-35	kallikrein related peptidase 10
PYGL	1.2267E-125	0.536083069	3.2159E-121	glycogen phosphorylase L
GPRC5A	5.5861E-118	0.533925466	1.4645E-113	G protein-coupled receptor class C group 5 member A
C12orf75	1.85104E-85	0.533912598	4.8527E-81	chromosome 12 open reading frame 75
GALNT6	1.0542E-226	0.533335073	2.7637E-222	polypeptide N-acetylgalactosaminyltransferase 6
PDZK1IP1	2.467E-117	0.533022602	6.4674E-113	PDZK1 interacting protein 1
MYO1B	5.285E-190	0.532339214	1.3855E-185	myosin IB
PSAP	1.82064E-91	0.525567889	4.77299E-87	prosaposin
RHOD	7.9071E-134	0.524768226	2.0729E-129	ras homolog family member D
CAP1	2.7235E-122	0.521406621	7.1401E-118	cyclase associated actin cytoskeleton regulatory protein 1
MTPN	1.8371E-105	0.518355339	4.8163E-101	myotrophin
PKP3	3.68142E-67	0.51734673	9.6512E-63	plakophilin 3
KCNK6	1.9464E-115	0.515762201	5.1028E-111	potassium two pore domain channel subfamily K member 6

LAMP1	1.05629E-84	0.515386157	2.76916E-80	lysosomal associated membrane protein 1
COL6A1	2.76743E-24	0.513885919	7.2551E-20	collagen type VI alpha 1 chain
PLAU	1.192E-115	0.511121675	3.125E-111	plasminogen activator, urokinase
ALDH3B2	4.9162E-130	0.509104749	1.2888E-125	aldehyde dehydrogenase 3 family member B2
KIFC3	2.7333E-175	0.506727691	7.1655E-171	kinesin family member C3
DUOX1	1.3974E-125	0.504991571	3.6634E-121	dual oxidase 1
SERINC2	1.1961E-173	0.502739161	3.1356E-169	serine incorporator 2
IFITM3	9.55848E-46	0.501357384	2.50585E-41	interferon induced transmembrane protein 3
NPEPPS	8.35457E-32	0.500285518	2.19023E-27	aminopeptidase puromycin sensitive
AMD1	7.61061E-46	-0.503059562	1.9952E-41	adenosylmethionine decarboxylase 1
PPP1R15A	2.80135E-90	-0.503277779	7.34402E-86	protein phosphatase 1 regulatory subunit 15A
AL118516.1	3.08899E-58	-0.503345385	8.0981E-54	
SMG1	1.55899E-27	-0.504435667	4.08706E-23	SMG1 nonsense mediated mRNA decay associated PI3K related kinase
NOP53	1.71874E-89	-0.506625513	4.50586E-85	NOP53 ribosome biogenesis factor
CYLD	3.49656E-22	-0.507200598	9.16658E-18	CYLD lysine 63 deubiquitinase
NACA	1.4933E-118	-0.50735502	3.9147E-114	nascent polypeptide associated complex subunit alpha
RPS23	1.8009E-103	-0.511577286	4.7211E-99	ribosomal protein S23
TIPARP	1.23487E-45	-0.512692306	3.23734E-41	TCDD inducible poly(ADP-ribose) polymerase
ARL4A	4.69125E-41	-0.513723506	1.22986E-36	ADP ribosylation factor like GTPase 4A
HLA-B	2.84887E-94	-0.514682676	7.46859E-90	major histocompatibility complex, class I, B
RPL5	2.5787E-113	-0.515292374	6.7603E-109	ribosomal protein L5
RPL13	5.2522E-121	-0.515499309	1.3769E-116	ribosomal protein L13
BIRC3	2.20169E-41	-0.517746955	5.77195E-37	baculoviral IAP repeat containing 3
TGIF1	2.22137E-45	-0.517847072	5.82354E-41	TGFB induced factor homeobox 1
CYP3A5	1.7742E-38	-0.518722248	4.65124E-34	cytochrome P450 family 3 subfamily A member 5
RPS7	2.5489E-118	-0.519834836	6.6823E-114	ribosomal protein S7
RPL14	2.8495E-126	-0.520392715	7.4701E-122	ribosomal protein L14
HSPA2	9.7506E-54	-0.521215822	2.55622E-49	heat shock protein family A (Hsp70) member 2
EPHB6	3.82643E-56	-0.521960188	1.00314E-51	EPH receptor B6
IGFBP3	2.50514E-15	-0.523466113	6.56748E-11	insulin like growth factor binding protein 3
TRA2B	2.16823E-48	-0.523870343	5.68424E-44	transformer 2 beta homolog
RPS15	2.0699E-148	-0.524040075	5.4264E-144	ribosomal protein S15
RPL12	2.1763E-113	-0.525178887	5.7054E-109	ribosomal protein L12
SPAG9	2.97484E-24	-0.529473772	7.79885E-20	sperm associated antigen 9
LAMTOR4	2.51688E-40	-0.530172226	6.59826E-36	late endosomal/lysosomal adaptor, MAPK and MTOR activator 4
SRSF7	4.99335E-53	-0.533200897	1.30906E-48	serine and arginine rich splicing factor 7
BBC3	2.18554E-58	-0.53443879	5.72961E-54	BCL2 binding component 3
RPL35A	3.7223E-125	-0.537009186	9.7584E-121	ribosomal protein L35a
IER2	2.07759E-58	-0.538278039	5.4466E-54	immediate early response 2
BAZ1A	2.24847E-35	-0.543177482	5.8946E-31	bromodomain adjacent to zinc finger domain 1A
MT-ND2	2.82569E-94	-0.545762973	7.40782E-90	MTND2
HERPUD1	8.0729E-56	-0.546548655	2.11639E-51	homocysteine inducible ER protein with ubiquitin like domain 1
WEE1	1.03541E-59	-0.546724387	2.71443E-55	WEE1 G2 checkpoint kinase
CSRNP1	7.50119E-62	-0.551208405	1.96651E-57	cysteine and serine rich nuclear protein 1
MT1E	1.71376E-60	-0.553080687	4.49278E-56	metallothionein 1E
RPLP1	3.1927E-126	-0.555415226	8.3699E-122	ribosomal protein lateral stalk subunit P1
HNRNPDL	4.34541E-82	-0.557859504	1.13919E-77	heterogeneous nuclear ribonucleoprotein D like
TAF4B	9.03134E-52	-0.558165076	2.36766E-47	TATA-box binding protein associated factor 4b
IRF1	6.00545E-65	-0.561539618	1.57439E-60	interferon regulatory factor 1
ZFP36L1	7.27826E-81	-0.563914006	1.90807E-76	ZFP36 ring finger protein like 1
FUS	2.45439E-43	-0.566638918	6.43443E-39	FUS RNA binding protein
RPL11	5.3395E-164	-0.568171477	1.3998E-159	ribosomal protein L11
ALDH3A2	2.91665E-50	-0.569563046	7.6463E-46	aldehyde dehydrogenase 3 family member A2
SAP18	5.93495E-87	-0.571807481	1.55591E-82	Sin3A associated protein 18
RPL32	2.528E-149	-0.572215302	6.6275E-145	ribosomal protein L32
KLF10	2.14533E-57	-0.572720888	5.6242E-53	Kruppel like factor 10
RPL34	3.2635E-141	-0.573578525	8.5557E-137	ribosomal protein L34
RND3	1.3735E-58	-0.575321899	3.60078E-54	Rho family GTPase 3
ERF	8.62799E-39	-0.576483355	2.26191E-34	ETS2 repressor factor
RPS13	1.026E-119	-0.57655464	2.6897E-115	ribosomal protein S13
RHOB	7.90323E-73	-0.576907674	2.07191E-68	ras homolog family member B
LONRF1	7.5715E-59	-0.577236648	1.98495E-54	LON peptidase N-terminal domain and ring finger 1
PTMA	2.4218E-167	-0.577923119	6.3491E-163	prothymosin alpha
RPS15A	2.9567E-119	-0.582027197	7.7514E-115	ribosomal protein S15a
GATA3	1.5441E-38	-0.583754759	4.04801E-34	GATA binding protein 3
EIF1	3.26E-167	-0.584956995	8.5464E-163	eukaryotic translation initiation factor 1
H2AFZ	2.24752E-84	-0.585474343	5.89211E-80	H2A.Z variant histone 1
EGR1	3.25742E-63	-0.585984795	8.53966E-59	early growth response 1
FAU	8.346E-162	-0.587592001	2.188E-157	FAU ubiquitin like and ribosomal protein S30 fusion
FOXQ1	1.15217E-67	-0.58765047	3.02054E-63	forkhead box Q1
BCL6	3.96222E-56	-0.587718741	1.03874E-51	BCL6 transcription repressor
ZFP36	2.00434E-98	-0.589537299	5.25458E-94	ZFP36 ring finger protein
JUND	6.691E-120	-0.594603407	1.7541E-115	JunD proto-oncogene, AP-1 transcription factor subunit
CEBPD	2.73447E-95	-0.595117329	7.16868E-91	CCAAT enhancer binding protein delta
H2AFX	2.00267E-61	-0.595543146	5.25019E-57	H2A.X variant histone
TAF1D	7.32107E-50	-0.596530253	1.91929E-45	TATA-box binding protein associated factor, RNA polymerase I subunit D

TRA2A	1.17837E-45	-0.598537461	3.08921E-41	transformer 2 alpha homolog
B2M	1.178E-152	-0.609149053	3.0882E-148	beta-2-microglobulin
ZFAS1	1.98E-102	-0.609451975	5.19067E-98	ZNFX1 antisense RNA 1
RPS18	3.9912E-155	-0.611428196	1.0463E-150	ribosomal protein S18
CKS2	5.05715E-57	-0.613162212	1.32578E-52	CDC28 protein kinase regulatory subunit 2
RPS21	1.0289E-153	-0.613198353	2.6974E-149	ribosomal protein S21
EZR	8.72992E-72	-0.614152161	2.28864E-67	ezrin
JUN	2.04417E-90	-0.620944506	5.359E-86	Jun proto-oncogene, AP-1 transcription factor subunit
ID4	7.66846E-69	-0.621421316	2.01036E-64	inhibitor of DNA binding 4, HLH protein
DUSP1	9.01973E-73	-0.622720914	2.36461E-68	dual specificity phosphatase 1
MGP	2.79146E-09	-0.628594634	7.3181E-05	matrix Gla protein
RPS12	1.1222E-152	-0.633884307	2.9419E-148	ribosomal protein S12
TXNIP	1.52151E-69	-0.636534656	3.9888E-65	thioredoxin interacting protein
SNHG12	8.67738E-38	-0.638087138	2.27486E-33	small nucleolar RNA host gene 12
ZC3H12A	2.20171E-62	-0.644168223	5.77201E-58	zinc finger CCCH-type containing 12A
SNHG8	1.53465E-95	-0.650966871	4.02325E-91	small nucleolar RNA host gene 8
HEXIM1	6.31915E-55	-0.652117924	1.65663E-50	HEXIM P-TEFb complex subunit 1
SQSTM1	5.85811E-76	-0.655564476	1.53576E-71	sequestosome 1
PER1	3.63447E-66	-0.656886599	9.52814E-62	period circadian regulator 1
SERTAD1	1.55283E-67	-0.663936262	4.07089E-63	SERTA domain containing 1
BMP2	4.8612E-84	-0.669641156	1.27441E-79	bone morphogenetic protein 2
RPL41	1.4896E-186	-0.673478412	3.9051E-182	ribosomal protein L41
PMAIP1	9.9832E-72	-0.675287393	2.6172E-67	phorbol-12-myristate-13-acetate-induced protein 1
ZNF217	1.78883E-39	-0.678509728	4.68959E-35	zinc finger protein 217
RORA	2.02587E-72	-0.680198494	5.31102E-68	RAR related orphan receptor A
RPL36A	3.8852E-114	-0.681050076	1.0185E-109	ribosomal protein L36a
RPL30	6.5658E-151	-0.681430096	1.7213E-146	ribosomal protein L30
MYC	3.6984E-89	-0.682483069	9.69572E-85	MYC proto-oncogene, bHLH transcription factor
HNRNPH1	1.09364E-91	-0.691642797	2.8671E-87	heterogeneous nuclear ribonucleoprotein H1
MT4	4.80942E-62	-0.695511157	1.26084E-57	metallothionein 4
TSC22D3	2.53225E-73	-0.698759401	6.63854E-69	TSC22 domain family member 3
KLF4	1.0214E-126	-0.715378226	2.6776E-122	Kruppel like factor 4
DMKN	1.46156E-48	-0.716486886	3.83162E-44	dermokine
MT1G	3.67466E-45	-0.722558128	9.63349E-41	metallothionein 1G
RPS8	1.7479E-199	-0.724541052	4.5822E-195	ribosomal protein S8
CYR61	1.28589E-56	-0.729461407	3.3711E-52	cellular communication network factor 1
HES1	1.31093E-44	-0.730055108	3.43673E-40	hes family bHLH transcription factor 1
RPL26	6.6482E-173	-0.735584919	1.7429E-168	ribosomal protein L26
BRD2	1.79774E-93	-0.738793145	4.71295E-89	bromodomain containing 2
FOSB	4.2254E-105	-0.746290065	1.1077E-100	FosB proto-oncogene, AP-1 transcription factor subunit
GADD45B	8.31613E-92	-0.748383577	2.18016E-87	growth arrest and DNA damage inducible beta
RSRP1	5.0941E-120	-0.756372179	1.3355E-115	arginine and serine rich protein 1
LGALS3	2.7382E-138	-0.760568414	7.1785E-134	galectin 3
RPS26	5.577E-136	-0.778813344	1.4621E-131	ribosomal protein S26
PNRC1	1.5331E-176	-0.779350197	4.0191E-172	proline rich nuclear receptor coactivator 1
JUNB	1.2714E-156	-0.793806612	3.3332E-152	JunB proto-oncogene, AP-1 transcription factor subunit
INTS6	6.57809E-81	-0.795123213	1.72451E-76	integrator complex subunit 6
BTG1	2.4779E-149	-0.806175918	6.4961E-145	BTG anti-proliferation factor 1
NRARP	2.69063E-73	-0.808098065	7.05376E-69	NOTCH regulated ankyrin repeat protein
ID3	5.31406E-98	-0.811359159	1.39313E-93	inhibitor of DNA binding 3, HLH protein
TLE4	5.19912E-86	-0.812287035	1.363E-81	TLE family member 4, transcriptional corepressor
PPP1R10	8.28704E-88	-0.817260224	2.17253E-83	protein phosphatase 1 regulatory subunit 10
ERRFI1	3.40866E-87	-0.836686914	8.93613E-83	ERBB receptor feedback inhibitor 1
MTRNR2L12	1.33E-111	-0.839168233	3.4866E-107	MT-RNR2 like 12
CLDN1	3.88829E-93	-0.83934147	1.01935E-88	claudin 1
CXCL14	7.9817E-121	-0.839839757	2.0925E-116	C-X-C motif chemokine ligand 14
IFRD1	3.8364E-108	-0.848837181	1.0058E-103	interferon related developmental regulator 1
TNFAIP3	9.4983E-134	-0.850253048	2.4901E-129	TNF alpha induced protein 3
SERPINB2	1.01258E-76	-0.853793054	2.65457E-72	serpin family B member 2
MT1X	4.0301E-127	-0.872980474	1.0565E-122	metallothionein 1X
RPS4X	1.8621E-234	-0.883974019	4.8817E-230	ribosomal protein S4 X-linked
MFSD2A	3.7304E-133	-0.886994315	9.7796E-129	major facilitator superfamily domain containing 2A
DNAJA1	1.5178E-152	-0.893516447	3.979E-148	DnaJ heat shock protein family (Hsp40) member A1
SYT8	1.95676E-84	-0.89855341	5.12983E-80	synaptotagmin 8
CCND1	3.4308E-108	-0.899759398	8.9941E-104	cyclin D1
NR4A1	1.8102E-126	-0.916004374	4.7455E-122	nuclear receptor subfamily 4 group A member 1
GADD45A	7.21647E-97	-0.9844507	1.89187E-92	growth arrest and DNA damage inducible alpha
ADRB2	6.6723E-134	-1.02940421	1.7492E-129	adrenoceptor beta 2
ATF3	8.5398E-175	-1.040787576	2.2388E-170	activating transcription factor 3
RPS10	4.3674E-201	-1.070297561	1.145E-196	ribosomal protein S10
NFKBIA	8.4109E-267	-1.25550235	2.205E-262	NFKB inhibitor alpha
KRT15	5.89049E-24	-1.297195822	1.54425E-19	keratin 15
LGALS7B	2.1123E-112	-1.309599472	5.5375E-108	galectin 7
XIST	8.4249E-269	-1.322304647	2.2087E-264	X inactive specific transcript
CFD	3.97929E-44	-1.359117884	1.04321E-39	complement factor D
APOE	3.3908E-173	-1.398278844	8.8894E-169	apolipoprotein E

Table S6

CCL27	1.3404E-166	-1.800913472	3.514E-162	C-C motif chemokine ligand 27

Table S7: Demographics of subjects with chronic wounds

ID	Age	Gender	BMI	Ethnicity	wound location
W1	58	Female	23.7	Caucasian	coccyx
W2	39	Male	23.2	Caucasian	sacrum
W3	60	Female	28	Caucasian	sternum
W4	54	Female	37.1	Caucasian	abdomen
W5	31	Female	41.79	Caucasian	left axilla
W6	33	Male	27.8	Caucasian	abdomen
W7	58	Female	40.3	Caucasian	abdomen
W8	51	Male	22.7	Caucasian	sacrum
W9	58	Male	26.4	African American	inner right thigh
W10	78	Male	22.2	Caucasian	sacrum
W11	49	Male	33.3	Not available	chest
W12	46	Female	30.7	African American	right pelvis
Sc_W1	39	Male	15.8	Caucasian	ischial Tuberosity
Sc_W2	69	Male	34.3	African American	right heel
Sc_W3	42	Female	29.7	Caucasian	right ischial

Table S8: Primers used for this study

Primer	Sequence
h_TP53_F	5' - GAA CAA GTT GGC CTG CAC TG - 3'
h_TP53_R	5' - GAA GTG GGC CCC TAC CTA GA - 3'
h_P73_F	5' - CTG TCA TCC CCT CCA ACA CC - 3'
h_P73_R	5' - ACG TGC TCC GCT TTC TTG TA - 3'
h_HDAC1_FP	5'-TGT CGG AGT ACA GCA AGC AG-3'
h_HDAC1_RP	5'-TGC CTC GGA CTT CTT TGC AT-3'
h_SIRT1_FP	5'- CGC TGG CCG ACA ACT TGT A- 3'
h_SIRT1_RP	5'-CAT GTG AGG CTC TAT CCT CCT-3'
h_18S_FP	5'-GTA ACC CGT TGA ACC CCA TT-3'
h_18S_RP	5'-CCA TCC AAT CGG TAG TAG CG-3'
